# The effect of African ancestry and mismatch-repair enzyme deficiency/microsatellite instability-high on colorectal adenocarcinoma immune gene expression

**DOI:** 10.3389/fgstr.2025.1638438

**Published:** 2025-10-03

**Authors:** Dimitri F. Joseph, Andrew Fu, Ricardo E. Flores, Dev V. Sharma, Joseph F. LaComb, Julie M. Clark, Ellen Li, Yunhan Liao, Jie Yang, Qi Yu, Seidu Adams, Olorunseun O. Ogunwobi, Brian Theisen, Nina G. Steele, Bin Chen, Alexandra Guillaume

**Affiliations:** ^1^ Department of Pharmacology and Toxicology, Michigan State University, East Lansing, MI, United States; ^2^ Department of Medicine, Renaissance School of Medicine at Stony Brook University, Stony Brook, NY, United States; ^3^ Department of Surgery, Henry Ford Pancreatic Cancer Center, Detroit, MI, United States; ^4^ Biostatistics Shared Resource at Stony Brook Cancer Center, Stony Brook, NY, United States; ^5^ Department of Family, Population and Preventive Medicine, Renaissance School of Medicine at Stony Brook University, Stony Brook, NY, United States; ^6^ Department of Medicine, NYC Health + Hospitals/Kings County Hospital Center, Brooklyn, NY, United States; ^7^ Department of Medicine, State University at New York (SUNY) Downstate Health Sciences University, Brooklyn, NY, United States; ^8^ Department of Biochemistry and Molecular Biology, Michigan State University, East Lansing, MI, United States; ^9^ Department of Pathology, Henry Ford Health, Detroit, MI, United States

**Keywords:** colorectal neoplasm, DNA mismatch repair, African continental ancestry group, European continental ancestry group, gene expression

## Abstract

**Background:**

Previous analyses of bulk colon and rectal adenocarcinoma (COAD/READ) RNA-sequence data comparing African ancestry (AA) and European ancestry (EA) groups have reported differentially expressed genes related to the immune response. However, these previous analyses of AA versus EA tissues did not control for mismatch-repair enzyme (MMR)/microsatellite instability (MSI) status, which is also associated with altered expression of immune related genes, and is used to determine eligibility for immune checkpoint inhibitor therapy.

**Methods:**

TCGA-COAD-READ bulk RNA-sequence data were analyzed to identify immune related genes that were significantly associated with AA and MMR-deficient (MMR-d)/MSI-High (MSI-H) groups. Reverse transcriptase-quantitative polymerase chain reaction (RT-qPCR) assays for selected immune genes relative to two reference genes, (*C1ORF43* and *RAB7A*) were conducted on an independent set of AA (n = 59) vs. EA (n = 59) formalin-fixed paraffin embedded (FFPE) samples enriched for MMR-d/MSI-H samples. Multiple linear regression models were employed to investigate ancestry and MMR/MSI status while controlling other variables.

**Results:**

Multivariable regression analysis of the TCGA-COAD-READ data revealed that *CXCL10* expression was lower in AA vs. EA groups and higher in MMR-d/MSI-H vs. MMR-proficient (MMR-p)/MSI-Low (MSI-L)+microsatellite stable (MSS) groups while controlling for COAD/READ location and stage. Neither COAD/READ stage or location were significant while controlling for ancestry and MMR/MSI status. CXCL10 is an important chemokine that regulates the tumor immune microenvironment. The number of AA MMR-d/MSI-H samples in the TCGA-COAD-READ dataset was too low (n = 9) to detect a significant effect of AA on *CXCL10* expression across MMR/MSI status. *CXCL10* mRNA levels measured by RT-qPCR in an independent set of COAD FFPE samples enriched for AA MMR-d/MSI-H samples, confirmed that *CXCL10* expression was higher in MMR-d/MSI-H samples compared to MMR-p/MSI-L+MSS, however, differences in *CXCL10* expression between AA vs. EA did not reach significance.

**Discussion:**

These results did not detect significant effects of AA on *CXCL10* expression across MMR/MSI status.

## 1 Introduction

Colorectal adenocarcinoma (COAD/READ) is the second leading cause of all cancer related deaths in the US (1). In the US, the African continental ancestry population group has both a higher incidence and poorer survival for COAD/READ compared with the European ancestry (EA) group (1). Multiple factors including socioeconomic factors that affect access to prevention and early diagnosis of COAD/READ (2) contribute, but do not fully explain these disparities. Previous studies of COAD/READ bulk RNA sequencing data generated from fresh/frozen COAD/READ samples have reported African vs. European ancestry (AA vs. EA) tissues exhibit numerous differentially expressed genes (DEGs), including genes related to the tumor immune microenvironment (3–10). A major limitation of these studies is the small numbers of African ancestry (AA) samples included in these studies. Most of the previous analyses used The Cancer Genome Atlas (TCGA) RNA-sequencing dataset (11), which has the largest but still limited number (n=64) of AA COAD/READ samples, in addition to data generated from smaller sets of 6–15 AA COAD samples.

These previous analyses were conducted without considering mismatch repair enzyme-deficiency/microsatellite-high (MMR-d/MSI-H) status as a potential confounding variable. Universal screening of COAD/READ biopsies and surgical resection pathology specimens for MMR-d/MSI-H status is now routinely conducted at US medical centers. This is because MMR-d/MSI-H patients are more responsive to immune checkpoint inhibitors (12). This may relate to higher expression of immune related genes, such as C-X-C motif chemokine 10 (*CXCL10)* gene (13, 14). MMR-d/MSI-H status is associated with increased cytoplasmic damaged DNA, which triggers the cyclic GMP-AMP synthase - stimulator of interferon genes (c-GAS/STING) pathway. This leads to activation of interferon gamma signaling pathways and activation of the CXCL10-CXCR3 axis, which has been shown to regulate immune cell homing and activation (14, 15).

A univariate analysis of two publicly accessible AA vs. EA bulk RNA sequence datasets revealed that *CXCL10* expression was lower in AA vs. EA groups (16). To test the hypothesis that ancestry could affect *CXCL10* expression levels across MMR/MSI status, the AA vs. EA TCGA-COAD-READ RNA-seq was reanalyzed with a focus on both ancestry and MMR/MSI status. Because COAD samples annotated for both ancestry and MMR/MSI status were not available in commercial tissue banks, we assembled an independent set of 134 AA and EA COAD formalin fixed paraffin embedded (FFPE) samples from three medical centers. This independent cohort included roughly equal numbers of AA MMR-d/MSI-H, AA MMR-p/MSI-L+MSS, EA MMR-d/MSI-H, and EA MMR-p/MSI-L+MSS, because of our focus on African ancestry and MMR-d/MSI-H COAD/READ. This cohort is therefore enriched for self-identified African ancestry and MMR-d/MSI-H because only ~12% of US COAD/READ cases has evidence of African ancestry (17) and only ~15% have MMR-d/MSI-H status (18).

## 2 Methods

### 2.1 Data acquisition and identification of DEGs

The Cancer Genome Atlas Colonic adenocarcinoma (TCGA-COAD) RNA sequence data from AA (n = 64) and EA (n =284) groups was downloaded as unstranded STAR (raw) counts and processed TPM by using TCGAbiolinks R/Bioconductor package (19). The following clinical metadata variables were also downloaded from TCGA: 1.) age at time of collection (years); 2. sex (male/female); self-identified ancestry (African/European); COAD/READ tumor location (right, left; COAD/READ stage (I,II,III,IV); and MSI status. MSI status was available for 327 out of 348 samples (20). For 17 of 21 samples without MSI-status values, the Microsatellite Analysis for Normal Tumor InStability (MANTIS) scores in the TCGA-COAD-READ database were used to categorize MSI status (21). Samples with MANTIS scores < 0.4 were categorized as MMR-p/MSI-L+MSS and scores ≥ 0.4 were categorized as MMR-d/MSI-H. The raw counts of 15 AA and 18 EA COAD samples from the SUNY Stonybrook/Downstate medical centers were downloaded from Gene Expression Omnibus (GEO) with the accession number GSE146009 (7). The COAD data in GSE146009 was downloaded from 15 AA and 18 EA samples, which were annotated with respect to ancestry but not for MMR/MSI status. The raw counts were used as input for identifying differentially expressed genes (DEGs) using edgeR (22). After using edgeR to normalize the raw counts, the resulting “cpm” counts were used as input into the *wilcox.test* function in R (v4.0.2) as previously described (23). The threshold for identifying DEGs was the absolute value│log_2_ fold change│≥ 1 and adjusted p-value <0.05 The consensus molecular subtypes (CMS) labels reported for the TCGA-COAD-READ dataset based on CMS network and CMS Random Forest (RF) were downloaded from cms_labels_public_all.txt - syn4978511 - Files (24). The abundance of tumor associated T-cells was estimated using CIBERSORT analysis of the TCGA-COAD-READ RNA-sequence data (25).

### 2.2 Assembly of COAD FFPE samples from three US medical centers.

Assembly of 134 de-identified adult (age > 18) human COAD/READ FFPE tissue samples archived between 2012 and 2024 from three US medical institutions, Stony Brook University Hospital (Stony Brook, NY), New York City Health + Hospitals (NYCH+H)/Kings County Hospital (KCH, Brooklyn, NY), and Henry Ford Health Center (Detroit, MI) was approved by the Stony Brook Institutional Review Board (sIRB2024-0020) with reliance forms reviewed by the Institutional Review Boards for Henry Ford Health Center and Michigan State University. No reliance form was required for NYCH+H/KCH since the research protocol included an honest broker that oversaw HIPAA compliance, was conducted with waiver of consent and was determined to be not human research by its Institutional Review Board (IRB1949860). Only initial surgical resections of treatment-naïve sporadic COAD-READ (excluding inflammatory bowel disease-associated and hereditary COAD-READ syndromes) were selected for analysis. The samples were linked to deidentified clinical metadata curated from electronic medical records by physicians at each of the three medical centers using a common data dictionary as previously described (26). The variables collected for the metadata included: 1.) age at the time of the sample collection (years), 2.) sex (male/female); 3.) ancestry (AA vs. EA) based on self-identification, 4.) ethnicity (all non-Hispanic); 5.) body mass index (BMI, kg/m^2^); 6.) diabetes mellitus status (type 1 diabetes, type 2 diabetes (T2DM), no diabetes); 7.) smoking (current, former, never); 8.) COAD tumor location (right defined as cecum, ascending colon, hepatic flexure, transverse colon; left defined as including splenic flexure, descending colon, sigmoid, rectum); 9.) COAD stage (1-4); 10.) MMR-d/MSI-H vs. MMR-p/MSI-L+MSS status classified primarily by immunohistochemistry (IHC); 11.) insurance status (Commercial/Medicare; Medicaid Mgd; Medicaid/Self-Pay).

### 2.3 RT-qPCR analysis of COAD-READ FFPE samples.

Total RNA was extracted from 5 µm COAD FFPE curls using the RecoverAll™ Total Nucleic Acid Isolation Kit (Thermo Fisher Scientific Inc, Waltham, MA) according to the manufacturer’s recommendation, except paraffin was removed by xylene washes, the protease digestion was extended to 3 hours at 50°C. 200–500 ng of total bulk RNA was reverse transcribed using Superscript IV™ VILO™ cDNA kit (Thermo Fisher Scientific Inc, Waltham, MA) according to the manufacturer’s recommendation qPCR was performed using the Applied Biosystems QuantStudio 3 Real Time PCR System (Thermo Fisher Scientific Inc., Waltham, MA). The 20 μl dual probe (target immune gene/reference gene) PCR reactions included 1-2 μl cDNA (corresponding to 25 ng of RNA), 1 μl 1× TaqMan Universal PCR master mix, 1 μl for each primers/pre mix (for target and reference gene) The reactions, run in triplicate, were incubated in a 96-well optical plate at 95 °C for 10 min, followed by 40 cycles of 95 °C for 15 s and 60°for 10 min. The threshold cycle (Ct) was defined as the fractional cycle number at which the fluorescence passes the fixed threshold. The Ct data were determined using default threshold settings. Taqman^®^ primer probe set IDs for the target genes were: *CXCL10* (HS00171042), *CD45* (HS04189704) and C*D3D* (HS00174158). The Taqman^®^ primer probe set IDs for the reference genes were: *RAB7A* (HS01115139) and *C1ORF43* (HS00367486). A previous study reported that *RAB7a* and *C1ORF43* were best suited for normalizing RT-qPCR assays of COAD/READ samples (27), particularly because of the low covariance exhibited by these two reference genes. These commercial probe/primer sets have been used extensively in previous publications including the study evaluating the two COAD reference genes (27). The ΔCt were calculated as reference gene Ct – target gene Ct to estimate the log transformation of the ratio of target gene/reference gene templates in the reactions.

### 2.4 Statistical analysis

TCGA *CXCL10* outcomes were expressed as CXCL10 log_2_TPM. The RT-qPCR CXCL10 values were expressed as *C1ORF43* Ct - *CXCL10* Ct and *RAB7A* Ct - *CXCL10* Ct. *CD45* values were expressed as *C1ORF43* Ct – *CD45* Ct and *RAB7A* Ct – *CD45* Ct, and *CD3D* values were expressed as *C1ORF43* Ct – *CD3D* Ct and *RAB7A* Ct – *CD3D* Ct. Spearman’s correlation was used to examine the linear correlation between *CXCL10* and continuous variables such as age, *CD45* and *CD3D* values. The Wilcoxon rank sum test (for variables with 2 levels) or Kruskal-Wallis test (for variables with ≥ 3 levels) was utilized to examine the marginal difference in outcomes among categorical variables. For the Kruskal Wallis test, a Dunn’s *post-hoc* test was used to compare individual groups with each other. Multiple linear regression models were then utilized to examine whether there was a difference in the ancestry level or MMR status after adjusting for COAD/READ location and COAD/READ stage. 134 independent human COAD/READ FFPE tissue samples are expected to have 90% power to detect an increase in R^2^ being 6.5% while the R^2^ of model using covariates alone being 11.5% based on a multiple regression full-versus-reduced-model F-test with a Type I error rate of 0.05 (28). Both R^2^s in the sample size justification are estimated from TCGA data. Statistical analysis was performed using GraphPad Prism 10 (for some univariate analyses), by using the cor.test () function in R version 4.44 to calculate correlation coefficients and SAS 9.4 (SAS Institute Inc., Cary, NC). Significance level was set at 0.05.

## 3 Results

### 3.1 CXCL10 transcript expression is lower in AA vs. EA in two independent COAD-READ tumor bulk RNA-sequence datasets

**Figure 1:**
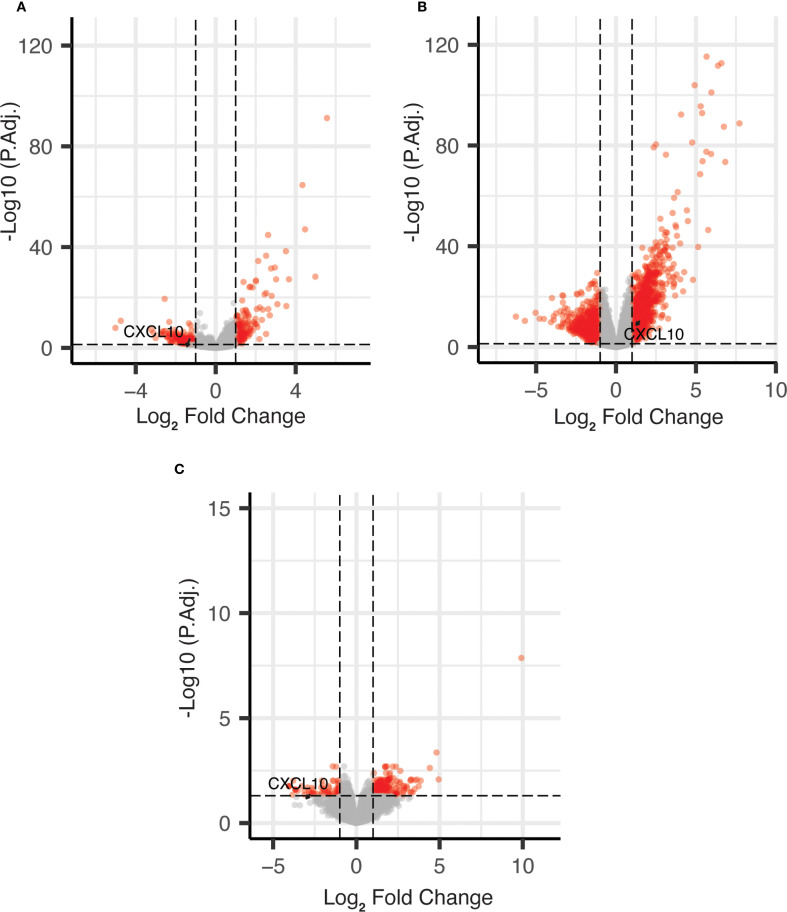
Volcano plots of DEGs for African vs. European ancestry and MMR-d/MSI-H using edgeR. **(A)** African vs. European ancestry DEGs in TCGA COAD dataset; **(B)** MMR-d/MSI-H vs. MMR-p/MSI-L+MSS DEGs in TCGA COAD dataset; **(C)** African vs. European ancestry DEGs in SUNY Downstate/Stony Brook dataset.

As shown in **Figure 1**, *CXCL10* was identified as AA vs. EA differentially expressed genes (DEGs), which were expressed at a lower level in AA vs. EA COAD/READ samples in both the TCGA-COAD-READ (64 AA vs. 284 EA) and a smaller SUNY Downstate/Stony Brook (15 AA vs. 18 EA) bulk RNA sequence datasets, using edgeR (22, see **Supplementary Table S1**).

### 3.2 Categorization of MMR/MSI status between AA and EA TCGA-COAD-READ samples

**Figure 2:**
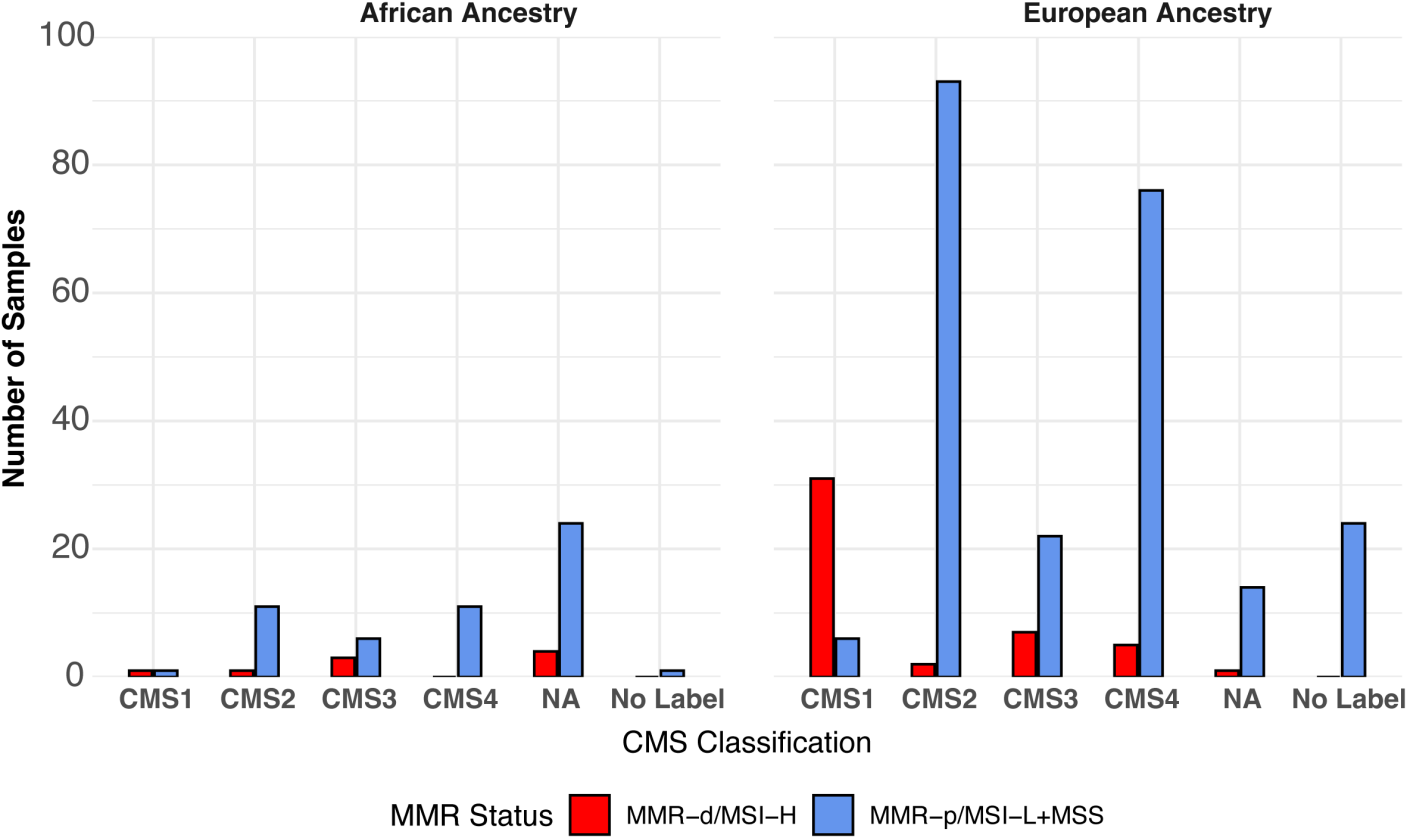
Distribution of CMS labels in TCGA-COAD samples by ancestry and by MMR/MSI status.

MMR/MSI-status of the TCGA-COAD samples was obtained by downloading the TCGA-COAD-READ metadata and the MSI status reported previously for 327/348 samples (20). Of the 21 samples lacking MSI status values, 17 were categorized by using the MANTIS score (21). No information on the MMR/MSI status was provided for the smaller SUNY Downstate/Stony Brook RNA-seq dataset (7). The distribution of consensus molecular subtype (CMS1, CMS2, CMS3, CMS4, No Label) previously assigned to the TCGA-COAD-READ samples (24) for:1.) AA MMR-p/MSI-L+MSS; 2.) AA MMR-d/MSI-H; 3.) AA MMR undetermined; 4.) EA MMR-p/MSI-L+MSS; 5.) EA MMR-d/MSI-H vs. MMR-p/MSIL+MSS are shown in **Figure 2**. CMS1 has been associated with high expression of immune-related genes and MMR-d/MSI-H (22). CMS2 has been associated with a differentiated epithelial cell phenotype. CMS3 has been termed the metabolic subtype because of dysregulated metabolic genes. CMS4 is associated with a high stromal content. Some of the COAD samples could not be readily assigned to a single CMS and have been termed No Label. Thirty-seven (13%) of the 284 EA samples were labeled as CMS1. Thirty-one (84%) of the 37 EA CMS1 samples were also MMR-d/MSI-H. Only two (3%) of 64 AA samples were labeled as CMS1. Only one (50%) of two AA CMS1 samples was also MMR-d/MSI-H.

### Overlap between TCGA-COAD-READ AA vs. EA DEGs and MMR-d/MSI-H vs. MMR-p/MSI-L+MSS DEGs

To reduce the number of false positive DEGs, the Wilcoxon rank sum test was used to determine the overlap between the AA vs. EA DEGs and the MMR-d/MSI-H vs. MMR-p/MSI-L+MSS DEGs (23). The number of AA vs. EA DEGs was reduced to 39 from 420, and the number of MMR-d/MSI-H vs. MMR-p/MSI-L+MSS DEGs was reduced to 738 from 2177 (see **Supplementary Table S1**). The overlap between the 39 AA vs. EA DEGs and the 738 MMR-d/MSI-H vs. MMR-p/MSI-L+MSS DEG lists consists of 7 genes (*CXCL10*, *ALOX15B*, *IDO1*, *HCAR2*, *MARCO*, *OR2I1P* and *MTND4P24*. Six of seven DEGs were decreased in the AA group and increased in the MMR-d/MSI-H group (*CXCL10*, *ALOX15B*, *IDO1*, *HCAR2*, *MARCO* and O*R2I1P*). The first five DEGs have been linked to macrophage function and in some instances with COAD/READ (29–34). *OR2I1P* is a pseudogene with unknown function. *MTND4P24*, which is increased in AA vs. EA and decreased in the MMR-d/MSI-H vs. MMR-p/MSI-L+MSS groups, is a pseudogene with unknown function.

### Analysis of variables affecting CXCL10 mRNA expression in the TCGA-COAD dataset

Further analysis of the effects of AA and MMR-d/MSI-H status focused on *CXCL10* log_2_TPM as the outcome because this gene plays a key role in regulating COAD/READ tumor microenvironment in MMR-d/MSI-H samples (14, 15). Differences in *CXCL10* values were significantly associated with ancestry, MMR/MSI status, COAD/READ location, COAD/READ stage, but not sex (see **Table 1**). Age was not significantly correlated with *CXCL10* levels. Multiple linear regression models were used to examine associations to *CXCL10* expression while adjusting for COAD/READ stage, and with and without COAD/READ location, because of the number of missing location values. As shown in **Table 1**, *CXCL10* values were lower in AA vs. EA (p -value < 0.0001) and higher in MMR-d/MSI-H vs. MMR-p/MSI-L+MSS (p-value < 0.0001), while controlling for COAD/READ stage and location. Neither COAD/READ stage nor location were significant, while controlling for ancestry and MMR/MSI status. Although MMR-d/MSI-H status has been previously correlated with right colon location (35), no significant multicollinearity was detected between the co-variables (results not shown). Estimated differences in *CXCL10* due to ancestry across MMR/MSI status were not significant (see **Table 2**). Consistent with CXCL10’s role as a T-cell attractant was the significant correlation (Spearman’s correlation coefficient r= 0.44, p-value <0.0001) detected between *CXCL10* log*_2_* TPM values and T-cell abundance estimated by CIBERSORT (see **Supplementary Figure S1** **).**

**Table 1:** Univariate analysis of *CXCL10* log_2_TPM values from the TCGA-COAD-READ dataset with co-variables.

Variable	N missing	Level	N	*CXCL10* log_2_ TPM median	IQR	P-value
Ancestry	0	African	64	4.34	2.52	**0.0003**
		European	284	5.27	2.72	
MMR/MSI Status	4	MMR-d/MSI-H	55	6.23	2.33	**<.0001**
		MMR-p/MSI-L+MSS	289	4.77	2.92	
Ancestry by MMR/MSI Status	4	African MMR-d/MSI-H	9	5.38	2.27	**<.0001**
		African MMR-p/MSI-L+MSS	52	3.81	2.71	
		European MMR-d/MSI-H	51	6.75	1.97	
		European MMR-p/MSI-L+MSS	228	4.91	2.82	
Sex	0	Female	168	5.05	2.40	0.4065
		Male	180	5.16	3.04	
COAD/READ Location	22	Left	167	4.78	3.01	0.0108
		Right	159	5.33	3.30	
COAD/READ Stage	12	1	53	5.13	2.18	0.0278
		2	119	5.45	3.00	
		3	113	4.90	3.05	
		4	51	4.62	2.74	

Bold values indicate significant p-values < 0.05.

**Table 2:** Multiple linear regression models of estimated differences in TCGA-COAD *CXCL10* log_2_TPM values with 95% confidence interval (CI).

Effect	Level	Estimated differences in *CXCL10* log_2_TPM (95% CI)	P-value*
With adjustment for COAD/READ location			
Ancestry	African vs European	-1.06 (-1.60, -0.58)	**0.0001**
MMR/MSI Status	MMR-d/MSI-H vs MMR-p/MSI-L+MSS	1.36 (0.70, 2.02)	**<.0001**
COAD/READ Location	Left vs Right	-0.35 (-0.81, 0.09)	0.1289
COAD/READ Stage	2 vs 1	0.06 (-0.58, 0.69)	0.3516
	3 vs 1	-0.16 (-0.81, 0.49)	
	4 vs 1	-0.54 (-1.31, 0.23)	
Without adjustment for COAD/READ location			
Ancestry	African vs European	-0.93 (-1.44, -0.41)	**0.0004**
MMR/MSI Status	MMR-d/MSI-H vs MMR-p/MSI-L+MSS	1.55 (0.98, 2.11)	**<.0001**
COAD/READ Stage	2 vs 1	0.02 (-0.59, 0.62)	0.2914
	3 vs 1	-0.12 (-0.73, 0.50)	
	4 vs 1	-0.58 (-1.31, 0.15)	

*P-values were calculated based on type 3 tests from a multiple linear regression model.

Bold values indicate significant p-values < 0.05.

Exploratory analysis comparing *CXCL10* log_2_TPM values to CMS labels within each ancestry group (see **Supplementary Figure S2**), detected no significant difference between CMS labels within the AA group. In contrast, significant differences were detected between CMS labels in the EA group (p-value <0.0001). Dunns *post-hoc* test detected significantly increased *CXCL10* expression in the EA CMS1 group compared with both CMS2 (p-value <0.0001) and CMS3 groups (p-value<0.0001), but not CMS4 or No Label groups. Also, *CXCL10* log_2_ TPM values were significantly higher in EA CMS4 compared with both CMS2 (p-value<0.0001) and CMS3 (p-value=0.0002) groups, but not CMS1 or No Label groups.

### RT-qPCR results from an independent set of COAD/READ FFPE samples enriched for AA MMR-d/MSI-H COAD samples

**Figure 3:**
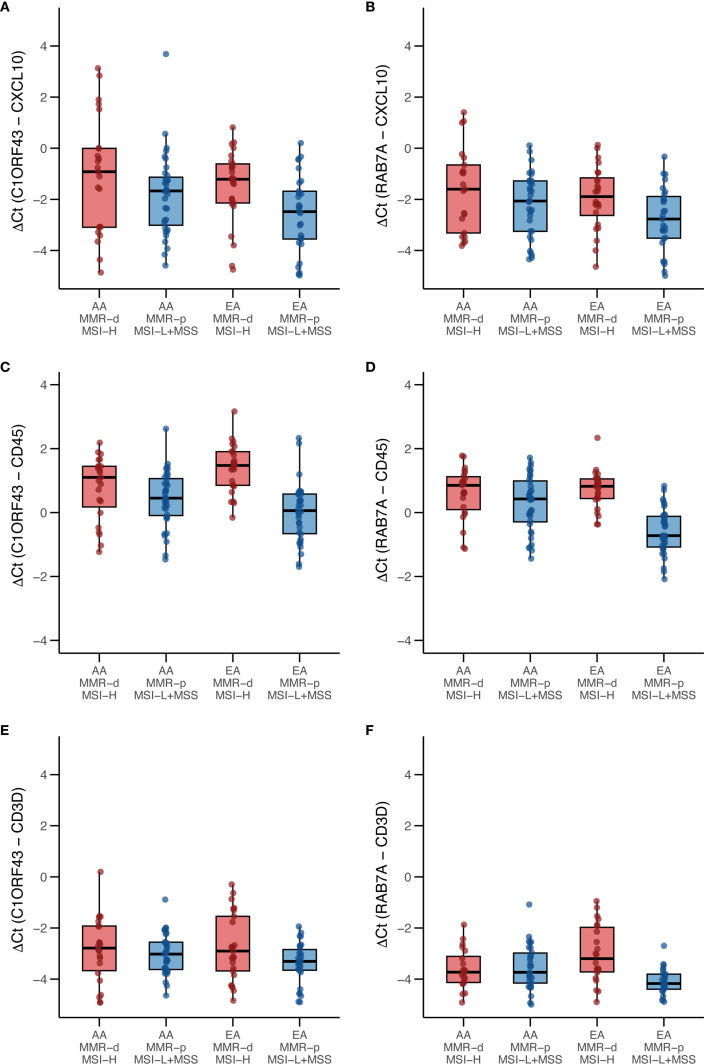
Box and whisker plots of RT-qPCR analysis of CXCL10, CD45 and CD3D expression in an independent set enriched for African ancestry (AA) MMR-d/MSI-H COAD FFPE samples. **(A)** *C1ORF43* Ct - *CXCL10* Ct; **(B)** *RAB7A* Ct – *CXCL10* Ct; **(C)** *C1ORF43* Ct – *CD45* Ct; **(D)** *RAB7A* Ct – *CD45* Ct, **(E)** *C1ORF43* Ct – *CD3D* Ct, **(F)** *RAB7A* Ct – *CD3D* Ct.

Because only 9 of the AA TCGA COAD/READ samples were MMR-d/MSI-H, an independent set of 134 COAD FFPE samples was assembled from three medical centers that was composed of roughly equal numbers of AA MMR-d/MSI-H, AA MMR-p/MSI-L+MSS, EA MMR-d/MSI-H, EA MMR-p/MSI-L+MSS. Amplifiable RNA by RT-qPCR was recovered from 118 (88%) of the samples. *CXCL10* expression was normalized relative to two reference genes as *C1ORF43* Ct – *CXCL10* Ct and *RAB7A* Ct – *CXCL10* Ct (see **Figure 3**). Loss of 12% of the original 134 samples resulted in the expected power being reduced from 90% to 87%. Univariate analyses of both values confirmed that *CXCL10* values were significantly higher in MMR-d/MSI-H vs. MMR-p/MSI-L+MSS (see **Tables 2**, **3**). In contrast to the TCGA-COAD *CXCL10* log_2_ TPM values, the RT-qPCR *CXCL10* values relative to both reference genes trended slightly higher in AA vs. EA but these differences did not reach significance. Differences in *C1ORF43* Ct – *CXCL10* Ct values were associated with COAD/READ location but not with COAD/READ stage, and differences in *RAB7A* Ct – *CXCL10* Ct values were associated with COAD/READ stage but not with COAD/READ location. Neither RT-qPCR *CXCL10* values were significantly associated with age or sex. When the same multivariable model used to analyze the TCGA *CXCL10* log_2_ TPM results was applied to the RT-qPCR results (**Tables 3**–**6**), AA *C1ORF43* Ct *- CXCL10* Ct (p = 0.0438) and *RAB7A* Ct *– CXCL10* Ct *(*p-value = 0.0497*)* values were higher than EA values, MMR-d/MSI-H *C1ORF43* Ct – *CXCL10* Ct (p-value = 0.019) and *RAB7A* Ct - *CXCL10* Ct (p-value = 0.093) values were higher or trended higher than MMR-p/MSI-L+MSS values, while controlling for COAD/READ stage and location.

**Table 3:** Univariate analysis of RT-qPCR *C1ORF43* Ct – *CXCL10* Ct results from an independent set enriched for MMR-d/MSI-H COAD/READ FFPE samples.

Variable	Level	N	C1ORF43 Ct-CXCL10 Ct median	IQR	P-value
Ancestry	African	59	-1.59	2.71	0.0812
	European	59	-2.14	2.91	
MMR/MSI Status	MMR-d/MSI-H	48	-1.13	2.37	**0.0008**
	MMR-p/MSI-L+MSS	70	-2.33	2.28	
Ancestry by MMR/MSI Status	African MMR-d/MSI-H	23	-0.92	3.11	**0.0009**
	African MMR-p/MSI-L+MSS	36	-1.68	2.02	
	European MMR-d/MSI-H	25	-1.21	1.53	
	European MMR-p/MSI-L+MSS	34	-2.99	2.88	
Sex	Female	63	-1.64	2.90	0.5601
	Male	55	-2.00	2.49	
COAD/READ Location	Left	35	-2.33	2.67	**0.0256**
	Right	83	-1.59	2.59	
COAD/READ Stage	1	31	-2.36	2.13	0.1551
	2	39	-1.59	2.31	
	3	39	-1.54	2.87	
	4	9	-2.28	4.52	

Bold values indicate significant p-values < 0.05.

**Table 4:** Univariate analysis of RT-qPCR *RAB7A* Ct - *CXCL1*0 Ct results from an independent set of COAD/READ FFPE samples.

Variable	Level	N	*RAB7A* Ct– *CXCL10* Ct median	IQR	P-value
Ancestry	African	59	-2.05	2.50	0.0812
	European	59	-2.27	2.44	
MMR/MSI Status	MMR-d/MSI-H	48	-1.69	2.16	**0.0088**
	MMR-p/MSI-L+MSS	70	-2.58	2.62	
Ancestry by MMR/MSI Status	African MMR-d/MSI-H	23	-1.60	3.09	**0.0093**
	African MMR-p/MSI-L+MSS	36	-2.08	2.25	
	European MMR-d/MSI-H	25	-2.09	1.28	
	European MMR-p/MSI-L+MSS	34	-3.21	2.86	
Sex	Female	63	-2.05	2.73	0.2353
	Male	55	-2.54	1.98	
COAD/READ Location	Left	35	-2.77	2.62	0.0678
	Right	83	-2.10	2.48	
COAD/READ Stage	1	31	-2.82	2.45	**0.0476**
	2	39	-2.05	2.29	
	3	39	-2.08	2.71	
	4	9	-2.75	3.85	

Bold values indicate significant p-values < 0.05.

**Table 5:** Multivariable regression model of estimated differences in *C1ORF43* Ct – *CXCL10* Ct results with 95% CI from an independent set enriched for MMR-d/MSI-H COAD/READ FFPE samples.

Effect	Level	Estimated differences (95% CI)	P-value*
Ancestry	African vs European	0.70 (0.02, 1.38)	**0.0438**
MMR/MSI Status	MMR-d/MSI-H vs. MMR-p/MSI-L+MSS	0.97 (0.22, 1.72)	**0.0119**
COAD/READ Location	Left vs Right	-0.45 (-1.26, 0.36)	0.2711
COAD/READ Stage	2 vs 1	0.43 (-0.46, 1.31)	0.5232
	3 vs 1	0.68 (-0.22, 1.57)	
	4 vs 1	0.36 (-1.02, 1.75)	

*P-values were calculated based on type 3 tests from a multiple linear regression model.

Bold values indicate significant p-values < 0.05.

**Table 6:** Multivariable regression model of estimated differences in *RAB7A* Ct – *CXCL10* Ct results with 95% CI from an independent set enriched for MMR-d/MSI-H COAD/READ FFPE samples.

Effect	Level	Estimated differences (95% CI)	P-value*
Ancestry	African vs European	0.67 (0.00, 1.35)	**0.0497**
MMR/MSI Status	MMR-d/MSI vs MMR-p/MSI-L+MSS	0.64 (-0.11, 1.38)	0.0923
COAD/READ Location	Left vs Right	-0.53 (-1.33, 0.27)	0.1951
COAD/READ Stage	2 vs 1	0.64 (-0.23, 1.51)	0.1725
	3 vs 1	0.87 (-0.02, 1.76)	
	4 vs 1	-0.11 (-1.48, 1.26)	

*P-values were calculated based on type 3 tests from a multiple linear regression model.

Bold values indicate significant p-values < 0.05.

**Figure 4:**
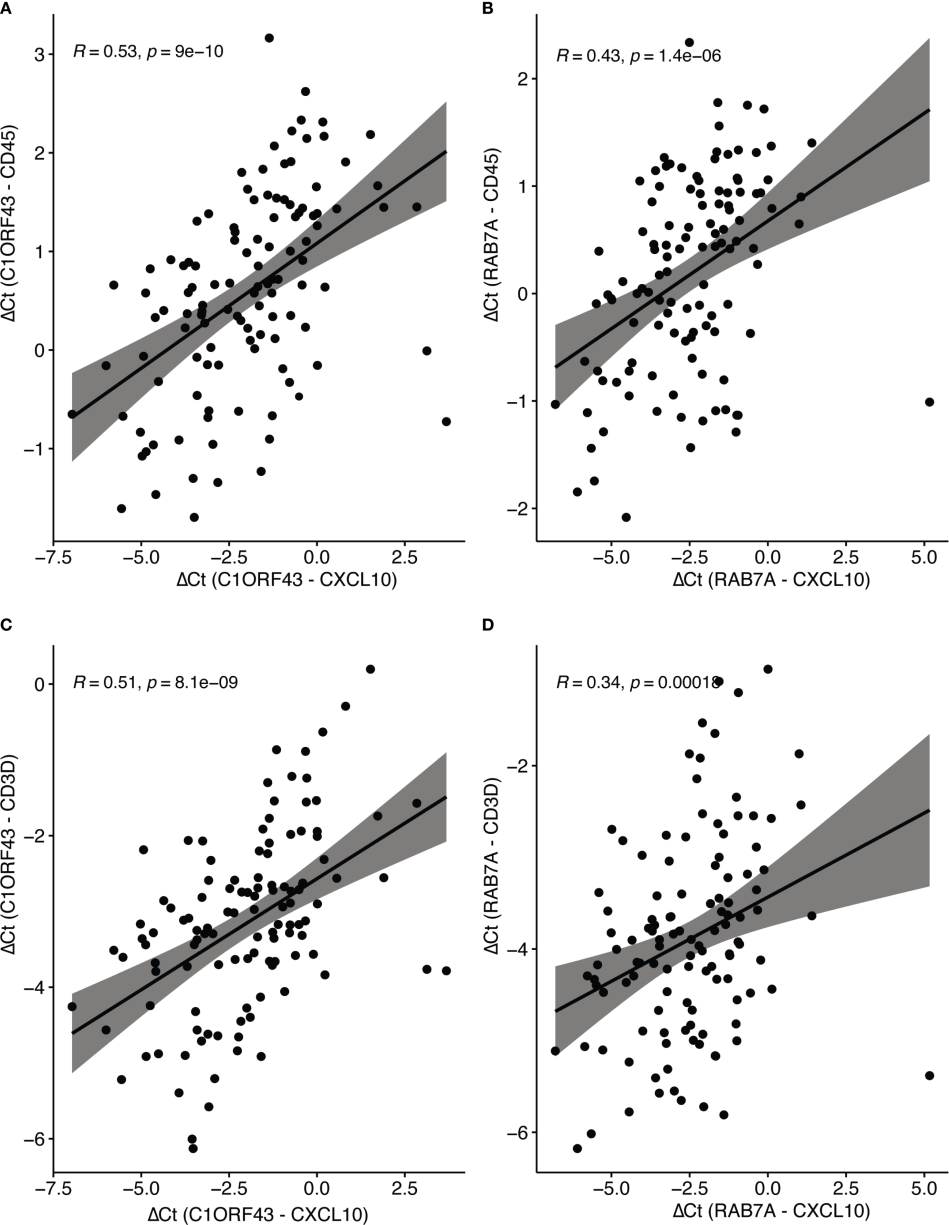
Spearman correlation of RT-qPCR CXCL10 and Immune Cell Type Markers (CD45 and CD3d). Scatter plots show correlations between ΔCt values of CXCL10 (normalized to reference genes C1ORF43 or RAB7A). **(A)** CXCL10 vs CD45 (C1ORF43 reference). **(B)** CXCL10 vs CD45 (RAB7A reference). **(C)** CXCL10 vs CD3D (C1ORF43 reference). **(D)** CXCL10 vs CD3D (RAB7A reference).

Parallel RT-qPCR assays were conducted for *CD45*, a myeloid/general white cell marker, and *CD3D*, a T-cell marker in the independent set of COAD/READ FFPE samples (see **Figure 3**). In single cell RNA-sequence datasets, *CXCL10* has been shown to be highly expressed in myeloid cell types (29). CXCL10 has been shown to be a T-cell attractant (36). Consistent with these reports are the strong positive correlations (see **Figure 4**) observed between *C1ORF43* Ct - *CXCL10* Ct and *C1ORF43* Ct - *CD45* Ct (Spearman correlation coefficient = 0.54, p-value < 0.0001), between *RAB7A* Ct - *CXCL10* Ct and *RAB7A* Ct - *CD45* Ct (Spearman correlation coefficient r =0.44, p-value < 0.0001), between *C1ORF43* Ct- *CXCL10* Ct and *C1ORF43* Ct – *CD3D* Ct (Spearman correlation coefficient r = 0.51, p-value < 0.0001), and between *RAB7A* Ct - *CXCL10* Ct and *RAB7A* Ct – *CD3D* Ct (Spearman correlation coefficient r = 0.34, p value < 0.0001).

## 5 Discussion

Previous analyses of the AA vs. EA TCGA-COAD dataset have highlighted AA vs. EA DEGs, particularly lower expression of immune related genes in the African vs. European ancestry groups. The current study differs from the previous studies by using multivariable analysis to examine potentially confounding variables such as MMR/MSI status, rather than attempting to control for these factors by propensity matching. Differences in the DEGs identified in the current study from previous studies may relate to the use of unprocessed counts as opposed to processed counts as input, and use of different DEG platforms (edgeR and Wilcoxon rank sum test). The current study identified five AA vs. EA immune related genes, including *CXCL10* that were expressed at lower levels in the AA, but expressed at higher level in MMR-d/MSI-H group. *CXCL10* is part of the CXCL9, 10, 11-CXCR3 axis, which plays an important role in tumor immune microenvironment remodeling (36). *CXCL10* has been positively correlated with COAD/READ survival (37, 38).

The percentages of MMR-d/MSI-H in the TCGA-COAD-READ AA vs. EA samples were 14.1% vs. 16.0%, consistent with previous reports that the prevalence of MSI-d/MSI-H is lower in the AA vs. EA cohort (18). This small difference in prevalence does not explain the greater than 2-fold difference in *CXCL10* expression between the two groups. Multivariable models confirmed that TCGA-COAD-READ *CXCL10* expression values were lower in AA vs. EA, and higher in MMR-d/MSI-H vs. MMR-p/MSI-Low, while controlling for COAD/READ location and COAD/READ stage. Neither COAD/READ location nor stage were significantly associated with TCGA *CXCL10* levels, when controlling for ancestry and MMR/MSI status. The total number of AA MMR-d/MSI-H samples was only 9 of 348 total samples, which clearly restricted the ability to detect statistically significant differences in *CXCL10* expression across groups stratified by both ancestry and MMR/MSI-status.

The consensus molecular subtype (CMS) classification is the most widely accepted gene expression based categorization of transcriptional profiles. It was based on applying machine learning (random forest) to five publicly accessible COAD/READ bulk RNA sequencing datasets, including the TCGA dataset (24). Our results demonstrate that the distribution of CMS1 labels in the AA cohort is very different from that of the EA cohort. Only in the EA cohort is the high association between CMS1 classification and MMR-d/MSI status observed. Furthermore, the significantly increased *CXCL10* expression values in the CMS1 group compared to CMS2 and CMS3 groups is observed only in the EA cohort and not in the AA cohort. If the public datasets used to develop the CMS classifications had poor representation of self-identified African ancestry COAD/READ samples, this could explain the different distributions of CMS labels between the two ancestry groups. Associations of the CMS3 classification and African ancestry and obesity has recently been reported (39, 40). Because obesity has been reported to be most prevalent in US self-identified African ancestry AA group (41), it may be important to control for obesity as a potentially confounding variable. Unfortunately, many of the TCGA-COAD-READ samples are missing body mass index (BMI) values.

The limited number of AA MMR-d/MSI-H samples in TCGA-COAD combined with the lack of AA samples annotated for MMR/MSI status from commercial vendors underscores the need for ancestrally diverse cohorts with robust clinical annotations. To further investigate the effect of African ancestry across MMR/MSI status, an independent set of COAD/READ FFPE samples was assembled from three medical centers that was enriched for AA and MMR-d/MSI-H samples. Approximately 2% of MMR-d/MSI COAD/READ have germline mutations or Lynch syndrome, but COAD/READ samples with germline MMR mutations were excluded from this independent set of samples. The relative proportion of the AA group was increased to match the number of EA group, and the percentage of MMR-d/MSI-H samples increased to 39% in the AA group and 42% in the EA group of the independent set of COAD FFPE samples. Normalized *CXCL10* mRNA expression (see **Tables 1**, **3**, **4**) was significantly higher in the MMR-d/MSI-H group vs. the MMR-p/MSI-L+MSS groups for both the TCGA-COAD-READ RNA-sequence data set (p-value=0.0003) and the independent FFPE RT-qPCR datasets (p-value =0.0003 for *C1ORF43* as reference gene, p-value = 0.0008 for *Rab7a* as the reference gene). However, there were differences between the TCGA-COAD-READ RNA-sequence dataset and the independent FFPE RT-qPCR results, when normalized *CXCL10* expression values were compared between African vs. European ancestry groups. While normalized *CXCL10* mRNA expression was significantly lower in the AA vs. EA TCGA-COAD-READ RNA-sequence data set, no significant difference was observed between the AA vs. EA independent set RT-qPCR datasets using either of the two reference genes (see **Tables 1**, **3**, **4**). In fact, the normalized RT-qPCR *CXCL0* mRNA values trended somewhat higher in the AA vs. EA group for both reference genes. The discordant AA vs. EA *CXCL10* results between the TCGA-COAD-READ and the independent FFPE datasets could potentially relate to 1.) differences in the proportion of AA MMR-d/MSI-H, AA MMR-p/MSI-L+MSS, EA MMR-d/MSI-H, EA MMR-p/MSI-L+MSS samples; 2.) MSI PCR-capillary electrophoresis (CE) based classification of MMR-d/MSI-H for the TCGA-COAD-READ vs. MMR-immunohistochemical (IHC) based classification of MMR-d; 3.) difference in the quality of the RNA extracted from frozen vs. FFPE tissues, 4.) differences in normalization for RNA-sequence vs. RT-qPCR measurements of mRNA expression, 5.) differences in correlations between self-identification of African ancestry and genomic estimates of African/European ancestry admixture. In the US the average genomic based estimate of African ancestry admixture is 73% in the self-identified African ancestry population (42), however the variation in African ancestry admixture compared to a reference Nigerian population can range from 30% to close to 100% (43). The sizes of the four ancestry MMR/MSI status groups were very unbalanced in the TCGA-COAD-READ group compared to the independent set of FFPE samples. With only 9 AA MMR-d/MSI-H samples in the TCGA-COAD-READ group, sampling bias particularly with respect to genomic estimates of African/European ancestry admixture could be the basis for discordant results between the TCGA-COAD-READ and the independent set of FFPE samples assembled from three medical centers. Because concordance between MMR-IHC and MSI PCR-CE has been reported to be 98% (44), it is unlikely that using two separate methods for classifying MMR-d/MSI-H explains the discordant results between the TCGA-COAD-READ and the independent FFPE set of tissues. The quality of the RNA recovered from FFPE samples is poor (RIN ~2) compared to frozen tissue (RIN >6) and typically exhibits higher Ct values in RT-qPCR assays compared to parallel frozen samples (45). It has been shown that while RT-qPCR and RNA-sequence results correlate, the correlation is surprisingly modest with r ~0.6 (45). For RNA-sequence data normalization is conducted using multiple genes. In contrast, RT-qPCR results are normalized against a single reference gene. For this reason, we selected Taqman primer probe sets for two reference genes that had been previously vetted for RT-qPCR analysis of COAD/READ RNA (27). To compare RNA-sequencing with RT-qPCR and to identify additional AA vs. EA DEGs, we are submitting this independent FFPE RNA sample set for parallel RNA-sequencing enriched by exome capture (46) and plan to continue to increase the size of the independent FFPE set of samples.

In summary, this study did not detect a significant ancestry effect on *CXCL10* expression across MMR status but confirmed that *CXCL10* mRNA expression is higher in MMR-d/MSI-H than MMR-p/MSI-L+MSS COAD/READ. Disentangling the effect of African ancestry from other co-variables such as MMR/MSI status requires increasing representation of minority samples across MMR/MSI status, genomic based estimation of ancestry admixture and rigorous collection of potential confounding metadata variables for all samples.

## Data Availability

The original contributions presented in the study are included in the article/**Supplementary Material**. Further inquiries can be directed to the corresponding author.

## Supplementary material

Supplementary Figure 1 Spearman correlation between *CXCL10* log_2_TPM values and CIBERSORT estimate of T-cell abundance in the TCGA-COAD-READ RNA-sequence dataset.

Supplementary Figure 2 Scatter plot of *CXCL10* log_2_TPM values in **(A)** AA CMS1, CMS2, CMS3, CMS4 and No Labels groups; and **(B)** EA CMS1, CMS2, CMS3, CMS4 and No Labels groups.
