# Supplementary material for: The effect of African ancestry and mismatch-repair enzyme deficiency/microsatellite instability-high on colorectal adenocarcinoma immune gene expression
Source: Front Gastroenterol (Lausanne). 2025 Oct 3;4:1638438. doi: 10.3389/fgstr.2025.1638438 (PMC12867126; doi:10.3389/fgstr.2025.1638438)
Supplement: Supplementary Figure 1 — Spearman correlation between CXCL10 log2TPM values and CIBERSORT estimate of T-cell abundance in the TCGA-COAD-READ RNA-sequence dataset. [file DataSheet1.pdf]

Supplementary Table S1A SUNY Stonybrook/Downstate edgeR AA vs. EA DEGs

SUNY Stonybrook/Downstate DEG	edgeR		African vs. European ancestry
Gene	logFold change	FDR	Direction
RNU1.28P	-4.051233265	0.017517	downregulated
RNU1.1	-4.051233265	0.017517	downregulated
RNVU1.18	-4.051233265	0.017517	downregulated
RNU1.3	-4.051233265	0.017517	downregulated
RNVU1.7	-3.846244891	0.045052	downregulated
LINC01610	-3.794311389	0.009564	downregulated
RNU1.27P	-3.662416417	0.025504	downregulated
RNU1.2	-3.662416417	0.025504	downregulated
RNU1.4	-3.662416417	0.025504	downregulated
RNVU1.29	-3.662416417	0.025504	downregulated
MYH7B	-3.534495807	0.02925	downregulated
CXCL11	-3.277964413	0.017283	downregulated
IGHV4.4	-3.116264997	0.028418	downregulated
CPS1	-3.112524874	0.044325	downregulated
**IDO1**	-2.938080128	0.020392	downregulated
RPPH1	-2.862305034	0.028925	downregulated
IGF2BP3	-2.825830135	0.039056	downregulated
**CXCL10**	-2.745880936	0.038444	downregulated
ERVMER34.1	-2.685311699	0.021143	downregulated
UCA1	-2.667532173	0.04107	downregulated
TBC1D3L	-2.635125146	0.036072	downregulated
ARSL	-2.591017081	0.025504	downregulated
ERP27	-2.556494826	0.038399	downregulated
KLRC2	-2.525042147	0.041818	downregulated
FBXO2	-2.375397882	0.04052	downregulated
PCDHGA10	-2.124246182	0.04262	downregulated
XKR9	-2.115533945	0.02023	downregulated
UBD	-2.088524203	0.037296	downregulated
LINC02577	-2.073183791	0.009564	downregulated
GZMB	-2.029553655	0.015162	downregulated
SENP3.EIF4A1	-2.010786008	0.044742	downregulated
STPG4	-1.98076857	0.02406	downregulated
WDR97	-1.971039518	0.025504	downregulated
ELFN1.AS1	-1.890252121	0.026852	downregulated
MYOM3	-1.852961216	0.032732	downregulated
SMKR1	-1.747823937	0.035947	downregulated
ESYT3	-1.702462992	0.037601	downregulated
DDN	-1.686477036	0.031131	downregulated
RAB17.DT	-1.681818167	0.017283	downregulated
AMBP	-1.638804432	0.043551	downregulated
BEGAIN	-1.635304876	0.020967	downregulated
ADGRF4	-1.632652618	0.045494	downregulated
PKDREJ	-1.483573486	0.030622	downregulated
NPM2	-1.457158023	0.04107	downregulated

LINC01106	-1.420169856	0.001992	downregulated
PSAT1	-1.412235598	0.009564	downregulated
FAM72D	-1.372676575	0.009564	downregulated
PAX6	-1.281677131	0.039159	downregulated
EIF5AL1	-1.234147841	0.048289	downregulated
NEIL3	-1.227540793	0.002048	downregulated
RDM1	-1.212639269	0.023284	downregulated
ERCC6L	-1.157101621	0.028037	downregulated
UBAC2.AS1	-1.149136844	0.037685	downregulated
CFAP263	-1.128023872	0.025421	downregulated
NUP62CL	-1.113457706	0.009564	downregulated
RPL17.C18orf32	-1.113075958	0.047491	downregulated
CDCA2	-1.106379785	0.045612	downregulated
SPATA31H1	-1.098551534	0.012529	downregulated
CLSPN	-1.09234242	0.036126	downregulated
EME1	-1.084578997	0.014339	downregulated
CFAP157	-1.082597432	0.026852	downregulated
RGPD8	-1.074507182	0.035947	downregulated
ESCO2	-1.073304218	0.045494	downregulated
HASPIN	-1.06821699	0.023075	downregulated
LINC01569	-1.060641463	0.019677	downregulated
CENPU	-1.054551185	0.009564	downregulated
SPIN4	-1.050589814	0.021135	downregulated
ADGRG5	-1.05038219	0.034529	downregulated
STRIP2	-1.041972062	0.036072	downregulated
TNNI1	-1.030444258	0.049546	downregulated
MAP3K9.DT	-1.028699386	0.026444	downregulated
WARS1	-1.017346607	0.045612	downregulated
SORD	-1.009988375	0.032023	downregulated
POLR3G	-1.001205902	0.047491	downregulated
PSMB9	-1.000493116	0.028962	downregulated
PLXDC2	1.006255973	0.048799	upregulated
PINLYP	1.011315474	0.040432	upregulated
MEF2C	1.018485988	0.025504	upregulated
SMG1P4	1.020235359	0.031542	upregulated
ZNF366	1.026136304	0.038399	upregulated
KLF9	1.028556429	0.04005	upregulated
C1QTNF2	1.040063933	0.048783	upregulated
TNS2	1.040110908	0.01583	upregulated
NOTCH4	1.042039127	0.047378	upregulated
PLPP3	1.0556337	0.004141	upregulated
SVEP1	1.056400532	0.039159	upregulated
CCDC3	1.056811829	0.04259	upregulated
GASK1B	1.062512264	0.042555	upregulated
HEG1	1.06456976	0.029662	upregulated
PREX2	1.06522177	0.033215	upregulated
KLF8	1.067043113	0.025421	upregulated

GIMAP1	1.071327528	0.030836	upregulated
SEPTIN4	1.079191554	0.04331	upregulated
ITGA9	1.088484039	0.032732	upregulated
ADGRB1	1.088929362	0.045612	upregulated
KDR	1.09004373	0.02925	upregulated
ROBO4	1.091229646	0.043551	upregulated
INKA1	1.092425882	0.049249	upregulated
SLC8A1.AS1	1.09294621	0.034506	upregulated
NRIP2	1.095899703	0.009564	upregulated
PRSS23	1.11269053	0.0497	upregulated
RASGRP2	1.115094634	0.038399	upregulated
CHST7	1.126122609	0.017945	upregulated
PER1	1.128486226	0.019701	upregulated
CDH6	1.128526373	0.023138	upregulated
TEF	1.130025414	0.016799	upregulated
GPRASP1	1.138712652	0.03919	upregulated
TM4SF18	1.139601516	0.022419	upregulated
TBX2	1.141345679	0.048378	upregulated
CACNA2D2	1.144048399	0.040691	upregulated
ZNF542P	1.150111876	0.0382	upregulated
TMEM204	1.156823127	0.042941	upregulated
FBXL7	1.168362991	0.037686	upregulated
SDC2	1.168408116	0.041956	upregulated
IGFBP7	1.169887206	0.04914	upregulated
MPDZ	1.173107863	0.045425	upregulated
DAAM2	1.174031286	0.022419	upregulated
CAMK1D	1.174130098	0.033117	upregulated
FZD8	1.178088012	0.026444	upregulated
ZNF135	1.181552948	0.044742	upregulated
EXOC3L1	1.182818663	0.012902	upregulated
SETBP1	1.185592893	0.038399	upregulated
NDN	1.1871641	0.037353	upregulated
SOX18	1.189573829	0.048289	upregulated
HRC	1.19328553	0.021937	upregulated
EMILIN3	1.196672095	0.0448	upregulated
CYYR1	1.202216475	0.02023	upregulated
MIR497HG	1.207280742	0.04107	upregulated
TAMALIN	1.208771715	0.025504	upregulated
SH2D3C	1.212689679	0.022419	upregulated
S1PR1	1.21505502	0.018827	upregulated
CALCRL	1.22480899	0.019677	upregulated
RASL12	1.228664767	0.044325	upregulated
FAM124B	1.234089877	0.046453	upregulated
THBD	1.234268473	0.0382	upregulated
SOX7	1.235218438	0.020723	upregulated
LDB2	1.235831885	0.016799	upregulated
DIPK2B	1.235913395	0.026852	upregulated

GNG2	1.235950078	0.02406	upregulated
HSPA12B	1.236705049	0.031776	upregulated
SAMD11	1.238756177	0.038399	upregulated
TMEM88	1.24043995	0.02023	upregulated
MEG3	1.241961427	0.039159	upregulated
KLF2	1.244080679	0.037685	upregulated
CCM2L	1.247625101	0.017283	upregulated
SEMA6C	1.248497295	0.008534	upregulated
CDH5	1.2492146	0.02406	upregulated
GLI1	1.251334891	0.025504	upregulated
CD34	1.253512365	0.013499	upregulated
PTCH2	1.2566435	0.046009	upregulated
NHSL2	1.256799001	0.040316	upregulated
CCL14	1.25695328	0.039085	upregulated
SCHIP1	1.256969272	0.049721	upregulated
ESAM	1.257498127	0.021143	upregulated
LRRC32	1.258371979	0.017899	upregulated
KIF5C	1.259883888	0.024193	upregulated
MTND2P28	1.260630436	0.04262	upregulated
C2orf74	1.265319171	0.037685	upregulated
ARHGEF15	1.267669123	0.023075	upregulated
SGK1	1.269237299	0.038399	upregulated
HLX	1.272501787	0.036072	upregulated
PLA2G4C	1.275806799	0.01409	upregulated
HIC1	1.276904078	0.014339	upregulated
A2M	1.277838692	0.01153	upregulated
ECSCR	1.278801945	0.031542	upregulated
RAB44	1.280918695	0.040432	upregulated
SERPINF1	1.281602478	0.04005	upregulated
ZNF667	1.281979145	0.038804	upregulated
PALMD	1.283575284	0.041956	upregulated
ACSS3	1.285167083	0.044325	upregulated
COPZ2	1.285426111	0.046184	upregulated
OLFML2A	1.291371446	0.031482	upregulated
EGFL7	1.291824319	0.017517	upregulated
MMRN2	1.294492128	0.016799	upregulated
TIE1	1.302274386	0.035692	upregulated
TRO	1.30247656	0.048348	upregulated
MAPK10	1.303571752	0.040076	upregulated
CCDC8	1.304754732	0.047378	upregulated
EDNRB	1.307853474	0.037685	upregulated
CRISPLD1	1.309416931	0.047491	upregulated
TEK	1.309985367	0.01153	upregulated
RAMP3	1.313726421	0.025504	upregulated
ESPNL	1.315637541	0.039159	upregulated
FLRT2	1.319755509	0.039333	upregulated
FHL5	1.322381263	0.018827	upregulated

GPX3	1.323088231	0.047491	upregulated
GPR162	1.327387939	0.026852	upregulated
RAB7B	1.328403493	0.02023	upregulated
C1QTNF3	1.330757692	0.023931	upregulated
C1QTNF5	1.332829262	0.03967	upregulated
COLEC11	1.336711946	0.023075	upregulated
ADAMTS10	1.339059142	0.040076	upregulated
EBF1	1.342281857	0.039361	upregulated
ZNF667.AS1	1.343323087	0.031114	upregulated
CAND2	1.34496106	0.031807	upregulated
DEPP1	1.350277827	0.045607	upregulated
TSPAN18	1.350806397	0.021731	upregulated
COL14A1	1.351725956	0.02023	upregulated
INSYN1	1.352144023	0.04005	upregulated
TMEM119	1.352895742	0.04005	upregulated
FAM162B	1.357433891	0.029881	upregulated
PNPLA7	1.365477345	0.02023	upregulated
APLNR	1.369045996	0.026852	upregulated
KCNT2	1.371042223	0.0448	upregulated
MIR3667HG	1.372766729	0.038399	upregulated
ADHFE1	1.376086365	0.0448	upregulated
COL21A1	1.379429578	0.040432	upregulated
SCARA3	1.387477017	0.031542	upregulated
RAMP2	1.394414579	0.019677	upregulated
PDGFD	1.398473952	0.021143	upregulated
LIMS2	1.403066495	0.039159	upregulated
A1BG	1.410290172	0.008376	upregulated
PDE1A	1.411247259	0.025504	upregulated
CLEC14A	1.417148447	0.017283	upregulated
RBP7	1.423959593	0.04262	upregulated
DOK6	1.425215869	0.030814	upregulated
SHE	1.428702767	0.009564	upregulated
ECM2	1.428875593	0.049287	upregulated
PDE7B	1.440098963	0.0382	upregulated
ADAMTS1	1.441136787	0.025504	upregulated
SEMA3G	1.446553229	0.010166	upregulated
FIGN	1.447921204	0.039253	upregulated
COX4I2	1.448098142	0.047491	upregulated
THSD7A	1.448627612	0.017945	upregulated
NMUR1	1.449997903	0.009564	upregulated
SLC26A10P	1.455598401	0.047185	upregulated
GASK1A	1.459081108	0.04107	upregulated
AFF3	1.459842821	0.032744	upregulated
SLC2A4	1.459985324	0.048289	upregulated
SLC15A2	1.461334124	0.037685	upregulated
THY1	1.462475718	0.046209	upregulated
DMD	1.46569829	0.047491	upregulated

SOX17	1.472740717	0.023075	upregulated
PEG3	1.472779815	0.044675	upregulated
NPR1	1.481690094	0.009564	upregulated
MAP2	1.491068861	0.020106	upregulated
NAP1L3	1.493640557	0.034792	upregulated
ADGRD1	1.502205157	0.0382	upregulated
USHBP1	1.505656443	0.02023	upregulated
PPP1R3C	1.520275803	0.04191	upregulated
ADAMTSL1	1.521552718	0.022419	upregulated
ADCY2	1.522016386	0.028418	upregulated
MT.TA	1.522811099	0.04052	upregulated
KANK3	1.526492959	0.008534	upregulated
CACNA1H	1.533218232	0.025575	upregulated
ADAMTS8	1.541582464	0.03919	upregulated
NEGR1	1.542616289	0.048289	upregulated
AR	1.54512878	0.01468	upregulated
PIEZO2	1.560459915	0.009564	upregulated
CYS1	1.56249408	0.048556	upregulated
ZNF418	1.565407981	0.017763	upregulated
GJA4	1.565976713	0.020106	upregulated
CLEC3B	1.567277085	0.043525	upregulated
FBLN1	1.575602206	0.016139	upregulated
CD1C	1.57594625	0.017283	upregulated
GADD45B	1.583222003	0.024193	upregulated
NUDT11	1.583941312	0.039333	upregulated
VWFP1	1.585922395	0.019677	upregulated
PODN	1.589483458	0.017283	upregulated
LIFR	1.604522767	0.033144	upregulated
SEMA5B	1.619432475	0.015145	upregulated
WHAMMP2	1.626831032	0.021861	upregulated
GFRA2	1.630903169	0.048048	upregulated
CH25H	1.638339759	0.026852	upregulated
HSPB2.C11orf52	1.643962343	0.041956	upregulated
JAM2	1.646218165	0.023075	upregulated
ADAM33	1.650748985	0.0448	upregulated
LPL	1.651196383	0.026852	upregulated
PLD4	1.65242684	0.02023	upregulated
GHR	1.653881436	0.04005	upregulated
SCARF1	1.656275184	0.017283	upregulated
CARMN	1.663251371	0.019677	upregulated
TLL1	1.663352562	0.04262	upregulated
MPZ	1.663413097	0.02023	upregulated
CSRNP3	1.671107222	0.030028	upregulated
FAM107A	1.676465826	0.017517	upregulated
RASD1	1.682863936	0.048289	upregulated
DACT3	1.683042886	0.0451	upregulated
PDE1B	1.685078221	0.024049	upregulated

MYL9	1.685219548	0.046184	upregulated
CXCL12	1.68621522	0.017283	upregulated
CDH23	1.69316734	0.008534	upregulated
FBXL22	1.715221181	0.02084	upregulated
EMCN	1.724914603	0.002048	upregulated
PLAC9	1.725999198	0.012529	upregulated
GPR146	1.72632865	0.012529	upregulated
TRIL	1.734394545	0.002048	upregulated
VMO1	1.736881204	0.032732	upregulated
PDE2A	1.746576172	0.00218	upregulated
TMEM255A	1.75576148	0.02406	upregulated
LINC00528	1.761558434	0.048289	upregulated
ATP1B2	1.770133541	0.008534	upregulated
RGMA	1.770643106	0.034792	upregulated
TENT5B	1.774730555	0.028287	upregulated
TNXB	1.777612332	0.017899	upregulated
AQP1	1.777818125	0.004141	upregulated
LINC01140	1.78762511	0.026444	upregulated
C1QTNF7	1.820395396	0.01377	upregulated
GCAWKR	1.829094526	0.017283	upregulated
FAM13C	1.830155108	0.001992	upregulated
FCER1A	1.839505304	0.02084	upregulated
TRGC1	1.851758737	0.0448	upregulated
CD300LB	1.854785384	0.008534	upregulated
SSTR2	1.87388609	0.020106	upregulated
SLC35F1	1.880643544	0.009564	upregulated
SOCS3	1.887788312	0.0497	upregulated
NOVA1	1.888440407	0.0448	upregulated
GNAO1	1.901262058	0.036586	upregulated
SCN9A	1.905210162	0.048654	upregulated
PPP1R14A	1.907215445	0.008534	upregulated
CFD	1.919698267	0.009564	upregulated
ZNF626	1.927111981	0.01153	upregulated
SLIT3	1.944999372	0.008376	upregulated
PKNOX2	1.949989448	0.021207	upregulated
CAVIN2	1.953347903	0.007802	upregulated
STEAP4	1.960975913	0.020231	upregulated
GPC3	1.973840193	0.008534	upregulated
FXYD1	1.974966579	0.02023	upregulated
SCN4B	1.99431132	0.004992	upregulated
SCUBE2	2.004143568	0.007262	upregulated
LINC02568	2.005634962	0.04005	upregulated
LGI4	2.016175515	0.004084	upregulated
IGF1	2.029131336	0.023139	upregulated
GALNT16	2.05201958	0.012694	upregulated
PTX3	2.066738521	0.047491	upregulated
PWP2	2.067435951	0.007262	upregulated

NBEA	2.136671955	0.025504	upregulated
EBF2	2.190209789	0.036126	upregulated
GSTM5	2.192386163	0.002048	upregulated
PYGM	2.240674092	0.007275	upregulated
SLCO4C1	2.278564188	0.014221	upregulated
TAGAP	2.284984151	0.040316	upregulated
CASS4	2.287446123	0.04262	upregulated
FLT3	2.296482762	0.032942	upregulated
MT1M	2.329570869	0.045612	upregulated
RETNLB	2.353964339	0.043756	upregulated
CCL4L2	2.373094573	0.048289	upregulated
GATD3	2.379702177	0.002048	upregulated
DPT	2.387840551	0.022012	upregulated
GSTM1	2.394146004	0.041428	upregulated
CD69	2.404587023	0.040129	upregulated
CD36	2.421734058	0.010083	upregulated
LDB3	2.440721666	0.037824	upregulated
HSPB6	2.445012011	0.041082	upregulated
MFAP5	2.52304404	0.019677	upregulated
CHRDL1	2.552760686	0.026852	upregulated
EPHA7	2.584639659	0.037685	upregulated
CNTN1	2.599567173	0.012529	upregulated
EEF1A1P11	2.620945108	0.010984	upregulated
GRIK3	2.639811261	0.003256	upregulated
IL1RN	2.809769685	0.047491	upregulated
FFAR2	2.829968439	0.031542	upregulated
MAMDC2	2.835126369	0.017107	upregulated
GREM2	2.875248796	0.017517	upregulated
ABCA8	2.877319196	0.023075	upregulated
PLIN4	2.882315196	0.026852	upregulated
HAND2.AS1	3.025911789	0.021344	upregulated
TACR2	3.03623956	0.017899	upregulated
AQP8	3.246854561	0.034506	upregulated
C7	3.248226404	0.009564	upregulated
SFRP1	3.277108963	0.008534	upregulated
CCL3L3	3.282966368	0.032942	upregulated
EEF1A1P13	3.312152408	0.008534	upregulated
TFF2	3.377022535	0.034792	upregulated
ACTL8	3.505766918	0.024028	upregulated
PRIMA1	3.52528277	0.009022	upregulated
OGN	3.645575443	0.008534	upregulated
UGT1A1	3.741992049	0.017517	upregulated
ADH1B	3.864729567	0.009564	upregulated
ATP1A2	4.413654174	0.002372	upregulated
MTCO1P12	4.817103312	0.000437	upregulated
FABP4	4.94431422	0.008376	upregulated
MTND1P23	9.914041363	1.36E-08	upregulated

Supplementary Table S1B TCGA edgeR MMR-d/MSI-H vs. MMR-p/MSI-L+MSS DEGs

TCGA DEG	edgeR		MMR-d/MSI-H vs. MMR-p/MSI-L+MSS
Gene	log Fold change	FDR	Direction
CALCB	-6.706730854	2.50625E-12	downregulated
PRSS56	-6.19940234	2.78303E-12	downregulated
PPBP	-5.73215565	3.32777E-09	downregulated
COL2A1	-5.504232759	1.27368E-14	downregulated
MAGEA4	-4.606869623	2.66654E-09	downregulated
ITLN2	-4.490352421	1.71348E-09	downregulated
NEUROD1	-4.319831963	7.85577E-10	downregulated
SLC30A2	-4.189748409	5.7204E-19	downregulated
KRT40	-4.180168421	1.89728E-09	downregulated
FGF20	-4.141019993	1.86633E-12	downregulated
SERPINA7	-4.021103932	3.69282E-15	downregulated
GDF10	-4.020362614	9.69284E-11	downregulated
INSL5	-4.016679071	4.01504E-09	downregulated
WIF1	-4.002880636	1.05388E-09	downregulated
HSPB3	-3.881819361	4.40204E-11	downregulated
SLC14A1	-3.845594484	8.59341E-11	downregulated
TRIM71	-3.836747798	8.65254E-11	downregulated
MS4A12	-3.768889311	2.80423E-10	downregulated
ZNF663P	-3.761508443	2.61795E-09	downregulated
MAGEA6.DT	-3.731711057	7.41902E-08	downregulated
VWA5B1	-3.704739743	2.57874E-11	downregulated
KRT31	-3.69741894	3.83661E-07	downregulated
FADS6	-3.64713472	3.39951E-12	downregulated
F7	-3.633858286	2.11418E-17	downregulated
SULT2A1	-3.575412906	1.08154E-11	downregulated
SV2C.AS1	-3.569518703	1.82622E-07	downregulated
POU6F2.AS1	-3.511882959	6.66578E-08	downregulated
IGF2	-3.503210554	1.27991E-06	downregulated
CLDN8	-3.497686216	3.49123E-08	downregulated
IGF2BP1	-3.489280222	1.67355E-08	downregulated
TMIGD1	-3.474280664	1.31551E-09	downregulated
NOTUM	-3.463006156	9.24178E-10	downregulated
SLC5A8	-3.458109969	1.96672E-07	downregulated
LY6G6D	-3.430350343	4.95678E-13	downregulated
LCN15	-3.422376936	3.74449E-06	downregulated
PTF1A	-3.418048467	3.47658E-08	downregulated
TMEM132C	-3.412584568	1.3013E-08	downregulated
FGF3	-3.406016856	1.39593E-07	downregulated
SLC22A11	-3.401243144	3.58982E-17	downregulated
KRT32	-3.395201489	4.85152E-07	downregulated
UGT2A3	-3.383080315	5.21431E-13	downregulated
NEUROG2	-3.37923814	1.62207E-10	downregulated
NTS	-3.345739131	1.48099E-07	downregulated
CELP	-3.335913984	5.9777E-19	downregulated
CA4	-3.335414321	4.94661E-09	downregulated
PHYHIPL	-3.317437132	4.58602E-11	downregulated
TUBAP13	-3.298629754	7.60073E-12	downregulated

CA1	-3.294400822	1.84141E-07	downregulated
SLC26A3	-3.293300699	6.37117E-11	downregulated
LINC02563	-3.290883954	4.01477E-12	downregulated
BHLHA9	-3.259780824	1.919E-08	downregulated
CACNG4	-3.256180853	1.44973E-11	downregulated
SCNN1B	-3.232704565	4.11549E-13	downregulated
ORM1	-3.226888129	1.49908E-06	downregulated
CPN1	-3.216468119	2.96671E-11	downregulated
IGF2.AS	-3.21547691	1.02129E-08	downregulated
RSPO4	-3.199555503	1.32656E-09	downregulated
MMP20.AS1	-3.191267897	1.66867E-10	downregulated
SVOPL	-3.185646085	4.04839E-11	downregulated
RNF182	-3.109943509	1.20227E-10	downregulated
KRT39	-3.092418823	1.35801E-08	downregulated
GUCA2B	-3.075039724	4.45879E-09	downregulated
ELAVL2	-3.061606202	1.85969E-14	downregulated
GNG4	-3.014518557	1.75656E-15	downregulated
LY6G6F.LY6G6	-2.997724314	8.5988E-11	downregulated
MUCL1	-2.9714883	2.03752E-08	downregulated
SPMIP5	-2.931881418	1.16351E-13	downregulated
AQP8	-2.92932068	1.19417E-07	downregulated
LINC00355	-2.920373555	1.56572E-06	downregulated
PCSK1N	-2.919926325	3.03188E-08	downregulated
SLC39A2	-2.919443166	1.22506E-08	downregulated
GPR83	-2.915617281	2.08427E-07	downregulated
TNNC2	-2.908404278	4.60013E-15	downregulated
KRT23	-2.865768645	7.93047E-11	downregulated
MYBPHL	-2.852949406	7.19928E-09	downregulated
APOA2	-2.849303756	3.76383E-06	downregulated
MMP20	-2.802955847	1.65635E-10	downregulated
LINC01923	-2.787877603	1.9665E-06	downregulated
UPK2	-2.779482867	3.79948E-08	downregulated
SLC38A3	-2.770500079	1.34072E-10	downregulated
C6orf15	-2.756157342	5.62186E-07	downregulated
TMEM252	-2.751260431	3.72398E-10	downregulated
AIRE	-2.747042683	3.30398E-10	downregulated
MAGEB17	-2.722667887	1.78184E-07	downregulated
PRR9	-2.717740577	2.31064E-06	downregulated
CTXND1	-2.716151509	4.66969E-08	downregulated
EYA1	-2.690945178	9.04513E-08	downregulated
CHGB	-2.690396287	5.34961E-06	downregulated
DRD2	-2.687798656	3.3399E-10	downregulated
AMER3	-2.670941395	2.27224E-07	downregulated
ZG16	-2.661841507	2.35231E-05	downregulated
GUCA2A	-2.656801027	6.89451E-10	downregulated
LRP2	-2.638981153	6.8954E-07	downregulated
NPTX2	-2.587885046	9.21899E-08	downregulated
VENTX	-2.584069468	1.20916E-08	downregulated
GRM8	-2.571908629	2.71438E-14	downregulated
DLX3	-2.571067446	2.12206E-08	downregulated

PCDH19	-2.556344881	2.90071E-09	downregulated
PKLR	-2.541342368	4.49754E-10	downregulated
POU5F1B	-2.535585419	1.86148E-19	downregulated
PCP4	-2.533607874	2.53411E-08	downregulated
UMODL1	-2.516173911	3.19068E-14	downregulated
RIIAD1	-2.495221457	1.17243E-10	downregulated
QPRT	-2.48753133	3.61427E-20	downregulated
UGT2B10	-2.48653466	4.52908E-07	downregulated
ISM2	-2.485299929	4.19947E-08	downregulated
PRAP1	-2.484028452	3.78976E-14	downregulated
GLYATL2	-2.479411768	7.29389E-09	downregulated
TAC1	-2.478624327	1.54707E-08	downregulated
REN	-2.477540634	2.49108E-08	downregulated
TCHH	-2.476597336	1.16003E-07	downregulated
MAP7D2	-2.471200876	4.19078E-09	downregulated
KNG1	-2.457870882	4.84597E-11	downregulated
ENPP3	-2.444259143	2.59159E-10	downregulated
LINC01411	-2.42885274	1.077E-06	downregulated
POU6F2	-2.420115394	2.14027E-06	downregulated
CERNA2	-2.413494371	1.14772E-09	downregulated
MAGEB17.AS1	-2.412092603	1.6591E-06	downregulated
LPO	-2.40455276	6.87168E-08	downregulated
MRLN	-2.383037439	7.30902E-06	downregulated
CYP4F8	-2.376586401	1.01434E-07	downregulated
CLCA4	-2.376531193	0.000121905	downregulated
SERPINA6	-2.375182016	2.49971E-08	downregulated
SYN3	-2.368404049	1.22575E-09	downregulated
SLC15A1	-2.366161148	1.13524E-09	downregulated
GABRA3	-2.365161047	1.85885E-05	downregulated
VSTM2L	-2.359071366	1.48386E-08	downregulated
APOB	-2.356181695	3.42009E-06	downregulated
KLHL34	-2.335085844	1.55478E-09	downregulated
CYP2B6	-2.334054518	9.53252E-15	downregulated
BOLA3P3	-2.331486695	7.20523E-15	downregulated
UPK1A	-2.324700566	1.67158E-06	downregulated
TCAP	-2.324125682	6.54302E-07	downregulated
DKK4	-2.316763409	2.22077E-05	downregulated
FOLR1	-2.29952228	1.33264E-06	downregulated
SYT4	-2.298589254	1.47876E-05	downregulated
SLC6A4	-2.295603945	1.38259E-09	downregulated
DIO3OS	-2.293861155	2.99828E-14	downregulated
STMND1	-2.281305298	5.46875E-11	downregulated
UTS2R	-2.27632964	3.66578E-06	downregulated
FAM240A	-2.268617215	1.36066E-09	downregulated
URAD	-2.265273627	8.58244E-07	downregulated
LINC02940	-2.264930985	4.5446E-11	downregulated
NKD1	-2.263688645	2.33781E-12	downregulated
HCAR1	-2.257125986	4.6613E-08	downregulated
RBP2	-2.256146331	2.30805E-08	downregulated
CXCL14	-2.255584437	1.12801E-12	downregulated

MEP1AP4	-2.254823922	1.72341E-10	downregulated
P2RX3	-2.254635222	1.13416E-08	downregulated
LINC01213	-2.25041941	8.85833E-10	downregulated
TMEM132D.A:	-2.248286468	0.000333245	downregulated
LINC02441	-2.245743861	8.7761E-17	downregulated
SEC14L4	-2.243422352	1.18282E-07	downregulated
TERLR1	-2.235855429	3.78957E-06	downregulated
LINC02251	-2.235517323	1.33043E-10	downregulated
VGLL1	-2.235187191	8.70173E-06	downregulated
RPL39P40	-2.231491555	1.97881E-14	downregulated
LHFPL7	-2.22984399	3.02501E-09	downregulated
GBX2	-2.225201927	8.62698E-09	downregulated
SLC13A2	-2.220413563	9.27352E-08	downregulated
PYY	-2.215572786	2.24597E-06	downregulated
C3orf85	-2.21016312	1.91028E-13	downregulated
LINC02525	-2.206757827	1.7356E-05	downregulated
SLC39A5	-2.205846907	5.34794E-20	downregulated
PTPRD.AS1	-2.198588897	1.13042E-14	downregulated
ELF5	-2.19043198	2.80744E-05	downregulated
TEKT5	-2.185572327	6.69894E-11	downregulated
BRINP2	-2.176780103	4.83528E-07	downregulated
GPR143	-2.174555378	6.19284E-14	downregulated
FABP1	-2.163021352	3.73999E-08	downregulated
DPP10.AS1	-2.157973391	8.98076E-06	downregulated
UGT1A8	-2.140621088	1.11101E-09	downregulated
FZD10	-2.139179372	3.64825E-05	downregulated
IGHA2	-2.138585769	3.29149E-05	downregulated
CAB39L	-2.136002852	5.84013E-19	downregulated
CRIPTO	-2.135628177	4.05412E-16	downregulated
APCDD1	-2.127963695	9.06983E-10	downregulated
GCG	-2.124444298	4.73216E-05	downregulated
OTOP2	-2.120300385	3.47078E-05	downregulated
ZNF285	-2.113634687	1.04835E-12	downregulated
ACRP1	-2.109953396	8.13041E-19	downregulated
STRA6	-2.107240845	3.38756E-10	downregulated
CTTNBP2	-2.105479502	4.69235E-17	downregulated
PIPOX	-2.100535572	3.86973E-09	downregulated
LINC00658	-2.100121815	2.00848E-06	downregulated
PTPRO	-2.099754149	1.24421E-10	downregulated
F10	-2.089818173	4.55944E-11	downregulated
COL9A1	-2.089679818	1.49956E-10	downregulated
HOXC12	-2.088976767	0.000221384	downregulated
TRPM6	-2.087210769	1.02132E-11	downregulated
CAMKV	-2.081725838	1.41048E-06	downregulated
AP3B2	-2.077239801	5.76347E-06	downregulated
CHP2	-2.074816406	1.55034E-08	downregulated
UPK3A	-2.072530113	4.6613E-08	downregulated
SLC1A7	-2.062315926	1.16437E-08	downregulated
COL9A3	-2.059382814	2.62796E-08	downregulated
LINC02128	-2.057181362	3.14676E-06	downregulated

NAT16	-2.04586459	2.74753E-07	downregulated
C19orf18	-2.037117531	1.84504E-11	downregulated
LRFN2	-2.034532138	6.62879E-07	downregulated
HUNK	-2.031944891	1.02315E-15	downregulated
SHISA9	-2.031878899	8.21728E-05	downregulated
LINC02805	-2.025796066	4.42908E-09	downregulated
ACSL6	-2.025050379	3.59789E-08	downregulated
POU6F2.AS2	-2.01739107	2.13478E-05	downregulated
SLC30A10	-2.015079375	2.55531E-06	downregulated
XPNPEP2	-2.010552163	2.75098E-07	downregulated
SCN5A	-2.005797868	9.72407E-10	downregulated
ASCL2	-2.00228439	1.09917E-15	downregulated
SLC26A2	-1.996139268	1.73137E-09	downregulated
GPR15LG	-1.996135316	1.2364E-09	downregulated
CPLX2	-1.995732815	2.75544E-06	downregulated
PCSK2	-1.995441017	3.08308E-05	downregulated
HMGCS2	-1.990915326	1.64234E-07	downregulated
GOLGA6L2	-1.987846281	1.00811E-05	downregulated
PHACTR3	-1.98752569	1.13226E-09	downregulated
SPMIP10	-1.977644098	2.4396E-11	downregulated
MTTP	-1.977199256	0.000305803	downregulated
MAGEA3	-1.976586608	0.003791723	downregulated
STOML3	-1.974788882	1.21167E-09	downregulated
KDM3AP1	-1.972335999	5.88149E-07	downregulated
PRORY	-1.970416451	2.89171E-05	downregulated
TRAV30	-1.969272089	5.2957E-08	downregulated
ZNF492	-1.96836376	3.09465E-06	downregulated
MEP1A	-1.967870515	4.95678E-11	downregulated
SLC13A3	-1.956737069	6.65016E-07	downregulated
ERVH.1	-1.945040632	4.87328E-06	downregulated
TUSC8	-1.939721121	1.47492E-12	downregulated
ACE2	-1.938972896	1.01232E-09	downregulated
TCP10L3	-1.935291118	1.5061E-11	downregulated
IGLJ2	-1.931853576	0.002696168	downregulated
MUC12	-1.926504421	9.8012E-08	downregulated
AXIN2	-1.921653961	2.34804E-19	downregulated
CHMP1B2P	-1.912815652	1.81054E-06	downregulated
ERP27	-1.90355555	4.35548E-08	downregulated
TARDBPP2	-1.902456063	4.81405E-08	downregulated
SCG3	-1.901796315	3.63788E-05	downregulated
RUBCNL	-1.899176797	7.75686E-14	downregulated
CRIPTO3	-1.898890904	4.73746E-07	downregulated
LINC01738	-1.898305531	1.71767E-10	downregulated
CST4	-1.896856446	3.3464E-07	downregulated
LINC01622	-1.894967733	3.62166E-12	downregulated
CPA6	-1.887390174	2.09579E-06	downregulated
MTCO3P12	-1.88625607	2.75621E-05	downregulated
PLA2G12B	-1.881970196	1.90558E-06	downregulated
CLDN10.AS1	-1.88026402	6.33642E-06	downregulated
LRRC36	-1.879827718	2.79971E-12	downregulated

NKD2	-1.87875632	7.78913E-15	downregulated
LINC01549	-1.87872086	3.63813E-06	downregulated
DRD1	-1.877112137	9.62404E-08	downregulated
CTNNA2	-1.873748605	0.000269131	downregulated
F11	-1.869462953	3.72215E-05	downregulated
SLC51B	-1.867804954	1.44103E-11	downregulated
KIF25.AS1	-1.863083707	7.71487E-05	downregulated
CEL	-1.86144691	5.2924E-05	downregulated
PAX7	-1.854716763	0.000757398	downregulated
LINC00525	-1.854086087	1.68512E-14	downregulated
SCT	-1.84663506	4.6172E-07	downregulated
SHISA6	-1.845840712	4.07778E-05	downregulated
RASL10B	-1.843543683	4.06801E-09	downregulated
SMIM2.AS1	-1.83927636	2.65549E-11	downregulated
LINC02223	-1.837598454	2.35437E-07	downregulated
ALKAL1	-1.834515807	3.8232E-11	downregulated
ZNF541	-1.83131055	8.23445E-08	downregulated
TYRP1	-1.827289496	1.59555E-09	downregulated
NXPE4	-1.825369212	1.77599E-05	downregulated
NPY5R	-1.822195889	6.23486E-05	downregulated
SLC4A10	-1.818772658	2.9973E-05	downregulated
IZUMO2	-1.815021942	2.4252E-06	downregulated
EVX2	-1.813591909	1.59376E-05	downregulated
LINC01996	-1.813347696	5.01636E-08	downregulated
LINC00942	-1.813260678	1.12253E-08	downregulated
NPFFR1	-1.809763785	6.97466E-14	downregulated
LINC02943	-1.806684548	8.28744E-08	downregulated
LIPC	-1.806432797	1.39461E-05	downregulated
RPS4XP7	-1.805241734	9.53222E-13	downregulated
LINC01239	-1.800850991	0.000170417	downregulated
PTCHD3P2	-1.800665977	1.19729E-05	downregulated
TINAG	-1.800001488	1.35792E-13	downregulated
C20orf204	-1.796486951	2.787E-06	downregulated
LRRC52.AS1	-1.796058752	5.47631E-05	downregulated
TG	-1.795313049	7.16064E-10	downregulated
DLX5	-1.792921624	2.27966E-05	downregulated
TRD.AS1	-1.792098629	5.53522E-06	downregulated
SMTNL2	-1.789077643	2.94547E-08	downregulated
R3HDML.AS1	-1.788533067	1.40727E-13	downregulated
REEP1	-1.786646115	7.32733E-09	downregulated
CLGN	-1.786322489	6.1712E-07	downregulated
PDE6A	-1.783468787	1.76721E-07	downregulated
LINC02936	-1.77977527	2.81652E-22	downregulated
KRTAP3.1	-1.776495533	0.00026342	downregulated
RIPPLY3	-1.776241327	1.5627E-09	downregulated
R3HDML	-1.775440666	2.06749E-11	downregulated
VSTM2B	-1.772522891	0.000536522	downregulated
WNT11	-1.771903316	1.11841E-06	downregulated
CLDN6	-1.771621487	0.00088455	downregulated
C10orf67	-1.770315738	1.05583E-07	downregulated

CHGA	-1.767459678	0.000762002	downregulated
MOGAT3	-1.765793334	4.73835E-16	downregulated
DPEP1	-1.764787628	9.19556E-08	downregulated
IGKJ1	-1.762242084	0.000184406	downregulated
QPCT	-1.762078758	4.13817E-13	downregulated
LINC02814	-1.759646946	5.2878E-08	downregulated
WFDC10A	-1.755158585	5.20917E-07	downregulated
GCM1	-1.752734444	3.53964E-06	downregulated
CKMT2	-1.75244143	3.6614E-05	downregulated
TBX10	-1.750756771	1.61907E-07	downregulated
C1orf105	-1.74817891	1.1297E-08	downregulated
LINC03052	-1.747327618	1.56245E-09	downregulated
KCTD16	-1.745204726	1.26965E-05	downregulated
LINC01214	-1.737806254	1.94709E-08	downregulated
SULT1C4	-1.737728029	8.32748E-06	downregulated
LINC02672	-1.737470595	8.26324E-05	downregulated
KCNK17	-1.735946702	3.19262E-07	downregulated
LINC02600	-1.735837294	4.6854E-06	downregulated
PALM3	-1.733395146	7.05138E-08	downregulated
RBP1	-1.732154766	1.8643E-07	downregulated
SLC35D3	-1.728052861	3.91214E-06	downregulated
TMEM72	-1.726671127	1.50633E-07	downregulated
CNTN3	-1.725837696	8.30427E-07	downregulated
RASSF10.DT	-1.720293188	8.02605E-11	downregulated
ZP2	-1.717759828	3.50036E-06	downregulated
FAM83C	-1.710089343	4.95289E-05	downregulated
SLC2A12	-1.708511411	1.81507E-12	downregulated
CLSTN2	-1.704154283	1.86838E-05	downregulated
RN7SL272P	-1.698064122	7.31429E-05	downregulated
GLRA2	-1.693375754	7.58301E-05	downregulated
LINC00365	-1.691435298	1.14321E-13	downregulated
LEFTY1	-1.689279732	5.82447E-05	downregulated
TRIM54	-1.688616892	7.75155E-05	downregulated
KCNT1	-1.688413179	2.24258E-06	downregulated
LY6G6E	-1.68295091	3.38148E-07	downregulated
LINC00964	-1.679943125	6.30036E-06	downregulated
NOX1	-1.678003959	3.1017E-12	downregulated
ABCB1	-1.675271072	8.94729E-10	downregulated
SOAT2	-1.674401242	0.000107629	downregulated
LRIT2	-1.674242144	5.97675E-08	downregulated
NEUROG2.AS1	-1.671912467	0.000368447	downregulated
HSD3B2	-1.671751361	0.000389115	downregulated
SLC28A1	-1.67090529	7.95015E-06	downregulated
FZD10.AS1	-1.669515404	1.76814E-06	downregulated
TH	-1.667827311	2.27086E-08	downregulated
UCA1	-1.664975872	1.26533E-05	downregulated
CYP4F2	-1.657553953	2.16592E-06	downregulated
ZNF470.DT	-1.652248116	3.64153E-14	downregulated
L1CAM	-1.651078552	2.5496E-05	downregulated
RPL10L	-1.650412992	1.75743E-07	downregulated

CCDC33	-1.649978095	2.25977E-07	downregulated
SELENBP1	-1.648356512	2.74982E-10	downregulated
SPACA3	-1.646634442	3.57652E-06	downregulated
PAH	-1.644209726	4.61931E-05	downregulated
C5orf52	-1.642856729	7.94883E-11	downregulated
SLC6A18	-1.639061562	4.50062E-06	downregulated
LY6G6F	-1.636843699	4.85899E-07	downregulated
VAV3	-1.636709943	8.949E-13	downregulated
SV2C	-1.635899543	5.27555E-06	downregulated
SATB2.AS1	-1.634253137	2.70251E-13	downregulated
CBLN1	-1.631461425	1.15685E-05	downregulated
SPATA25	-1.631007182	1.69999E-17	downregulated
OGDHL	-1.63032641	6.33578E-06	downregulated
TMEM150C	-1.625599033	8.84749E-12	downregulated
TNFRSF19	-1.624930433	2.83916E-06	downregulated
IGLV7.46	-1.623813962	0.000581638	downregulated
SLC19A3	-1.618881947	3.93517E-09	downregulated
CBY2	-1.618743617	3.43417E-07	downregulated
CES1	-1.613418205	6.83262E-05	downregulated
RGN	-1.611604593	7.26991E-06	downregulated
CEACAM7	-1.609786636	0.000163886	downregulated
GJC3	-1.608879271	7.44327E-11	downregulated
LINC01226	-1.608754306	4.13594E-14	downregulated
CAPS	-1.607583598	4.59972E-09	downregulated
CCL25	-1.604716832	0.007613261	downregulated
HCN1	-1.599876854	3.10415E-05	downregulated
GGT4P	-1.599148818	4.27166E-06	downregulated
PTPRD	-1.591728425	3.62531E-09	downregulated
CDHR1	-1.59156708	1.11702E-06	downregulated
LINC02938	-1.590771468	2.68726E-07	downregulated
OXGR1	-1.590008746	1.30818E-07	downregulated
RPRM	-1.589910573	1.13117E-05	downregulated
SMPX	-1.589726613	0.000147194	downregulated
IHH	-1.586277896	1.57546E-14	downregulated
CECR2	-1.584851726	2.51101E-05	downregulated
IL17F	-1.584030065	5.87821E-05	downregulated
CES1P1	-1.581885242	0.000243312	downregulated
PRAC1	-1.576442545	0.007414658	downregulated
NRXN2	-1.575751898	2.26232E-08	downregulated
PRR15	-1.573226759	1.83181E-24	downregulated
LINC02966	-1.569988066	2.02199E-08	downregulated
CPB1	-1.567376072	0.001214675	downregulated
IGFL4	-1.567149248	6.56524E-08	downregulated
SOSTDC1	-1.565554623	1.42438E-06	downregulated
TMEM176A	-1.559919	6.70056E-16	downregulated
MAGEA6	-1.558416899	0.017992121	downregulated
RNF43	-1.554100074	1.08741E-20	downregulated
LRRC2	-1.553512063	7.63087E-13	downregulated
LINC01555	-1.548926833	2.98371E-09	downregulated
SERPIND1	-1.547512533	0.000129758	downregulated

AIFM3	-1.543284684	9.30616E-11	downregulated
ISX	-1.540559274	3.01008E-08	downregulated
LINC01036	-1.54028657	0.001046408	downregulated
KRT36	-1.537553956	1.84127E-06	downregulated
LINC02820	-1.536734327	0.000529686	downregulated
TMEM63C	-1.535355615	5.41228E-08	downregulated
SERPINA10	-1.534313034	0.000156352	downregulated
CYP4F23P	-1.534306786	1.1034E-06	downregulated
SLC38A4	-1.532903989	4.6744E-06	downregulated
CLVS2	-1.528928482	0.000664319	downregulated
PNPLA1	-1.52881	3.86973E-09	downregulated
ABAT	-1.527038694	4.27809E-13	downregulated
VANGL2	-1.524117953	2.09082E-09	downregulated
LCN12	-1.522706325	5.12159E-11	downregulated
EGF	-1.52081017	0.000283629	downregulated
TRIM63	-1.519500293	7.5462E-05	downregulated
MYOM3	-1.518592436	1.73375E-10	downregulated
TDRKH.AS1	-1.518194369	9.6299E-20	downregulated
SESN1	-1.513099081	1.741E-16	downregulated
B3GAT1.DT	-1.511289829	1.05994E-08	downregulated
EPHA7	-1.511253065	0.000253648	downregulated
TNMD	-1.508469355	6.16225E-06	downregulated
RXFP4	-1.501112573	2.57129E-11	downregulated
DYNLRB2.AS1	-1.500113227	2.40322E-06	downregulated
IGHV3.64	-1.49949509	0.002224317	downregulated
SALL4	-1.49673616	5.12159E-11	downregulated
CACNA1E	-1.494612198	0.000450794	downregulated
DIO3	-1.491535247	2.17191E-06	downregulated
RSPO2	-1.490681384	3.17686E-06	downregulated
ABCC2	-1.490447184	7.20129E-06	downregulated
FGGY	-1.488783	2.05045E-07	downregulated
SLC7A10	-1.480576927	0.00027855	downregulated
LINC03046	-1.479338666	0.000267005	downregulated
FTCD	-1.478556076	0.000585609	downregulated
LINC01942	-1.477722611	1.48866E-06	downregulated
AREG	-1.468432235	2.7477E-09	downregulated
KCNJ16	-1.467957244	0.000158313	downregulated
EPDR1	-1.467858895	3.35139E-15	downregulated
HOXD12	-1.464678216	3.73083E-05	downregulated
LINC01807	-1.464348296	9.53979E-05	downregulated
OBP2B	-1.462757638	0.004255246	downregulated
GLYATL1	-1.462697049	3.50283E-07	downregulated
FREM2	-1.462009098	7.75155E-05	downregulated
NPY1R	-1.455149779	8.65408E-05	downregulated
PNMT	-1.452689899	0.002007045	downregulated
NBPF6	-1.451015975	0.000117288	downregulated
IGSF23	-1.45030764	6.71617E-08	downregulated
ARID3A	-1.449825918	2.11415E-14	downregulated
KL	-1.446779656	1.33377E-06	downregulated
CKB	-1.446357511	4.03165E-06	downregulated

FREM1	-1.446123336	6.07282E-05	downregulated
PPP1R14D	-1.442709298	2.4225E-12	downregulated
CFTR	-1.441584957	6.17378E-12	downregulated
LINC01485	-1.441432267	2.47092E-05	downregulated
RASGRF1	-1.436742167	3.06452E-06	downregulated
CLEC2L	-1.432622228	0.000149284	downregulated
OPCML	-1.431950304	3.5152E-07	downregulated
ID1	-1.429535707	1.28344E-08	downregulated
LINC02538	-1.425681932	6.58307E-09	downregulated
SCARA5	-1.425616865	2.86172E-05	downregulated
LINC02950	-1.422091286	1.2195E-06	downregulated
CTSV	-1.421912582	1.51805E-11	downregulated
TCP10L2	-1.41527907	2.95983E-07	downregulated
HOXD10	-1.414977736	3.74497E-05	downregulated
OCA2	-1.405877	8.836E-05	downregulated
LINC02413	-1.405746233	0.000368609	downregulated
SLC7A9	-1.405238009	0.00063574	downregulated
IGLV10.54	-1.40389672	0.002464498	downregulated
UGT2B17	-1.403761512	0.006928688	downregulated
MEX3A	-1.399614583	7.6217E-14	downregulated
ADH1C	-1.397136427	0.000532064	downregulated
BMP3	-1.396949104	0.000279076	downregulated
KLRG2	-1.39605386	0.000787619	downregulated
LINC00654	-1.394232105	3.89867E-07	downregulated
ZNF606	-1.392472048	4.09127E-11	downregulated
GABRR1	-1.389029702	0.001584771	downregulated
RNLS	-1.388982894	1.02324E-12	downregulated
PMEPA1	-1.388479395	2.40676E-13	downregulated
TLE2	-1.386532549	6.30778E-12	downregulated
NODAL	-1.386322944	2.30648E-06	downregulated
LINC00940	-1.382844192	1.8142E-05	downregulated
NR1I2	-1.378278846	9.68786E-14	downregulated
AKR1C4	-1.373497062	0.000742693	downregulated
CST1	-1.373234879	0.000370986	downregulated
KIF1A	-1.372742693	0.002429344	downregulated
PGAP3	-1.372726608	7.90504E-08	downregulated
IGKV1D.12	-1.363810627	0.002480357	downregulated
RPSAP71	-1.362308004	0.000542554	downregulated
HOXA2	-1.361815145	4.85385E-08	downregulated
ATP12A	-1.361051552	0.005849708	downregulated
WASF3	-1.360184931	7.70807E-06	downregulated
NXPH1	-1.359119867	0.000268611	downregulated
HOXD11	-1.3578923	0.00012425	downregulated
ST6GAL2	-1.355933712	0.000152403	downregulated
RASSF10	-1.355359771	6.13944E-07	downregulated
HOGA1	-1.35220374	1.24805E-06	downregulated
PLAGL2	-1.351142544	3.36E-20	downregulated
FABP6	-1.351000955	1.80422E-06	downregulated
MYEF2	-1.35052482	2.82627E-07	downregulated
SLC38A11	-1.349685738	0.000410743	downregulated

UGT1A10	-1.348768046	1.17946E-05	downregulated
SEPHS1P6	-1.348624658	1.03299E-06	downregulated
SFTA2	-1.347331645	0.0001832	downregulated
AFP	-1.345119346	3.69794E-05	downregulated
LGR6	-1.344754724	1.39722E-05	downregulated
SLC27A6	-1.3447498	0.000296609	downregulated
DEFB1	-1.344491062	2.70663E-05	downregulated
CST2	-1.344394934	5.59725E-05	downregulated
SGK2	-1.340965306	2.59386E-11	downregulated
ZNF853	-1.339402579	2.08766E-09	downregulated
LINC02924	-1.338657032	4.16205E-11	downregulated
SYT7	-1.338532546	5.86025E-13	downregulated
ZNF793.AS1	-1.338029349	4.43972E-06	downregulated
GGT7	-1.336513485	7.63087E-13	downregulated
RP1	-1.334328218	0.000133299	downregulated
CELF5	-1.331438364	4.67062E-06	downregulated
KRT12	-1.329874316	8.0834E-05	downregulated
LINC02982	-1.329434049	1.23272E-09	downregulated
ANO9	-1.329374208	4.62315E-13	downregulated
PLCB4	-1.328432178	5.21166E-08	downregulated
ENGASE	-1.327029706	1.84549E-16	downregulated
MMP11	-1.32515063	7.22468E-07	downregulated
FAM131B.AS1	-1.324551498	0.000676736	downregulated
LINC00526	-1.322551845	1.0044E-11	downregulated
DUSP15	-1.320924847	5.47762E-07	downregulated
C1GALT1C1L	-1.32072843	1.00696E-07	downregulated
CKM	-1.319822584	7.05764E-06	downregulated
WNT5B	-1.317025916	1.48392E-09	downregulated
CDK5R2	-1.316961903	0.000659148	downregulated
HOXA10.AS	-1.312691184	2.21458E-06	downregulated
ASB5	-1.312207032	0.003782719	downregulated
HCRT	-1.311703638	0.000324114	downregulated
EPCAM.DT	-1.311490581	4.78887E-14	downregulated
TMPRSS13	-1.30968399	3.42009E-06	downregulated
GLP2R	-1.308326426	2.47841E-08	downregulated
ACTBP1	-1.308287041	1.06428E-05	downregulated
BEX2	-1.308220675	0.000161916	downregulated
GABRA4	-1.307720049	0.002438516	downregulated
SFRP5	-1.306112529	0.002557769	downregulated
LINC02594	-1.306105282	1.96658E-06	downregulated
MME	-1.30549629	0.000121135	downregulated
CST6	-1.305071663	0.002139359	downregulated
AKNAD1	-1.303869783	1.64453E-08	downregulated
FER1L4	-1.302380582	3.91202E-06	downregulated
GPC5	-1.301233842	0.001340765	downregulated
ACTG1P25	-1.301084535	1.27634E-13	downregulated
HMGN2P15	-1.296775792	1.76259E-08	downregulated
EVX1	-1.294101501	0.000185403	downregulated
NEURL2	-1.291959214	1.66554E-14	downregulated
TMEM82	-1.289532125	6.96704E-06	downregulated

LIN7A	-1.287574318	5.68312E-05	downregulated
RPS6KA6	-1.28676623	4.34999E-06	downregulated
FAM178B	-1.286700579	9.45417E-06	downregulated
HSD3B1	-1.28546933	0.000171618	downregulated
GSPT2	-1.285142615	7.49063E-09	downregulated
LINC03086	-1.283653872	0.000205898	downregulated
SHROOM2	-1.283018764	3.71319E-09	downregulated
LEP	-1.282891993	0.012128862	downregulated
PLUT	-1.282133823	3.96582E-06	downregulated
CYP24A1	-1.28123523	0.000531257	downregulated
TMC2	-1.28030832	1.73217E-05	downregulated
PPP1R14C	-1.280196176	4.20349E-08	downregulated
RXRG	-1.280151982	1.84438E-05	downregulated
PNLIPRP2	-1.279757676	0.000365843	downregulated
LINC02512	-1.278617455	0.003420125	downregulated
NKX6.3	-1.276684118	0.021729653	downregulated
AMACR	-1.27445803	1.2202E-12	downregulated
**MTATP8P2**	-1.272449202	0.024984042	downregulated
USP2	-1.272161067	1.34684E-07	downregulated
GCAWKR	-1.271690623	2.0856E-05	downregulated
PADI1	-1.270346175	0.000100454	downregulated
SEZ6L	-1.269350103	0.002046769	downregulated
MIR3131	-1.268521314	1.49187E-09	downregulated
SIX2	-1.266119365	0.001206848	downregulated
WDR35.DT	-1.265660782	2.10686E-08	downregulated
BRSK2	-1.265126886	8.70725E-05	downregulated
TMEM176B	-1.265100853	1.77034E-14	downregulated
SULT1E1	-1.260342034	0.010255217	downregulated
SNCAIP	-1.260158609	3.89562E-06	downregulated
POFUT1	-1.258933556	7.71446E-21	downregulated
MUC20P1	-1.25867214	4.56819E-08	downregulated
ARHGDIG	-1.258129826	0.000279076	downregulated
NOVA1.DT	-1.257668269	0.001195072	downregulated
KHDRBS3	-1.255401269	3.14115E-11	downregulated
KCNG1	-1.255302058	0.000367601	downregulated
GGH	-1.25436026	4.27837E-12	downregulated
TPTE2P1	-1.250825432	7.05357E-07	downregulated
LINC02848	-1.248164723	1.9266E-05	downregulated
FBXO2	-1.24788338	0.000637075	downregulated
SUSD3	-1.247642165	9.82959E-08	downregulated
PRSS23	-1.247517704	3.17388E-13	downregulated
SLC22A31	-1.245959334	0.008350976	downregulated
CYP4F29P	-1.244198584	0.000182921	downregulated
LINC02568	-1.239654854	9.8849E-05	downregulated
NALF2	-1.238755943	0.001478066	downregulated
KISS1	-1.237470747	2.36993E-06	downregulated
KRT20	-1.237289963	1.1599E-05	downregulated
TMEM132D	-1.236878699	0.000101736	downregulated
LINC02418	-1.233616992	0.000423573	downregulated
CABCOCO1	-1.232586587	3.06363E-06	downregulated

CARD11	-1.230883737	6.14718E-05	downregulated
AGMO	-1.230400735	0.000164324	downregulated
PI16	-1.227979272	0.004181516	downregulated
NRCAM	-1.227793415	0.000120249	downregulated
ADGB	-1.22716965	0.000136203	downregulated
NTRK2	-1.226506339	0.003059803	downregulated
ODAM	-1.225825714	0.000892416	downregulated
GNGT1	-1.223498021	9.44646E-05	downregulated
LINC00552	-1.222999076	0.000405719	downregulated
VIP	-1.222428967	0.001132808	downregulated
AGT	-1.221958087	1.96203E-09	downregulated
CHST13	-1.221096443	6.09707E-05	downregulated
LRRC19	-1.221077703	6.42848E-07	downregulated
GUSBP5	-1.219333372	3.13627E-07	downregulated
CCDC170	-1.218982799	2.0385E-07	downregulated
ZSWIM3	-1.218693049	1.15396E-19	downregulated
RPSAP53	-1.217049018	0.004347944	downregulated
TMEM236	-1.216809414	1.93023E-05	downregulated
CFAP74	-1.215619803	4.30923E-05	downregulated
MAPRE3	-1.214278036	2.48162E-18	downregulated
CHRNA1	-1.212927336	1.64706E-07	downregulated
SEMA5A	-1.212009316	2.96114E-12	downregulated
OSER1.DT	-1.21141969	1.56862E-11	downregulated
MCMDC2	-1.210978085	9.34596E-09	downregulated
DDC	-1.205882651	3.09759E-09	downregulated
KRT75	-1.205663839	0.002134892	downregulated
NFE2	-1.204558026	0.000893009	downregulated
TDRD12	-1.204556214	0.000614383	downregulated
DLX6.AS1	-1.202870692	0.002846086	downregulated
SPINK6	-1.202719106	0.002985507	downregulated
NKILA	-1.202261361	6.12452E-09	downregulated
CELF3	-1.201951403	0.000703432	downregulated
LINC02487	-1.201809381	9.5166E-10	downregulated
GRK1	-1.19992927	0.000167104	downregulated
RAMP2.AS1	-1.198861381	5.21711E-07	downregulated
MAGEA11	-1.197280648	0.027060803	downregulated
SSUH2	-1.196157729	1.12193E-05	downregulated
SLC6A3	-1.194494543	0.001851587	downregulated
SATB1.AS1	-1.193816516	8.87584E-05	downregulated
ERICH4	-1.191017514	0.002907554	downregulated
GREM2	-1.189877475	0.000361255	downregulated
TCP11	-1.189035375	0.015175009	downregulated
IGLV2.18	-1.188878657	0.006491635	downregulated
HTR4	-1.187994726	0.000522505	downregulated
ISLR2	-1.187797735	6.55472E-07	downregulated
SLC5A6	-1.186472527	1.59609E-19	downregulated
AOAH	-1.184737472	8.37948E-09	downregulated
NKAIN3	-1.184162542	3.50153E-05	downregulated
FMN2	-1.183789414	0.001796038	downregulated
ALKAL2	-1.183400883	3.85618E-05	downregulated

EMX2	-1.182779069	0.006070587	downregulated
AKAP4	-1.182597719	0.007869779	downregulated
RLN2	-1.182156758	2.01429E-06	downregulated
STMN2	-1.180727533	0.00031535	downregulated
HOXA3	-1.179184831	5.24993E-09	downregulated
GRPR	-1.178164269	0.002854503	downregulated
TCF7	-1.177674843	8.10136E-19	downregulated
GLOD5	-1.17766693	3.6946E-10	downregulated
ATP9A	-1.177551692	8.50132E-17	downregulated
LY6G6C	-1.177179597	7.87434E-05	downregulated
CLDN15	-1.175607451	4.33692E-06	downregulated
IL34	-1.174418014	1.07624E-08	downregulated
CHODL	-1.173363072	8.14041E-05	downregulated
HSD11B2	-1.173031968	3.43297E-10	downregulated
BPI	-1.172500165	1.84682E-05	downregulated
ADGRF4	-1.171419307	1.52511E-07	downregulated
GUCY2GP	-1.167264331	2.14171E-07	downregulated
CAPN6	-1.167037663	0.007005975	downregulated
NHERF4	-1.165660566	2.69485E-07	downregulated
UPF3AP2	-1.165438753	1.90538E-09	downregulated
LRRC43	-1.165286314	9.51766E-08	downregulated
PNLIPRP1	-1.164182901	1.1043E-05	downregulated
LINC02188	-1.163761393	0.005347528	downregulated
NCKAP5	-1.163055477	9.08783E-06	downregulated
THSD7B	-1.162218101	0.003989133	downregulated
ARL11	-1.160962314	8.79518E-06	downregulated
CBLN2	-1.159726788	1.22386E-05	downregulated
NPY4R	-1.159185136	8.39686E-06	downregulated
LINC02967	-1.158725474	3.36392E-10	downregulated
KILH	-1.157886268	0.007726284	downregulated
DPYSL5	-1.156594171	0.010043375	downregulated
TSPAN6	-1.156586066	2.29573E-15	downregulated
LINC02747	-1.154717787	1.46998E-09	downregulated
RUNDC3A	-1.154010965	0.002279188	downregulated
HS3ST5	-1.151251825	0.002480357	downregulated
SPATC1L	-1.151034736	4.35761E-06	downregulated
LINC02057	-1.149841638	4.62203E-05	downregulated
MPP1	-1.149009269	2.36939E-09	downregulated
SLC22A2	-1.148866519	0.000101362	downregulated
PACRG	-1.148740962	3.36061E-06	downregulated
CHN2	-1.146381778	0.00027616	downregulated
TNFSF11	-1.144918919	1.8643E-07	downregulated
MLH1	-1.14467587	1.25282E-22	downregulated
STK4.DT	-1.143326188	2.02809E-08	downregulated
ABCC6P2	-1.143274165	7.74349E-08	downregulated
HPSE2	-1.142724496	2.21466E-05	downregulated
WNT6	-1.142504077	0.003272203	downregulated
HSPH1	-1.142175304	1.52432E-16	downregulated
RPS16P2	-1.141023607	5.02359E-06	downregulated
CEACAM8	-1.139696464	0.001323684	downregulated

SLC10A4	-1.137944499	9.06763E-05	downregulated
PABPC1L	-1.137513191	7.40819E-08	downregulated
LINC00540	-1.137496065	0.006269783	downregulated
SLC14A2	-1.13590791	0.005710405	downregulated
CEBPA	-1.134180412	1.87574E-12	downregulated
TMEM249	-1.133003391	0.000256748	downregulated
THBS4	-1.132263262	0.005817778	downregulated
CH25H	-1.131920826	1.16581E-07	downregulated
PGBD5	-1.131712499	2.76214E-06	downregulated
ARFGEF1.DT	-1.12899016	1.77509E-08	downregulated
LUZP2	-1.128440195	0.000179245	downregulated
ATOH8	-1.127789406	1.01842E-05	downregulated
KRTAP4.1	-1.127351408	2.12484E-05	downregulated
ROBO2	-1.12615252	7.16759E-05	downregulated
PTP4A3	-1.125654231	7.90618E-08	downregulated
CPE	-1.125530239	1.9165E-05	downregulated
FABP3	-1.123492328	0.000362332	downregulated
DDAH2	-1.122701841	2.51209E-17	downregulated
SERPINE2	-1.121846589	1.47759E-10	downregulated
LPL	-1.121222049	4.24225E-05	downregulated
NKX2.1	-1.119837718	0.048219033	downregulated
KIF25	-1.119365613	0.000218314	downregulated
PIGZ	-1.119350535	1.29594E-08	downregulated
LINC01605	-1.117163805	8.72418E-09	downregulated
ARSLP1	-1.116246956	0.00129643	downregulated
MYL3	-1.115023318	0.000153182	downregulated
FCGRT	-1.114969389	2.98009E-13	downregulated
RTL8A	-1.11210964	7.49801E-08	downregulated
RHPN1.AS1	-1.111909531	7.13658E-09	downregulated
LINC01315	-1.111267996	2.85782E-10	downregulated
OXR1.AS1	-1.110736341	7.11829E-08	downregulated
KRTAP5.1	-1.110373153	1.43457E-08	downregulated
PMFBP1	-1.110102939	6.84683E-08	downregulated
LINC02562	-1.105918727	2.63474E-10	downregulated
ANKRD33BP1	-1.105707842	8.89686E-05	downregulated
SELENOP	-1.105649287	1.37938E-05	downregulated
JCHAIN	-1.105311723	0.007272763	downregulated
ZNF536	-1.104384464	0.00014666	downregulated
NRXN3	-1.104269977	0.000167359	downregulated
MUC20	-1.103406686	8.1274E-10	downregulated
GAS2	-1.101746275	9.60355E-09	downregulated
TTC23L.AS1	-1.10037052	9.946E-09	downregulated
TMEM35A	-1.099992481	9.5729E-05	downregulated
APOLD1	-1.097665197	2.51783E-05	downregulated
NEK3	-1.097041914	3.45626E-15	downregulated
TREH	-1.096327057	6.37685E-05	downregulated
LINC01082	-1.093404639	0.000144941	downregulated
RNA5SP111	-1.092906835	0.004180617	downregulated
LAPTM4B	-1.092120546	4.98434E-10	downregulated
HS1BP3.IT1	-1.091879567	4.15053E-06	downregulated

OIT3	-1.091867566	5.25917E-05	downregulated
LPAL2	-1.090489802	2.98167E-12	downregulated
CADPS	-1.089121844	2.88496E-05	downregulated
TGFBI	-1.087428346	1.51603E-08	downregulated
CLCN2	-1.086486103	4.53827E-16	downregulated
HAS2.AS1	-1.086046115	9.43182E-05	downregulated
MLXIPL	-1.084595389	9.61492E-07	downregulated
CEACAM6	-1.082516396	2.61733E-07	downregulated
APOD	-1.082164047	0.001000007	downregulated
FUZ	-1.081843649	5.74777E-08	downregulated
SOWAHA	-1.081824042	8.31044E-09	downregulated
PLA2G4F	-1.081534162	1.13162E-08	downregulated
LINC02266	-1.078326559	0.007085421	downregulated
KCNMB4	-1.07778566	6.29537E-05	downregulated
LINC00871	-1.076638235	0.00483088	downregulated
EREG	-1.076588253	0.001722696	downregulated
ZNF423	-1.075743564	2.22771E-07	downregulated
C1QTNF12	-1.075585442	4.73216E-05	downregulated
CYCSP6	-1.075386527	0.001231013	downregulated
GFRA3	-1.075307694	0.001198901	downregulated
IGFBPL1	-1.074922606	0.001243521	downregulated
GRB7	-1.07445434	8.07639E-06	downregulated
LINC01597	-1.073516843	0.000507401	downregulated
CD300LG	-1.072487463	0.017772517	downregulated
MMP7	-1.071479524	0.002460653	downregulated
SLC6A19	-1.07040437	0.01921303	downregulated
FGF18	-1.069826454	1.59388E-07	downregulated
VSTM2A	-1.069208645	0.005936539	downregulated
SPMIP4	-1.068637614	1.38012E-15	downregulated
C12orf56	-1.068386002	0.011320861	downregulated
MADCAM1	-1.067920605	3.74445E-05	downregulated
FRMD1	-1.067824439	0.000171186	downregulated
RGR	-1.067390764	0.01391662	downregulated
CYP39A1	-1.067066426	1.80393E-09	downregulated
CERS4	-1.065717247	3.70137E-05	downregulated
LINC00607	-1.063375848	0.012735454	downregulated
DPP10	-1.063280579	0.016340732	downregulated
PID1	-1.060979138	1.45618E-06	downregulated
ZNF503	-1.060447947	4.68108E-11	downregulated
IGHA1	-1.060163848	0.01324984	downregulated
ANKDD1B	-1.059992489	1.06776E-06	downregulated
ZNF662	-1.058691788	0.000112054	downregulated
ACTL8	-1.058576218	0.02253947	downregulated
RPSAP19	-1.057478565	0.001761824	downregulated
CLDN3	-1.05741847	4.72154E-08	downregulated
COL17A1	-1.056661643	1.33224E-05	downregulated
FGF19	-1.056650524	0.0197135	downregulated
EN2	-1.05587653	0.011923457	downregulated
FITM2	-1.055629018	1.18469E-15	downregulated
FAM3B	-1.054235958	0.010668309	downregulated

GPR150	-1.053967151	8.11489E-06	downregulated
TUBA5P	-1.053207495	1.83514E-11	downregulated
BTNL3	-1.052784589	0.000267747	downregulated
RDH12	-1.052166117	1.55267E-06	downregulated
LIME1	-1.051854899	4.1356E-08	downregulated
DDX27	-1.051835235	3.62913E-21	downregulated
EWSAT1	-1.050890291	2.58614E-05	downregulated
LINC02864	-1.050612345	0.049566222	downregulated
CCDC88B	-1.050307401	1.56301E-05	downregulated
LRRC37A17P	-1.050164904	0.000406053	downregulated
LINC01003	-1.049700217	2.7381E-14	downregulated
DHRS12	-1.04959684	2.8479E-15	downregulated
ITGB8.AS1	-1.048258814	9.51357E-10	downregulated
DSG1	-1.047434548	0.000103696	downregulated
ADD3.AS1	-1.047433798	7.24058E-07	downregulated
RAB32	-1.047050899	5.08459E-12	downregulated
CDHR5	-1.046643186	2.49952E-07	downregulated
ZNF610	-1.045840127	1.52782E-06	downregulated
VIL1	-1.044815929	2.50199E-15	downregulated
GAPDHP71	-1.044117074	7.32307E-05	downregulated
FOXD2	-1.04346836	3.56195E-08	downregulated
TAS1R3	-1.042485997	0.000186543	downregulated
DAPK2	-1.042445105	5.20306E-12	downregulated
ARSL	-1.042416712	2.36889E-05	downregulated
TFCP2L1	-1.041436139	1.32042E-07	downregulated
TTPA	-1.040709046	8.79826E-06	downregulated
TF	-1.040152758	0.003959493	downregulated
CWH43	-1.038937754	0.00859258	downregulated
NINL	-1.036947845	9.39261E-05	downregulated
COLEC10	-1.035059213	0.006743328	downregulated
PRDX5	-1.034977142	1.46788E-09	downregulated
ZNRF3	-1.03356851	7.78559E-15	downregulated
SNTN	-1.033389744	4.74646E-07	downregulated
SPIRE2	-1.033254752	1.36194E-14	downregulated
FCER1A	-1.031474492	0.000102381	downregulated
IGLV1.51	-1.029195863	0.01147756	downregulated
CLDN9	-1.026735768	0.000437579	downregulated
IGHV3.72	-1.026352614	0.009581262	downregulated
ADAMTSL2	-1.024602301	3.08851E-08	downregulated
GRIK2	-1.022619173	0.0012474	downregulated
SNHG18	-1.021846113	1.34072E-10	downregulated
SLC22A3	-1.021515976	3.4728E-11	downregulated
IYD	-1.021166625	5.39551E-09	downregulated
SMOC2	-1.020256488	0.000308922	downregulated
ADRA2C	-1.020217074	0.00114184	downregulated
NHLRC1	-1.019607746	6.30696E-07	downregulated
MOCS3	-1.019359276	7.44181E-19	downregulated
RPL12P14	-1.017208377	4.73644E-08	downregulated
MYL12BP2	-1.017068579	2.3045E-05	downregulated
CDX2	-1.016651935	3.54057E-10	downregulated

LINC02298	-1.015911012	2.86131E-09	downregulated
SPIN3	-1.015713165	1.97295E-07	downregulated
CSTL1	-1.015642191	0.000746218	downregulated
LINC01579	-1.014776556	5.19524E-05	downregulated
IGHV3.64D	-1.014091205	0.031798731	downregulated
CARTPT	-1.01408896	0.014668814	downregulated
SERP2	-1.013636679	9.7215E-05	downregulated
SLC23A1	-1.013105999	8.05503E-07	downregulated
DDO	-1.012929524	8.36661E-05	downregulated
PDGFA.DT	-1.011449306	0.000130483	downregulated
APIP	-1.010813616	1.73375E-10	downregulated
CYP2W1	-1.007462763	0.017208571	downregulated
MYH7B	-1.00664915	0.004347944	downregulated
RPL31P34	-1.006615914	5.16124E-07	downregulated
CDH26	-1.004900601	9.01256E-05	downregulated
MT.TT	-1.004462629	0.000562323	downregulated
NANOS3	-1.004376033	0.001273286	downregulated
EMID1	-1.004164482	5.12333E-07	downregulated
LINC00858	-1.001552607	0.000391582	downregulated
HNRNPA1P26	-1.000418387	4.73144E-05	downregulated
CPNE1	-1.000181444	1.72341E-10	downregulated
SLN	1.000006351	0.000150695	upregulated
MCHR1	1.000705704	3.42522E-06	upregulated
ADAM12	1.002839008	0.000115677	upregulated
CD86	1.003196035	3.09486E-08	upregulated
LINC.PINT	1.003493698	4.61827E-06	upregulated
RORB	1.00354682	2.57506E-05	upregulated
SMC3P1	1.003898596	3.19233E-10	upregulated
SPRED3	1.00502254	1.39961E-10	upregulated
C4orf50	1.005616555	1.11506E-05	upregulated
MEIS2	1.005955073	3.61617E-07	upregulated
THEMIS	1.005964193	8.90754E-08	upregulated
FBXO24	1.007129772	6.88126E-09	upregulated
TIGIT	1.007644235	1.19924E-07	upregulated
PLBD2	1.007971183	5.91576E-17	upregulated
CROCC2	1.008323754	0.000289424	upregulated
TCAM1P	1.009242001	0.004477946	upregulated
SIRPB2	1.009386912	7.12215E-08	upregulated
SMCHD1	1.01033892	2.00327E-19	upregulated
POLR2A	1.010455912	2.35013E-19	upregulated
CTXN1	1.010712364	1.21339E-05	upregulated
PCDHGB8P	1.010731549	3.58146E-05	upregulated
LPCAT1	1.010740961	2.34786E-18	upregulated
TXNDC11.AS1	1.011042773	6.54949E-10	upregulated
PCDHGA6	1.011342595	2.97604E-08	upregulated
SPDYE5	1.011867171	2.12899E-06	upregulated
TREM1	1.012278013	9.65226E-05	upregulated
IGKV5.2	1.012845692	0.001914633	upregulated
NT5DC3	1.014040213	3.61415E-18	upregulated
PYDC2.AS1	1.015316886	0.005968958	upregulated

ADGRE4P	1.015397791	1.60202E-06	upregulated
GCKR	1.015554296	1.89521E-06	upregulated
ZNF232.AS1	1.01626165	2.56303E-12	upregulated
THBS2.AS1	1.016297795	4.58663E-06	upregulated
FAM171A2	1.016345023	9.58793E-10	upregulated
SLC11A1	1.017659513	4.59314E-07	upregulated
TAFA2	1.017792218	3.55985E-11	upregulated
IRF1	1.018038829	2.65474E-17	upregulated
HEPACAM2	1.018042009	0.006522035	upregulated
SLA	1.01815371	3.47005E-09	upregulated
SACS	1.01960247	8.25234E-13	upregulated
IFI30	1.019719159	2.55403E-10	upregulated
GALNT16.AS1	1.019924264	2.17665E-08	upregulated
NUSAP1	1.020028402	1.47556E-13	upregulated
PTPRC	1.020198253	5.8005E-07	upregulated
WNT10B	1.020215267	5.21195E-06	upregulated
CD7	1.02040074	1.71646E-06	upregulated
CYBB	1.020446221	2.68914E-07	upregulated
IL15	1.020591556	2.22899E-14	upregulated
F3	1.020861094	2.22919E-10	upregulated
DMXL2	1.02175228	3.75843E-16	upregulated
FER	1.022311406	1.90061E-15	upregulated
PLA2G2D	1.022475805	0.00087581	upregulated
C1QA	1.022961856	2.19138E-08	upregulated
CXCR2	1.023055542	3.11379E-05	upregulated
WNT4	1.023658461	9.16155E-07	upregulated
IGFBP1	1.024393738	0.001754719	upregulated
TNFAIP2	1.025224008	5.60188E-11	upregulated
RRH	1.025229893	3.4746E-07	upregulated
GK.AS1	1.025340485	7.3504E-05	upregulated
LMO4	1.025471146	9.90389E-18	upregulated
CCDC88A	1.025572523	1.08842E-08	upregulated
LINC02882	1.025682519	0.000922167	upregulated
SSTR5	1.027056918	0.000518441	upregulated
PCDH9	1.027158392	0.000179568	upregulated
MT1M	1.028322889	0.000337968	upregulated
C4BPB	1.028382758	1.32678E-07	upregulated
CFAP276	1.028928274	9.73674E-06	upregulated
RPSAP52	1.029061506	1.77611E-08	upregulated
PPM1K	1.029185876	3.57225E-16	upregulated
KRT7	1.02957007	0.001716702	upregulated
SBSPON	1.030205436	0.000247615	upregulated
OR2AG2	1.030561504	3.65404E-06	upregulated
AGPAT4	1.030831879	6.11068E-10	upregulated
GIMAP5	1.030923085	1.83217E-06	upregulated
ZKSCAN8P1	1.031038293	6.38471E-08	upregulated
GPR158	1.031161133	2.71362E-05	upregulated
GPR84	1.031899266	4.82633E-08	upregulated
GBP2	1.032408245	1.66368E-16	upregulated
ANKRD1	1.032556267	5.57374E-05	upregulated

NCF2	1.032602769	2.75645E-08	upregulated
STEAP4	1.032762863	1.1547E-05	upregulated
PRKAR2B	1.033024192	5.00308E-08	upregulated
ZNF543	1.033190761	2.76669E-10	upregulated
SLC9A7	1.033809418	5.84093E-10	upregulated
SLFN5	1.034460116	4.44803E-12	upregulated
PLCG1.AS1	1.034531683	6.90173E-07	upregulated
ALDH1A2	1.035015535	0.008876343	upregulated
GALNT14	1.035700298	0.000394993	upregulated
DNAJC19P5	1.035725112	2.184E-09	upregulated
RSPH1	1.035949651	2.71596E-12	upregulated
NR4A2	1.036396475	3.24506E-11	upregulated
ITGB7	1.037050709	1.48067E-09	upregulated
ARNT2	1.037616193	9.53943E-07	upregulated
FAM174B	1.038000376	1.42649E-09	upregulated
DTX2P1	1.039331324	1.10517E-05	upregulated
EFNA5	1.03949626	8.61628E-06	upregulated
**MARCO**	1.039788529	0.002110053	upregulated
HAR1A	1.039967406	1.69122E-05	upregulated
TNFSF13B	1.040550356	3.67653E-09	upregulated
GAPDHP60	1.040553785	1.31632E-07	upregulated
ARPC3P1	1.041052009	5.02869E-07	upregulated
LCP2	1.041653591	5.43318E-10	upregulated
RBMS1P1	1.041933674	1.33457E-05	upregulated
UBOX5.AS1	1.041978129	3.35888E-08	upregulated
TFEC	1.042225031	8.16895E-07	upregulated
ANKRD33	1.042280398	8.67132E-05	upregulated
LINC01473	1.043511711	4.13054E-06	upregulated
PLEKHD1	1.043898739	5.74777E-08	upregulated
LCK	1.044236614	1.68366E-06	upregulated
SMCO2	1.044269115	5.93488E-13	upregulated
TLR2	1.045265864	1.45774E-09	upregulated
SNORD46	1.045967099	1.17664E-09	upregulated
EVI2A	1.046064915	4.19112E-07	upregulated
RNASE4	1.046143099	2.48851E-11	upregulated
PABPC1P1	1.046278157	9.70499E-06	upregulated
TYMS	1.046337665	4.25597E-19	upregulated
ATP8B3	1.046565283	9.96337E-12	upregulated
TNFRSF11A	1.046998091	4.12055E-11	upregulated
CD96	1.047169499	1.58063E-09	upregulated
BMPR1B	1.047174896	4.06968E-05	upregulated
IL1RL1	1.04736382	2.00163E-06	upregulated
KDELR3	1.047460653	1.71088E-18	upregulated
FBXO6	1.048413762	6.75579E-15	upregulated
FAM72B	1.048593737	4.84785E-07	upregulated
RELL1	1.048602411	1.61935E-21	upregulated
HOXD3	1.048812307	2.11829E-09	upregulated
PIK3AP1	1.04889598	1.29864E-11	upregulated
FCGR2B	1.049526521	2.48153E-06	upregulated
TRPA1	1.049584692	3.59221E-05	upregulated

RADIL	1.049742865	5.33246E-07	upregulated
KLK10	1.049915054	0.000826259	upregulated
VSIG4	1.051050044	4.09623E-06	upregulated
TENT5B	1.051546748	1.44318E-08	upregulated
SULT1C2	1.051690926	1.59376E-05	upregulated
RN7SKP16	1.051758285	4.9591E-06	upregulated
APOL6	1.052012592	4.16911E-24	upregulated
KCND3	1.052145917	1.47498E-06	upregulated
IGKV2D.40	1.052772277	0.00313197	upregulated
PDIA2	1.053867975	0.000159593	upregulated
PIAS2	1.054152878	1.14511E-26	upregulated
MCUB	1.054401378	3.56888E-21	upregulated
GLIS3.AS1	1.05478821	0.000708848	upregulated
LILRB4	1.054851559	7.54159E-07	upregulated
C19orf38	1.055357569	7.23277E-09	upregulated
ZNF488	1.055755993	4.92174E-07	upregulated
IRX3	1.05605853	0.000312815	upregulated
HELB	1.056737941	1.17306E-17	upregulated
CHST5	1.05678945	0.000228557	upregulated
DIRC3	1.057178743	6.6029E-05	upregulated
B3GAT1	1.057379785	1.03715E-07	upregulated
PROX2	1.057464544	5.1895E-07	upregulated
AFAP1L2	1.058046709	4.4196E-15	upregulated
SIX3	1.058295058	0.000803747	upregulated
TOGARAM2	1.058361078	5.1014E-14	upregulated
MS4A14	1.059099161	1.00043E-09	upregulated
TMEM145	1.059289929	2.26979E-06	upregulated
PCDHGB3	1.059442694	5.28367E-08	upregulated
FRMD3.AS1	1.060184507	9.14411E-06	upregulated
MAEL	1.060801388	0.004068123	upregulated
ACRBP	1.061463475	5.67793E-14	upregulated
ARL17B	1.061700176	7.39166E-07	upregulated
SSTR5.AS1	1.062033217	0.001504429	upregulated
HAVCR2	1.062045097	1.6391E-09	upregulated
ARHGAP10	1.062688531	5.12411E-12	upregulated
MKNK1.AS1	1.064142208	1.74737E-09	upregulated
IZUMO1	1.064761388	2.78386E-08	upregulated
CLEC12A	1.065930798	7.30067E-06	upregulated
DUSP5P1	1.065940743	6.39483E-06	upregulated
MAP3K6	1.066155405	2.31601E-17	upregulated
SMG1P5	1.066664887	2.40844E-09	upregulated
DPYSL2	1.066665505	3.85739E-15	upregulated
GALNT5	1.067142499	3.16164E-13	upregulated
ANXA1	1.067968662	4.52213E-10	upregulated
PTAFR	1.068087228	1.90191E-09	upregulated
KCNJ15	1.068170291	9.45417E-06	upregulated
MSR1	1.068719015	5.70872E-06	upregulated
COX20	1.068872498	8.14064E-08	upregulated
POLR2J2	1.069242649	4.3021E-08	upregulated
SYNGR1	1.069263677	1.95181E-07	upregulated

CYP2C9	1.069328001	0.000131362	upregulated
CAPN9	1.070333733	0.00016817	upregulated
KCNIP2.AS1	1.070590118	1.48691E-10	upregulated
SLC7A2	1.070816775	6.82135E-06	upregulated
FGFR2	1.071664661	1.22732E-07	upregulated
BAAT	1.072815488	0.0008294	upregulated
CD300E	1.072855	8.40901E-06	upregulated
IGHV1.69.2	1.073161786	0.007402211	upregulated
DNER	1.073739433	4.34673E-05	upregulated
SNORA71B	1.073972997	5.64267E-07	upregulated
CR1	1.074301491	1.65512E-05	upregulated
SYT13	1.074638149	1.3656E-06	upregulated
RP1L1	1.075490018	1.76971E-07	upregulated
LUCAT1	1.07581544	5.56638E-05	upregulated
SIRPB1	1.075979255	2.41888E-06	upregulated
DIABLO	1.076292873	3.23084E-15	upregulated
MDM2	1.07757252	6.72088E-24	upregulated
FOXD4	1.077729369	5.07135E-10	upregulated
LINC01687	1.077846741	0.001113827	upregulated
RIMS3	1.078961013	4.91403E-09	upregulated
VNN3P	1.079305752	3.28889E-05	upregulated
FOXCUT	1.079918886	1.01299E-05	upregulated
PCDHGA4	1.079953762	3.33475E-08	upregulated
AJAP1	1.080544321	6.79799E-09	upregulated
BMAL2	1.08078016	1.13486E-21	upregulated
DOCK8.AS2	1.081011256	1.42207E-07	upregulated
CD209	1.081104635	6.40271E-08	upregulated
CCL13	1.08177303	0.000129171	upregulated
GCNT3	1.081777348	2.73501E-05	upregulated
NRIR	1.081897753	1.95633E-07	upregulated
ASRGL1	1.082327362	9.74051E-09	upregulated
LINC00943	1.083120646	3.34333E-06	upregulated
PARP14	1.084189759	1.0305E-23	upregulated
SLA2	1.084699794	6.21073E-13	upregulated
CA9	1.08518042	0.001408373	upregulated
OR5BA1P	1.0863405	6.48259E-07	upregulated
SNORA65	1.086780297	1.31004E-10	upregulated
PDGFRL	1.087038278	3.49511E-06	upregulated
ANXA2R	1.087206819	5.07036E-17	upregulated
H2AZP3	1.087220078	0.00015289	upregulated
STEAP3.AS1	1.087363248	5.11473E-09	upregulated
OSR2	1.087438398	3.23594E-09	upregulated
CD84	1.088244092	3.12144E-07	upregulated
PTPRG.AS1	1.088394428	2.09109E-05	upregulated
GVINP1	1.088982133	2.7935E-09	upregulated
GPRC5D	1.089112215	1.02238E-10	upregulated
MUC1	1.089393183	6.22829E-06	upregulated
BCL2A1	1.089431628	3.65941E-07	upregulated
SLC1A1	1.089469358	1.51335E-10	upregulated
PLPPR1	1.090088781	0.000316714	upregulated

ATP1B3.AS1	1.090294822	3.45873E-08	upregulated
GPR141	1.090423559	6.01499E-08	upregulated
ZCRB1P1	1.091095332	5.37619E-09	upregulated
NBEAL1	1.091478163	2.43908E-09	upregulated
PTMAP9	1.091599243	7.9953E-17	upregulated
PRSS23.AS1	1.091753665	5.26028E-09	upregulated
FCGR1CP	1.0918049	1.63029E-08	upregulated
PEG10	1.091976628	0.003562653	upregulated
NRTN	1.092965267	1.9702E-13	upregulated
NFATC1	1.093406436	4.32591E-11	upregulated
IQCM	1.094881364	0.00010878	upregulated
C8orf34.AS1	1.094883388	0.002151773	upregulated
BATF3	1.095621196	5.38666E-16	upregulated
HLA.DRB6	1.096016659	1.28822E-07	upregulated
KCNK1	1.09671819	5.02144E-14	upregulated
FPR1	1.097114952	7.54245E-06	upregulated
GFAP	1.098695433	5.25213E-06	upregulated
TOX	1.099298817	3.9299E-07	upregulated
LILRB3	1.09958841	1.04746E-10	upregulated
FYB1	1.100237847	5.6812E-09	upregulated
CXCR6	1.100609882	5.3482E-11	upregulated
FAM169A	1.100858988	8.04033E-09	upregulated
ABCC8	1.100908918	1.08005E-06	upregulated
IL10	1.100929432	2.17738E-08	upregulated
LINC02580	1.100987433	2.18851E-08	upregulated
CYLD.AS1	1.101458139	2.33293E-11	upregulated
UBE2D3P2	1.102669203	5.6812E-09	upregulated
ABCA15P	1.102780838	5.42081E-12	upregulated
IL12RB2	1.103619638	2.51043E-10	upregulated
LILRA6	1.103718304	1.58055E-08	upregulated
HMGN2P3	1.104865694	6.20669E-09	upregulated
IFI6	1.105435667	9.10762E-08	upregulated
TNRC6B	1.105990107	8.29342E-25	upregulated
ADGRG3	1.108240418	3.65941E-07	upregulated
SLC28A3	1.108754084	1.83292E-05	upregulated
BCAT1	1.108849846	5.56261E-07	upregulated
LY6E.DT	1.108962276	4.74847E-07	upregulated
LINC02405	1.109107615	1.51459E-06	upregulated
DOCK8.AS1	1.109153086	6.30696E-07	upregulated
HLA.DQB1	1.10974208	4.00259E-07	upregulated
SLC39A13.AS1	1.111175578	3.36673E-09	upregulated
SLC47A2	1.111297054	3.21274E-11	upregulated
FBXL13	1.111448037	6.98919E-11	upregulated
APOL2	1.111952913	1.51556E-21	upregulated
CLEC7A	1.114194879	4.88832E-09	upregulated
APOBEC3H	1.114370628	2.9793E-12	upregulated
ANK1	1.114449202	1.92506E-07	upregulated
C1QB	1.114788108	3.29339E-09	upregulated
RN7SL481P	1.115395525	1.65562E-08	upregulated
IKBKE.AS1	1.11540847	2.5898E-10	upregulated

RN7SL368P	1.115789577	8.05893E-08	upregulated
SNORD83A	1.116568185	8.31578E-10	upregulated
TDRD1	1.117146433	0.002140564	upregulated
FFAR2	1.117196809	2.87334E-06	upregulated
FCGR1A	1.117707506	3.31284E-09	upregulated
LINC00630	1.117838324	3.60191E-11	upregulated
SLAMF8	1.118133386	5.61272E-08	upregulated
PRC1.AS1	1.118174804	2.8032E-10	upregulated
PTMAP5	1.118213599	4.3982E-16	upregulated
KLHL11	1.120472176	4.20534E-19	upregulated
UBE2L6	1.120684842	1.30746E-16	upregulated
CXCL11	1.122188024	2.70422E-05	upregulated
NARF.IT1	1.125833252	7.06352E-13	upregulated
FKBP9P1	1.12583664	2.78742E-10	upregulated
ZMYND15	1.125870237	2.63083E-20	upregulated
SLC25A21	1.12720118	1.83503E-05	upregulated
GNAT2	1.127897173	9.94552E-13	upregulated
ANO2	1.128212747	6.02006E-14	upregulated
FXYD4	1.129784479	5.52693E-06	upregulated
CALM2P2	1.131045161	2.27137E-09	upregulated
TJP2	1.131096201	5.8062E-11	upregulated
IL19	1.133095624	7.871E-06	upregulated
NUTM2B	1.133523392	5.62634E-10	upregulated
FCGR1BP	1.135894459	1.04064E-10	upregulated
TNC	1.136210057	1.19355E-06	upregulated
SYNPO2L	1.136399186	4.12413E-08	upregulated
LNCTAM34A	1.136529258	2.6771E-13	upregulated
RPL17P50	1.136673129	5.9413E-23	upregulated
LINC02884	1.137378262	7.56025E-09	upregulated
ADRB1	1.137599477	4.79135E-07	upregulated
RAB38	1.140220294	2.08623E-10	upregulated
MICB	1.140944924	2.87399E-14	upregulated
HSALR1	1.1409827	7.56025E-09	upregulated
CCDC200	1.141290033	2.34398E-13	upregulated
APOL4	1.141529265	2.06229E-11	upregulated
TRG.AS1	1.14193907	1.73833E-12	upregulated
TMEM231P1	1.142573824	2.84038E-08	upregulated
ADAM28	1.142640668	4.08204E-10	upregulated
C3orf49	1.143632651	7.85577E-10	upregulated
IL2RA	1.145551563	5.80736E-10	upregulated
RPS15AP10	1.145616185	3.85849E-10	upregulated
ANKRD36BP2	1.146764589	2.02238E-05	upregulated
BEAN1	1.147228823	1.08529E-06	upregulated
RPS12P26	1.148495057	1.45751E-05	upregulated
TLE6	1.149334659	1.39667E-11	upregulated
SLC31A2	1.149524257	1.68454E-09	upregulated
ALPK2	1.149577974	1.38576E-09	upregulated
SPHK1	1.149606566	4.71759E-09	upregulated
FDCSP	1.149995907	0.000997343	upregulated
CMTM2	1.151252675	1.86047E-12	upregulated

SNORA5A	1.151845052	8.57507E-07	upregulated
HSPA8P15	1.152956487	4.75531E-08	upregulated
TAS2R4	1.153230768	1.28538E-07	upregulated
C11orf65	1.15343843	9.33993E-13	upregulated
RPL4P6	1.153584189	1.56265E-17	upregulated
S100A9	1.153926683	1.13768E-06	upregulated
SAP30L.AS1	1.154069976	1.6252E-11	upregulated
SHF	1.154097741	3.10698E-08	upregulated
LINC01117	1.154889926	1.95958E-08	upregulated
ST6GALNAC6	1.155889277	1.68023E-12	upregulated
LINC00299	1.157100211	9.7471E-11	upregulated
VIM.AS1	1.157534341	7.23842E-14	upregulated
AQP9	1.157614983	2.61584E-05	upregulated
C8orf74	1.158203873	1.11955E-05	upregulated
LINC00944	1.158671657	1.43485E-07	upregulated
SLFN12L	1.158888805	2.56656E-11	upregulated
GPR3	1.160669967	9.17206E-18	upregulated
SUPT16HP1	1.161422441	6.98878E-13	upregulated
LINC01750	1.161653129	2.49971E-08	upregulated
ACAP2.IT1	1.161710316	9.50046E-06	upregulated
FER1L5	1.162519332	9.37183E-08	upregulated
RNF125	1.162664588	4.70514E-15	upregulated
CBLIF	1.16295495	0.000275469	upregulated
RETREG1.AS1	1.163094893	5.9873E-09	upregulated
SNORA3B	1.163296369	1.13226E-09	upregulated
TRBV27	1.163528716	4.96121E-09	upregulated
SLC30A4.AS1	1.165504437	2.97703E-11	upregulated
CCL4L2	1.165929903	3.52673E-09	upregulated
VWA3B	1.166869489	1.84504E-11	upregulated
AADAC	1.167277172	1.21815E-05	upregulated
PCDHGA5	1.168450949	1.73137E-09	upregulated
ADGRL1.AS1	1.16938217	4.47451E-18	upregulated
CD44.AS1	1.169649002	1.91244E-08	upregulated
ARRDC3.AS1	1.169960197	2.72371E-12	upregulated
IL2RB	1.170109937	4.98132E-12	upregulated
MMP1	1.170391155	0.000122972	upregulated
WNT8B	1.170888156	1.35645E-10	upregulated
RAB3A	1.171728572	3.72992E-16	upregulated
LINC01811	1.171781396	2.56334E-05	upregulated
RPS3AP34	1.172697628	8.82995E-09	upregulated
CD226	1.173309041	1.26834E-10	upregulated
HLA.DQA1	1.173400243	1.74918E-08	upregulated
HSPA5P1	1.173657976	1.72753E-16	upregulated
TANC2	1.174029149	2.81535E-11	upregulated
TIAM1.AS1	1.174090821	3.66019E-06	upregulated
CFAP57	1.174315229	4.27355E-10	upregulated
CARMN	1.175061074	1.71599E-06	upregulated
KLK6	1.175138969	0.00105925	upregulated
ADGRE1	1.17593717	5.49664E-10	upregulated
CLNK	1.177322894	1.18457E-12	upregulated

TIMM23B.AGA	1.177744157	2.1447E-09	upregulated
MMP25.AS1	1.178104702	3.92896E-14	upregulated
CHRM1	1.178575448	2.53803E-06	upregulated
ZNF695	1.179830793	6.34351E-15	upregulated
USP18	1.180742382	5.54498E-17	upregulated
FRAS1	1.182149324	2.65759E-07	upregulated
RAET1K	1.182841257	9.46588E-13	upregulated
RAC3	1.182865673	1.33094E-13	upregulated
TPRXL	1.182958103	0.000295439	upregulated
HCN2	1.183522288	1.37624E-08	upregulated
KLK11	1.183693106	0.000162194	upregulated
TRBV6.5	1.184249241	1.68065E-09	upregulated
ARHGEF4	1.185622864	3.44242E-07	upregulated
ST3GAL3	1.185646322	7.20097E-11	upregulated
HRH2	1.186097253	6.20521E-10	upregulated
GRIK1	1.187060607	9.14411E-06	upregulated
AGAP2	1.187116086	1.13375E-10	upregulated
SIX4	1.187457919	5.85943E-09	upregulated
FCGR2C	1.187802815	2.10519E-07	upregulated
ENPP7P4	1.18831131	6.08784E-07	upregulated
SULT1C5P	1.188617673	1.07705E-06	upregulated
XCL2	1.189636633	2.1593E-10	upregulated
PRF1	1.191056651	3.5702E-08	upregulated
PTGER2	1.191172537	7.29389E-09	upregulated
LIPN	1.191843352	7.41249E-05	upregulated
RPL15P18	1.191848613	2.66572E-07	upregulated
VTCN1	1.192098154	7.11648E-05	upregulated
GZMK	1.192179249	7.39394E-08	upregulated
HLA.DPB1	1.19243007	2.80432E-11	upregulated
PIGHP1	1.192446413	8.02714E-17	upregulated
RPS27AP12	1.193850437	9.69284E-11	upregulated
CALB2	1.194908454	6.05068E-05	upregulated
LINC01876	1.195113852	7.81136E-07	upregulated
PLEK	1.19548145	2.7936E-08	upregulated
NBAT1	1.195568335	0.000798019	upregulated
JAK2	1.196452398	3.48805E-27	upregulated
ROBO3	1.196682561	2.11947E-16	upregulated
PDCD1	1.197416351	1.92329E-11	upregulated
CEACAM3	1.197637987	2.32208E-10	upregulated
SLC2A5	1.198117649	4.09839E-11	upregulated
LINC02474	1.198585106	0.000963038	upregulated
CXCR4	1.200787832	1.81338E-12	upregulated
DPYD	1.201304449	3.4239E-10	upregulated
TSSK3	1.201518823	5.60188E-11	upregulated
SLC28A2.AS1	1.202591072	7.05251E-11	upregulated
ICAM5	1.205311374	3.44711E-09	upregulated
CNFN	1.205961492	7.1909E-11	upregulated
ZNF618	1.206025371	2.11269E-14	upregulated
IL9R	1.207513395	2.67269E-14	upregulated
SLC34A3	1.207595338	5.40112E-08	upregulated

RPL21P80	1.208304658	9.76563E-13	upregulated
REG1B	1.208724853	0.046042204	upregulated
RHOF	1.208963051	6.45967E-13	upregulated
KCNJ3	1.209050014	5.72391E-05	upregulated
ERFL	1.209877673	3.91262E-13	upregulated
LINC02970	1.210234162	1.68687E-10	upregulated
DNAH11	1.210421174	2.39261E-08	upregulated
ISYNA1	1.210506904	4.27211E-11	upregulated
TCAF2	1.211750395	2.80024E-19	upregulated
ERVH48.1	1.212233423	3.19092E-06	upregulated
KLHDC7B	1.213218488	2.98167E-12	upregulated
SPDYE1	1.213558479	1.21877E-06	upregulated
MGAM	1.213620447	1.0543E-08	upregulated
SYCE3	1.213700572	4.60969E-12	upregulated
HAPLN3	1.214473861	9.82487E-14	upregulated
ZNF45.AS1	1.215287203	1.45121E-07	upregulated
TRDC	1.215349557	1.66915E-06	upregulated
POLR2J3.1	1.215794132	6.75224E-09	upregulated
UICLM	1.215942607	0.000142172	upregulated
GUSBP18	1.216557295	1.7388E-10	upregulated
ILK	1.216823296	4.2508E-14	upregulated
SMG1P6	1.217153101	6.89614E-09	upregulated
SDCBP2.AS1	1.217167112	4.33502E-11	upregulated
ARC	1.217693732	5.81066E-07	upregulated
MRPS35.DT	1.219705057	4.33392E-05	upregulated
TENT5A	1.219726121	5.9169E-22	upregulated
RHOA.IT1	1.220760714	1.25947E-11	upregulated
ENTPD1.AS1	1.22193018	3.36646E-13	upregulated
CCNB3	1.222155897	4.84316E-13	upregulated
ITIH6	1.222385522	4.88609E-06	upregulated
KCNQ2	1.222392191	0.001140685	upregulated
HLA.DMB	1.222590758	7.84185E-13	upregulated
TYMP	1.222873451	4.64278E-15	upregulated
IRX2	1.222882989	0.000423573	upregulated
NPAS3	1.224139755	1.73278E-08	upregulated
TIAM1	1.226176094	5.70491E-11	upregulated
LINC01967	1.226843454	4.52732E-05	upregulated
GRIA3	1.22717306	5.80951E-08	upregulated
STS	1.227333381	5.95183E-21	upregulated
FTH1P22	1.22838797	2.05711E-13	upregulated
PADI4	1.228410155	2.31164E-07	upregulated
FCGR2A	1.228507022	7.22393E-12	upregulated
CLEC5A	1.22922455	4.9688E-08	upregulated
DYNLT4	1.229812284	7.0592E-13	upregulated
HYPK	1.230388453	2.45597E-15	upregulated
LINC00472	1.230830678	6.32968E-06	upregulated
MT2A	1.232054681	1.58154E-13	upregulated
IFIT2	1.233919733	1.86123E-15	upregulated
GRIP2	1.235248748	1.3858E-07	upregulated
MFSD13B	1.238233571	2.6845E-07	upregulated

NCCRP1	1.239085929	0.000113936	upregulated
CLEC4E	1.241297733	7.39394E-08	upregulated
ACKR4	1.241562764	1.98279E-08	upregulated
DPY19L2	1.242417002	3.22366E-09	upregulated
GPR27	1.24243445	5.41457E-07	upregulated
AKAP3	1.243183872	7.34969E-14	upregulated
ASB14	1.244245978	1.33794E-14	upregulated
CIST1	1.244738236	5.9666E-05	upregulated
CPB2.AS1	1.245635269	4.6982E-05	upregulated
MRC1	1.24678179	3.3148E-07	upregulated
MT1L	1.24694181	6.17112E-11	upregulated
ZNF571.AS1	1.247508941	2.98278E-08	upregulated
CLEC6A	1.248316761	7.49362E-08	upregulated
APOBEC3G	1.248667569	3.03159E-16	upregulated
NSA2P7	1.249018281	4.08193E-10	upregulated
IFIT3	1.249224938	1.68065E-14	upregulated
MLPH	1.249262297	5.29267E-09	upregulated
PCDHGB1	1.249396322	1.00605E-06	upregulated
MICE	1.250694103	1.63953E-13	upregulated
CD74	1.250823073	7.45918E-14	upregulated
XAF1	1.251337326	2.12905E-13	upregulated
IGHV3.13	1.251457531	0.000103696	upregulated
FSCN1	1.251523028	4.58649E-10	upregulated
GSEC	1.252696097	3.79548E-15	upregulated
RPS2P53	1.253028266	6.17871E-10	upregulated
DOCK5	1.254455752	5.62428E-29	upregulated
TCP11L2	1.254626809	8.00416E-22	upregulated
KRT1	1.25776027	1.37717E-06	upregulated
CCL8	1.258057243	1.76066E-07	upregulated
LINC01871	1.258196427	9.1296E-13	upregulated
ABCG5	1.258994942	1.48884E-08	upregulated
PAX9	1.259894735	1.51898E-08	upregulated
TCN1	1.260809259	0.003441312	upregulated
RAP1GAP	1.261195553	5.49664E-10	upregulated
GPR174	1.265653498	9.24547E-10	upregulated
CXCL17	1.265825215	0.000406774	upregulated
TRGC1	1.26599735	4.41099E-14	upregulated
RAB26	1.267012733	1.86821E-06	upregulated
CEACAM4	1.267676732	7.5966E-12	upregulated
CSF3R	1.268338234	1.55268E-08	upregulated
RAD17P1	1.269131272	8.4614E-09	upregulated
PLCL2	1.269267196	1.23571E-14	upregulated
GP6	1.271846972	3.73008E-11	upregulated
APOL1	1.272188701	4.25895E-13	upregulated
CALHM6	1.272884449	5.94227E-08	upregulated
SLC7A11	1.272930786	8.50132E-17	upregulated
RNF152	1.272938373	2.66817E-15	upregulated
TFF1	1.273658924	0.000150758	upregulated
IGFL2	1.274950072	2.38362E-05	upregulated
KLRK1	1.275266625	1.97011E-12	upregulated

THBS1.IT1	1.27535776	8.62658E-06	upregulated
TLL2	1.275394814	2.92917E-13	upregulated
OXCT1	1.275766381	5.5451E-19	upregulated
CCL7	1.276215044	3.16064E-08	upregulated
ADAMTS9.AS1	1.276528857	4.63521E-06	upregulated
RGL4	1.27741495	5.9476E-17	upregulated
PDCD1LG2	1.278068955	4.84597E-11	upregulated
MYRF	1.278437309	2.61697E-10	upregulated
LRRC26	1.280633919	0.000932356	upregulated
GCNT7	1.281202872	1.84141E-07	upregulated
KCNN2	1.281911469	7.9515E-14	upregulated
HDHD2	1.281926	3.48313E-14	upregulated
DLEU2L	1.282073996	1.55989E-07	upregulated
SMG1P3	1.282338782	2.51615E-12	upregulated
RPL11P3	1.283028991	0.002797056	upregulated
NPW	1.283220668	8.75256E-06	upregulated
TENM3.AS1	1.284375413	3.32318E-07	upregulated
TVP23C	1.28462682	1.50412E-15	upregulated
ST6GALNAC2	1.287379289	1.00263E-11	upregulated
KLC1.AS1	1.287948152	1.34093E-15	upregulated
LINC01550	1.289561171	1.07096E-07	upregulated
SLC4A4	1.289860383	4.01512E-05	upregulated
KRT6B	1.290582702	0.000590606	upregulated
HYMAI	1.290606549	2.86975E-06	upregulated
SLC16A6P1	1.290809889	7.26402E-11	upregulated
LINC01684	1.291891574	2.69477E-08	upregulated
CXCL8	1.292006775	2.87383E-05	upregulated
LINC01191	1.293528427	1.26299E-11	upregulated
MMP12	1.294716613	1.1034E-07	upregulated
FAM186A	1.294994798	3.1669E-13	upregulated
HELLPAR	1.295109795	7.56386E-07	upregulated
PRH2	1.295384511	2.51101E-05	upregulated
CEP85L	1.297770609	7.84551E-17	upregulated
CATSPERB	1.300363667	4.79155E-14	upregulated
PRSS27	1.301101022	1.30207E-14	upregulated
ARHGEF10	1.302158182	4.44126E-11	upregulated
SMIM35	1.30222297	2.93619E-17	upregulated
HOXB.AS2	1.302412529	1.9031E-08	upregulated
DPF1	1.302532299	2.65401E-15	upregulated
PP1P	1.303470943	3.78474E-07	upregulated
CERNA1	1.303550373	7.31358E-13	upregulated
FAR2P1	1.303941426	0.000113778	upregulated
PCDHB13	1.304128747	7.30134E-12	upregulated
NFIA	1.305127935	2.18588E-16	upregulated
HLA.DRB1	1.305265094	3.81827E-12	upregulated
ANO1	1.305924054	9.44001E-12	upregulated
MYRFL	1.306141897	2.83277E-06	upregulated
NFIA.AS2	1.307325688	3.32771E-06	upregulated
POTEF	1.309271726	6.30993E-08	upregulated
CCNP	1.309587476	2.11898E-10	upregulated

PTMAP12	1.309886885	4.16308E-10	upregulated
LAIR2	1.31020688	6.3852E-09	upregulated
HPR	1.311607435	2.85488E-05	upregulated
OAS2	1.315104522	1.04239E-11	upregulated
DARS1.AS1	1.31527802	1.27375E-20	upregulated
LINC01934	1.315404932	2.57341E-08	upregulated
RNF144B	1.319765494	5.23417E-25	upregulated
KYNU	1.321946147	2.93958E-12	upregulated
RNVU1.1	1.325049721	3.56571E-11	upregulated
APRG1	1.325637907	1.09027E-12	upregulated
RPL7AP10	1.327483627	3.57046E-13	upregulated
IGHV1.69	1.328449749	8.5647E-05	upregulated
FAM83A	1.329147091	2.76817E-05	upregulated
IL18RAP	1.329384384	5.39202E-16	upregulated
SRD5A3.AS1	1.329824677	4.09111E-24	upregulated
MTNR1A	1.330542372	7.1909E-11	upregulated
HLA.DRA	1.332924238	5.21469E-12	upregulated
TP73	1.333593532	4.57593E-14	upregulated
IL1RN	1.335156887	6.3852E-09	upregulated
SLFNL1	1.336263142	2.59441E-15	upregulated
ENO2	1.33679698	8.64495E-21	upregulated
RDH16	1.33824306	1.68023E-12	upregulated
RAET1E	1.339320347	9.04479E-14	upregulated
CASKIN1	1.33953822	1.35205E-08	upregulated
ARG1	1.341127858	7.93963E-11	upregulated
CSNK1G2.AS1	1.342198105	2.41546E-10	upregulated
CARINH	1.342846756	3.80727E-22	upregulated
HIF1A.AS3	1.342909733	1.43082E-05	upregulated
TMPRSS3	1.342972838	2.80992E-10	upregulated
SDR16C5	1.343261291	1.69981E-06	upregulated
PLAAT1	1.343781135	1.11708E-06	upregulated
AIF1L	1.346257574	8.22934E-15	upregulated
NAIP	1.347080029	4.14956E-16	upregulated
CASP1P2	1.347462334	2.35561E-09	upregulated
PFDN1P1	1.349755139	4.50322E-13	upregulated
PPP4R2P1	1.34976682	8.85179E-12	upregulated
CLMAT3	1.350206206	7.3011E-08	upregulated
BHMT	1.350447091	2.20079E-05	upregulated
APOBEC3A	1.351050953	9.29322E-11	upregulated
LANCL1.AS1	1.351475228	3.44846E-10	upregulated
MESTIT1	1.352206541	1.03875E-11	upregulated
LINC02605	1.353365706	6.30505E-12	upregulated
LINC01088	1.353818987	1.69625E-06	upregulated
CYP19A1	1.355383166	2.02201E-09	upregulated
SLC6A16	1.355397113	8.30447E-13	upregulated
LINC02475	1.35555362	2.61737E-05	upregulated
MT2P1	1.356402262	2.3119E-11	upregulated
IFI44L	1.356962699	2.78145E-08	upregulated
LINGO1	1.357179717	9.97238E-13	upregulated
CXCR1	1.357347918	2.70081E-07	upregulated

TRBV9	1.35784566	1.82253E-12	upregulated
UST	1.35855425	5.39745E-11	upregulated
WASIR2	1.358841774	1.04359E-11	upregulated
ONECUT2	1.358989828	1.44327E-09	upregulated
FFAR4	1.360074702	3.38148E-07	upregulated
CCL4	1.360577643	9.7973E-14	upregulated
SLC43A3	1.360767843	7.6217E-14	upregulated
WARS1	1.360912114	1.14438E-29	upregulated
SNX18P3	1.362153631	2.22899E-14	upregulated
SOX8	1.364875495	3.81241E-06	upregulated
SERPINB5	1.366000888	4.6936E-08	upregulated
HES7	1.366574804	9.27803E-09	upregulated
PTGS2	1.366825084	6.83888E-07	upregulated
SEMA6A.AS1	1.367163066	4.84815E-14	upregulated
USP30.AS1	1.367921116	1.22046E-15	upregulated
RPL3P1	1.368814594	3.16183E-10	upregulated
TGFBR3L	1.368907434	9.12731E-11	upregulated
CCDC68	1.369099657	8.35237E-20	upregulated
AURKC	1.369383952	2.67759E-18	upregulated
BARX2	1.369635207	2.00847E-08	upregulated
XIRP1	1.370618961	5.38941E-10	upregulated
LINC02099	1.371215583	1.57811E-11	upregulated
TSIX	1.37227778	2.18244E-07	upregulated
GRAMD1B	1.372544846	2.85239E-11	upregulated
CDH15	1.374374635	9.74876E-14	upregulated
PRODH	1.37483884	5.75038E-08	upregulated
CC2D2B	1.375294548	7.60943E-10	upregulated
LRRC4	1.376024289	1.80089E-09	upregulated
GFI1B	1.376906384	2.16483E-09	upregulated
ZNF564	1.377352179	4.66404E-16	upregulated
IFNG.AS1	1.377379065	6.47394E-08	upregulated
ABCG8	1.37846777	3.14747E-08	upregulated
TMEM40	1.379238977	1.36779E-07	upregulated
TP53AIP1	1.381048326	4.41418E-12	upregulated
HLA.DQB1.AS1	1.381682211	1.79578E-12	upregulated
CD8A	1.381807487	2.46165E-12	upregulated
HLA.DPA1	1.38231722	3.34547E-12	upregulated
GBP1	1.382646314	2.20928E-20	upregulated
ANKRD36	1.383702306	3.78954E-13	upregulated
HSPA2.AS1	1.384091623	1.05428E-10	upregulated
BST2	1.384659935	3.11494E-10	upregulated
COPG2IT1	1.385116693	2.45385E-12	upregulated
PLA2G4A	1.385435813	2.19718E-08	upregulated
SLC18A1	1.386229249	4.05306E-05	upregulated
FAM72C	1.387651833	1.56655E-14	upregulated
COX7CP2	1.388819532	2.41519E-21	upregulated
HTR2B	1.38975893	4.87751E-07	upregulated
SMC1B	1.396221941	4.47608E-09	upregulated
LRRC73	1.396266405	2.60797E-15	upregulated
TMEM151A	1.398462451	2.17572E-08	upregulated

ANXA2P1	1.402238709	6.42589E-18	upregulated
PKD1L3	1.404525616	3.27241E-09	upregulated
PLA2G3	1.40452865	1.80277E-05	upregulated
GLT1D1	1.405877597	1.4561E-13	upregulated
FCGR3B	1.407118724	3.37176E-07	upregulated
PTMAP11	1.407460215	1.42258E-15	upregulated
KIAA1671.AS1	1.408920707	7.35505E-11	upregulated
RPL22L1	1.410665887	4.35719E-17	upregulated
LINC01359	1.410808965	6.96784E-09	upregulated
ZNF451.AS1	1.411579373	2.59159E-10	upregulated
FCGR3A	1.413943335	9.90146E-11	upregulated
ETV5	1.41456086	3.57995E-21	upregulated
AVPR1A	1.415031961	1.05373E-11	upregulated
LINC02656	1.415277665	2.97556E-09	upregulated
PDGFRL2P	1.415665893	1.47331E-11	upregulated
SHOC1	1.417116699	5.30748E-05	upregulated
FAM13A.AS1	1.417463645	1.76623E-14	upregulated
HNRNPA1P49	1.417887295	1.85139E-13	upregulated
IL21	1.419593029	6.04414E-10	upregulated
SP140	1.41984311	1.30465E-15	upregulated
MAK	1.420710141	1.16975E-08	upregulated
MAD1L1	1.421444926	1.07655E-13	upregulated
ESR2	1.421798723	4.71713E-21	upregulated
RN7SL381P	1.422914536	3.04292E-11	upregulated
SAXO3	1.423442434	6.13773E-14	upregulated
RASGRP1	1.423966433	2.01448E-17	upregulated
SAMD9L	1.424054227	1.83429E-18	upregulated
HPSE	1.425403009	6.61915E-30	upregulated
FAHD2P1	1.427127644	7.7734E-11	upregulated
PLAAT5	1.428341058	5.31009E-07	upregulated
ENTREP1	1.429210035	3.46722E-14	upregulated
LINC01426	1.429536632	5.6079E-12	upregulated
PGLYRP3	1.43054381	6.91578E-07	upregulated
SRGAP2D	1.430871956	3.09651E-15	upregulated
EP300.AS1	1.430902832	7.19981E-20	upregulated
LINC03076	1.43201792	4.66643E-09	upregulated
DMBT1	1.433781176	0.000253626	upregulated
SNORD94	1.434398267	3.77231E-10	upregulated
CALB1	1.434499518	0.003920781	upregulated
IL1R2	1.436037928	8.54905E-11	upregulated
CBFA2T3	1.436765825	5.2668E-08	upregulated
HTR3A	1.43679406	4.99016E-09	upregulated
ACAD9.DT	1.437022103	5.68708E-15	upregulated
ZIC2	1.437195116	4.85229E-05	upregulated
FCAR	1.437281064	2.28136E-08	upregulated
APOL3	1.439464837	3.87975E-22	upregulated
WNT7B	1.441113295	1.97626E-06	upregulated
CXCR2P1	1.442518478	4.20599E-11	upregulated
LINC00261	1.443327809	3.35516E-06	upregulated
SCN2A	1.445456875	9.45582E-07	upregulated

NDUFA4L2	1.448698424	1.08724E-12	upregulated
PPP1R3B.DT	1.449622014	4.3859E-13	upregulated
CRYBA2	1.450522907	5.59582E-08	upregulated
VEPH1	1.450735293	1.29766E-12	upregulated
LINC00939	1.454490053	3.72246E-08	upregulated
SLC5A4	1.455836739	1.13824E-14	upregulated
HMGN1P1	1.456935766	8.6311E-16	upregulated
AMANZI	1.458194499	1.46604E-06	upregulated
FER1L6	1.458396628	8.95293E-06	upregulated
TRGV10	1.459529743	1.87315E-15	upregulated
VPS39.DT	1.460631305	9.46646E-12	upregulated
LRIG2.DT	1.461787307	7.99207E-12	upregulated
PCDHB8	1.463492568	3.14373E-07	upregulated
MT.TF	1.46728206	2.65153E-05	upregulated
HOXD1	1.468188508	1.22705E-12	upregulated
RPL37P12	1.470044083	6.20222E-10	upregulated
SLC1A3	1.47077343	3.26667E-11	upregulated
MAMLD1	1.470947945	2.51034E-15	upregulated
FTH1P4	1.471600754	3.47251E-14	upregulated
DNAJC8P2	1.473091034	7.29955E-15	upregulated
PRDM4.AS1	1.473323715	3.79643E-15	upregulated
EOMES	1.473529434	1.73239E-12	upregulated
ALDH1L1	1.473965982	9.20615E-06	upregulated
RTN4RL2	1.474005966	1.99143E-19	upregulated
RIMBP3	1.474617666	2.24277E-19	upregulated
LYZ	1.478287177	1.32329E-07	upregulated
PELO.AS1	1.478471307	3.86526E-21	upregulated
CAMK2A	1.479011979	1.71998E-13	upregulated
IGFL3	1.479912024	1.95171E-06	upregulated
**OR2I1P**	1.480615061	2.94024E-09	upregulated
LOXL1.AS1	1.481753309	5.84013E-19	upregulated
NOTCH2NLC	1.481817561	3.16647E-07	upregulated
SNORA66	1.482520959	3.02028E-16	upregulated
HLA.DRB5	1.483436399	3.79588E-10	upregulated
TRGC2	1.485881894	1.83816E-17	upregulated
PRH1	1.487921776	2.81947E-09	upregulated
ABCA3	1.487960971	9.64068E-11	upregulated
CXCL13	1.488099498	2.14061E-08	upregulated
NPSR1.AS1	1.492193159	9.61255E-08	upregulated
LINC02084	1.493200295	2.53959E-14	upregulated
MIAT	1.49385365	1.58497E-13	upregulated
MTCO2P12	1.495024723	5.84402E-07	upregulated
PLLP	1.495866695	7.38086E-21	upregulated
CYP2U1.AS1	1.496645939	8.03371E-14	upregulated
ARMC3	1.497480329	5.02821E-08	upregulated
ATOH1	1.499227637	1.94045E-06	upregulated
RN7SL834P	1.500221145	5.03275E-20	upregulated
AMZ1	1.504505649	4.17139E-14	upregulated
SPDEF	1.505223411	2.63248E-07	upregulated
PNMA2	1.506138484	9.7471E-11	upregulated

MAP3K5.AS2	1.506350618	7.07688E-13	upregulated
UBASH3B	1.507076431	1.33802E-20	upregulated
CD163	1.507220594	2.97903E-10	upregulated
DEFA6	1.508322047	0.001881149	upregulated
EDRF1.AS1	1.508982795	5.51603E-12	upregulated
CD164L2	1.509613472	2.46384E-14	upregulated
CHRM3.AS2	1.51119137	3.83462E-07	upregulated
FGFBP2	1.51545195	4.01895E-16	upregulated
MUC4	1.516142972	1.01173E-07	upregulated
LINC02041	1.517212032	7.29009E-13	upregulated
HERC3	1.519030994	1.01475E-16	upregulated
TLR8	1.520324366	3.96419E-12	upregulated
PROK2	1.520370158	3.48475E-07	upregulated
PLK2	1.522156873	6.81146E-28	upregulated
HOXC11	1.523860821	1.49078E-05	upregulated
ASPHD2	1.525223387	4.71847E-29	upregulated
MT1E	1.528647902	2.03437E-11	upregulated
DTHD1	1.529772641	9.12731E-11	upregulated
GTF3C2.AS1	1.533811098	9.3621E-12	upregulated
ZBED2	1.53405905	6.68541E-11	upregulated
LINC01962	1.534621418	2.37096E-22	upregulated
RPS27P29	1.536322943	7.56544E-10	upregulated
HSPA4L	1.536391726	1.07703E-15	upregulated
HLA.DMA	1.537344518	5.13345E-22	upregulated
CRTAM	1.539408487	1.63102E-15	upregulated
MT.ND5	1.540186594	1.20241E-15	upregulated
LINC02939	1.542447176	6.93223E-11	upregulated
LINC03033	1.545042092	7.37726E-16	upregulated
CCL5	1.5457858	6.60629E-17	upregulated
ANKRD36B	1.545912194	4.20747E-19	upregulated
INHBA.AS1	1.546439869	1.81633E-08	upregulated
RPS3AP38	1.546786954	1.56054E-10	upregulated
MUC16	1.548854174	0.000101745	upregulated
RNA5SP37	1.550211304	3.62427E-14	upregulated
TRIB2	1.551353235	3.41292E-27	upregulated
BIRC3	1.553884448	1.99075E-22	upregulated
FBXO39	1.558506306	6.3152E-17	upregulated
GAD1	1.558776391	1.46884E-10	upregulated
FCRL6	1.559088353	1.5983E-17	upregulated
ECE1.AS1	1.559405704	7.11715E-15	upregulated
TBX21	1.56030526	1.27375E-20	upregulated
SYCP3	1.562381222	3.51866E-10	upregulated
PLA2G2A	1.562570149	1.21815E-05	upregulated
BACH1.IT1	1.563239085	2.95434E-11	upregulated
HMGA2.AS1	1.564633486	9.99816E-09	upregulated
PTPRU	1.564655275	4.44347E-13	upregulated
H3C10	1.565698304	3.69579E-14	upregulated
AGBL4	1.56646359	7.65855E-13	upregulated
HOXC4	1.567046309	7.53144E-12	upregulated
HLA.DPB2	1.568239134	4.43028E-14	upregulated

PRDM16	1.571407089	2.53344E-12	upregulated
TPO	1.572596508	3.11987E-14	upregulated
DAPK1	1.57296853	3.23345E-16	upregulated
CCL18	1.577163497	1.14707E-07	upregulated
LACTB2.AS1	1.577975318	8.82956E-14	upregulated
PRKCG	1.581094114	4.09702E-08	upregulated
KHDC1	1.582527816	5.22616E-14	upregulated
LRRN3	1.582751111	3.69981E-08	upregulated
ZNF114	1.582836471	6.02006E-14	upregulated
GSDMC	1.584345859	4.55161E-21	upregulated
LYG1	1.585356783	5.00687E-30	upregulated
GATA4	1.586101539	5.32845E-05	upregulated
FBXL16	1.587442674	2.27715E-15	upregulated
CHST4	1.590835451	9.5862E-06	upregulated
LINC02688	1.590876819	1.00602E-14	upregulated
SPINK4	1.592006224	0.000423504	upregulated
KCNE1	1.592492154	3.47251E-14	upregulated
NKG7	1.593040141	2.21051E-17	upregulated
ARAP1.AS2	1.593404525	1.15742E-11	upregulated
COL6A6	1.593573684	1.81189E-10	upregulated
SMIM33	1.597009614	3.88108E-10	upregulated
CD55	1.597480785	6.87601E-25	upregulated
ADGRF1	1.598812662	7.45267E-09	upregulated
LYPD5	1.599884699	1.67963E-24	upregulated
TINCR	1.599973619	1.57564E-10	upregulated
CD244	1.600827588	5.28665E-18	upregulated
TAS2R15P	1.603787634	3.30981E-12	upregulated
SECTM1	1.604193916	9.58733E-17	upregulated
RPL36AP30	1.604694263	5.26431E-17	upregulated
MIRLET7BHG	1.605013529	1.47622E-23	upregulated
MEFV	1.605024994	5.3716E-15	upregulated
EIF4EP1	1.605092758	2.77939E-10	upregulated
RN7SKP23	1.60709535	4.71695E-15	upregulated
TPM1.AS	1.60869864	1.31952E-11	upregulated
SH2D1B	1.609017509	1.34266E-21	upregulated
GJA3	1.609156486	1.49734E-10	upregulated
MIR924HG	1.610540698	2.00794E-16	upregulated
CREB3L1	1.610607314	4.73639E-15	upregulated
GLYCTK.AS1	1.613710331	1.76904E-14	upregulated
ITIH2	1.614258449	4.62315E-13	upregulated
SNORA14B	1.614292775	7.81202E-12	upregulated
LINC01127	1.617801509	1.09105E-14	upregulated
NPSR1	1.61787562	5.89666E-05	upregulated
**CXCL10**	1.619430812	5.20306E-12	upregulated
PSMD6.AS2	1.620722628	1.29206E-16	upregulated
KLRD1	1.621090277	7.51332E-15	upregulated
RPL9P32	1.624281692	1.94752E-12	upregulated
B3GNT4	1.625592764	2.70748E-23	upregulated
SPRR1A	1.625772239	4.56225E-05	upregulated
MUC2	1.627752568	4.21811E-05	upregulated

SNHG22	1.631769485	6.03829E-16	upregulated
CTSW	1.635761171	7.65952E-18	upregulated
SPTBN1.AS2	1.635890809	6.13499E-18	upregulated
CHMP3.AS1	1.636275637	1.18457E-12	upregulated
SERPINA1	1.637514151	2.17055E-10	upregulated
H2BC6	1.638939458	5.52109E-11	upregulated
GHRLOS	1.641449974	2.67319E-18	upregulated
TUBB8P1	1.643605471	3.41936E-17	upregulated
CRYZP1	1.646772889	2.82253E-13	upregulated
TH2LCRR	1.647389283	2.98367E-13	upregulated
MUC3A	1.64830198	8.64495E-21	upregulated
IL31RA	1.650133447	2.07303E-14	upregulated
MUC17	1.652618436	2.71848E-05	upregulated
LINC02334	1.65294353	2.64544E-11	upregulated
CAMTA2.AS1	1.653129119	7.53461E-21	upregulated
LYPD3	1.654780254	2.58365E-13	upregulated
LRRC8C.DT	1.657098766	1.86053E-17	upregulated
MATR3	1.658200192	6.54007E-13	upregulated
IGLV1.36	1.658449169	2.96779E-07	upregulated
RDH10.AS1	1.662525149	1.90191E-09	upregulated
PI3	1.66440865	5.59037E-08	upregulated
FASLG	1.665125673	1.11106E-22	upregulated
H2BC15	1.666576641	9.91936E-17	upregulated
TTC36.AS1	1.667185609	1.69999E-17	upregulated
FPR2	1.668970843	6.83015E-10	upregulated
HLA.U	1.669099717	1.93137E-07	upregulated
CYP1A1	1.670543191	4.8514E-06	upregulated
HOXC8	1.672394065	1.70891E-10	upregulated
PEG13	1.67305999	4.79155E-14	upregulated
SPNS2.AS1	1.673358327	3.78323E-17	upregulated
DNAAF3	1.676009047	8.14429E-21	upregulated
TRPM8	1.677613058	1.2115E-10	upregulated
KIAA1549L	1.677664344	3.17758E-10	upregulated
LINC02068	1.678444777	1.00818E-15	upregulated
MED28.DT	1.682004468	6.45461E-19	upregulated
HOXC9	1.682474366	1.53521E-11	upregulated
DRGX	1.684000014	1.38622E-07	upregulated
CCK	1.686316183	4.85152E-07	upregulated
ARX	1.688049814	1.64398E-08	upregulated
KRT7.AS	1.688485075	6.11624E-13	upregulated
SPDYE2	1.689416683	5.40051E-17	upregulated
RPH3AL.AS1	1.690960154	1.06597E-16	upregulated
PABPN1P1	1.695512389	9.89536E-18	upregulated
PKN2.AS1	1.696352793	1.17154E-14	upregulated
DOC2B	1.69672967	1.81144E-14	upregulated
TTC24	1.697122006	1.20787E-16	upregulated
RNF213.AS1	1.698773485	3.73733E-22	upregulated
PLEKHG4B	1.699322532	7.80814E-12	upregulated
SNORD3A	1.699415544	8.43515E-06	upregulated
GJB5	1.702322793	6.0068E-08	upregulated

CHRFAM7A	1.702855009	3.82325E-15	upregulated
LINC00589	1.703927012	1.62094E-19	upregulated
LINC00941	1.708123567	1.39667E-11	upregulated
CTSE	1.708693217	2.80996E-05	upregulated
KCNRG	1.709095874	3.03523E-19	upregulated
ZBED6	1.711669853	1.32528E-20	upregulated
INSYN2B	1.714238423	2.91562E-10	upregulated
PNPLA3	1.715744403	1.67057E-11	upregulated
MAPK12	1.716639297	1.9028E-23	upregulated
HLA.DOA	1.716727614	2.35299E-16	upregulated
CA8	1.717285888	3.27833E-09	upregulated
KRT17	1.721338291	2.14686E-08	upregulated
TICAM2.AS1	1.723915986	1.47079E-22	upregulated
AIM2	1.724401476	7.66975E-12	upregulated
ANKRD45	1.728411468	2.07698E-12	upregulated
HOMER2	1.730975926	6.21912E-13	upregulated
EMX1	1.731137279	4.34216E-09	upregulated
LINC02195	1.731835413	7.87186E-25	upregulated
CRYBA4	1.732183652	8.91969E-17	upregulated
TFAP2A.AS1	1.733516451	1.64792E-17	upregulated
CAPN14	1.735755892	6.95871E-14	upregulated
ERFE	1.737808163	3.65511E-16	upregulated
KCNC1	1.737809774	7.30134E-12	upregulated
RUNX1.AS1	1.737961281	2.25088E-07	upregulated
ANGPTL7	1.738710074	2.55367E-09	upregulated
GBP1P1	1.739007723	1.24793E-20	upregulated
KCNQ5	1.749646183	3.90769E-13	upregulated
H2BC8	1.749793547	3.32464E-12	upregulated
DPH1.AS1	1.750718159	9.95448E-25	upregulated
SLC6A14	1.751029524	9.35264E-10	upregulated
MT.ND6	1.752160529	4.46064E-16	upregulated
PHB1P19	1.752635281	3.64894E-16	upregulated
FAM169BP	1.755559236	2.14714E-12	upregulated
LINC00624	1.758597464	1.11403E-14	upregulated
LINC01303	1.76036906	3.88795E-15	upregulated
SLC16A7	1.763750639	3.28937E-14	upregulated
ZIC5	1.76448995	2.81276E-07	upregulated
DHDDS.AS1	1.767894983	1.66693E-17	upregulated
C8G	1.768087323	2.30266E-13	upregulated
CAMK2N2	1.769680188	4.20747E-19	upregulated
ZBTB47.AS1	1.770473549	1.56884E-10	upregulated
CD274	1.772058388	2.34679E-23	upregulated
FAM177B	1.773004483	1.48651E-10	upregulated
ARK2C	1.773079747	5.84013E-19	upregulated
GZMH	1.773152822	3.31347E-19	upregulated
EML5	1.77378839	3.07419E-21	upregulated
LINC01443	1.774777896	1.72341E-10	upregulated
TRNP1	1.775945763	2.01193E-13	upregulated
TNFSF9	1.776606972	1.69555E-14	upregulated
DIRAS3	1.777583629	3.06821E-16	upregulated

RN7SL809P	1.782648944	2.80076E-22	upregulated
TATDN1P1	1.782848353	1.24791E-08	upregulated
H3C14	1.786853251	4.77137E-14	upregulated
GALNT13	1.79060079	8.1274E-10	upregulated
CHST6	1.792169104	3.76835E-21	upregulated
GAPDHP61	1.79330469	9.36647E-16	upregulated
MYRF.AS1	1.794303217	1.01981E-15	upregulated
NPTN.IT1	1.796714467	2.24815E-18	upregulated
NADK2.AS1	1.797082924	4.45423E-15	upregulated
H2AC8	1.799329263	1.16853E-12	upregulated
AQP5	1.802623818	3.73577E-05	upregulated
FRMPD2	1.803899479	9.80415E-11	upregulated
LAG3	1.804494908	1.57432E-24	upregulated
FAM167A	1.806541306	4.70701E-21	upregulated
ODCP	1.809557942	8.13041E-19	upregulated
A2ML1	1.80974311	1.32646E-06	upregulated
KISS1R	1.811760509	8.96969E-15	upregulated
DNAH2	1.813625304	1.99317E-22	upregulated
PRSS41	1.813954302	1.5761E-10	upregulated
SPTB	1.814482468	2.67781E-30	upregulated
CALR4P	1.814504788	5.82289E-15	upregulated
RBM44	1.814587355	3.24044E-23	upregulated
ONECUT3	1.815125873	8.08995E-06	upregulated
VRTN	1.816077887	1.08132E-12	upregulated
SNPH	1.817035474	6.27759E-30	upregulated
CELF2.AS1	1.818010682	1.90719E-14	upregulated
KRR1P1	1.818800475	2.98371E-09	upregulated
ZNF578	1.822857849	1.35951E-16	upregulated
CXCL9	1.828148989	3.00373E-14	upregulated
RAB3B	1.828381262	1.59381E-14	upregulated
SAA2	1.828526973	1.92329E-11	upregulated
H3P16	1.830633105	2.21256E-47	upregulated
H1.2	1.833720245	8.46526E-21	upregulated
AGR2	1.838111557	1.89182E-25	upregulated
XKR9	1.840541497	4.72541E-14	upregulated
COL6A5	1.844020996	1.16202E-13	upregulated
ADGRG6	1.845529139	7.11467E-26	upregulated
PLAAT4	1.85381854	2.05168E-19	upregulated
INSM1	1.854649951	9.06591E-08	upregulated
PDE10A	1.860910264	2.48457E-22	upregulated
CLEC4D	1.86140488	1.66589E-14	upregulated
GZMA	1.86250657	3.58571E-25	upregulated
BEX1	1.866276638	2.07079E-13	upregulated
CLDN20	1.867912464	6.25738E-15	upregulated
FOXD1	1.869561292	3.54644E-11	upregulated
H4C8	1.8719619	1.28689E-15	upregulated
RAMP1	1.873200138	5.71557E-13	upregulated
PRDM8	1.87524139	2.20683E-21	upregulated
TTN	1.879488854	1.56467E-12	upregulated
MTCO1P53	1.88080821	9.02022E-08	upregulated

ZNF410	1.885770807	1.62941E-21	upregulated
JPX	1.886609139	6.62132E-14	upregulated
POGLUT2P1	1.886707041	1.76875E-15	upregulated
GFI1	1.886866918	3.07055E-24	upregulated
H3C8	1.88750159	4.3086E-12	upregulated
CFAP46	1.890448907	1.78664E-15	upregulated
IGFL1	1.893534461	1.44318E-08	upregulated
TGM1	1.896406916	1.71977E-27	upregulated
CCNYL6	1.898873895	1.91028E-13	upregulated
RAB27B	1.905543172	8.00416E-22	upregulated
DLEU7	1.907271321	2.60527E-10	upregulated
MTND4P12	1.907395594	4.44131E-08	upregulated
GPR82	1.908197784	3.68772E-15	upregulated
CD109	1.91584431	7.30859E-17	upregulated
LINC02826	1.915973542	4.45272E-11	upregulated
GJB6	1.916640424	1.81958E-13	upregulated
ASS1P11	1.917127565	5.95992E-13	upregulated
DEFA5	1.917427295	0.000300017	upregulated
HAR1B	1.918040428	1.30977E-18	upregulated
VSTM5	1.920076092	2.05624E-22	upregulated
BEGAIN	1.92201605	2.18736E-35	upregulated
DLG3.AS1	1.922533563	1.29153E-27	upregulated
MIR29B2CHG	1.923273075	9.89674E-15	upregulated
NEAT1	1.925347153	3.02486E-14	upregulated
FLG	1.928876447	3.77191E-14	upregulated
ERVW.1	1.932551412	4.62743E-09	upregulated
PA2G4P6	1.93420274	5.90034E-19	upregulated
CKLF	1.936719898	2.30735E-17	upregulated
S100A12	1.941120427	5.64365E-16	upregulated
SLC25A48	1.95234125	5.07546E-25	upregulated
SULT1C3	1.955914449	5.08735E-11	upregulated
PLCXD3	1.956132177	8.72414E-13	upregulated
MAP3K5.AS1	1.957301236	3.95569E-19	upregulated
TFAP2A	1.96072414	6.71215E-17	upregulated
LINC01522	1.967437787	2.48162E-18	upregulated
ADAMTSL4.AS	1.970272931	4.9739E-17	upregulated
LINC02489	1.973115568	9.15093E-22	upregulated
LINC02700	1.97408079	1.0665E-12	upregulated
HSD17B1	1.974657399	1.32658E-30	upregulated
ALOX12B	1.98270267	8.77816E-25	upregulated
ABCA12	1.982737432	3.87827E-10	upregulated
EML4.AS1	1.98584261	1.11673E-23	upregulated
COLGALT2	1.989013865	6.2544E-12	upregulated
PRSS21	1.990520654	7.10398E-07	upregulated
MT.RNR1	1.991273805	3.38102E-24	upregulated
RPL3P6	1.99285151	7.32715E-19	upregulated
RPS15AP30	1.997031159	2.1263E-15	upregulated
GBP4	2.000074702	3.8809E-27	upregulated
KLRC1	2.001140104	1.68853E-30	upregulated
USP3.AS1	2.001888596	6.98687E-16	upregulated

LINC02649	2.006417048	2.79784E-18	upregulated
H2BC26	2.006566117	1.20955E-24	upregulated
NXF3	2.007386561	9.39094E-09	upregulated
CCDC144BP	2.00799639	7.58546E-15	upregulated
NTSR1	2.008130331	9.06869E-10	upregulated
SPDYA	2.008331678	2.98768E-21	upregulated
NKX3.1	2.008347252	8.96744E-34	upregulated
KIZ.AS1	2.009342908	5.09411E-14	upregulated
SAA1	2.011553607	4.30062E-10	upregulated
H2AC11	2.012673413	3.36092E-17	upregulated
EPB41L1.AS1	2.015924757	1.3929E-13	upregulated
RERE.AS1	2.016016616	1.30728E-18	upregulated
INSYN2A	2.016726616	4.82059E-13	upregulated
CIITA	2.020066747	1.21166E-28	upregulated
HNRNPH1P1	2.020999194	1.09816E-21	upregulated
LRRTM1	2.028054899	2.76871E-09	upregulated
CCL26	2.033040136	8.09207E-21	upregulated
LURAP1L.AS1	2.041923409	5.46364E-14	upregulated
AQP3	2.044805748	3.01772E-16	upregulated
NRBF2P5	2.049334842	1.15024E-20	upregulated
IFNG	2.049644068	1.00016E-27	upregulated
IL36RN	2.05183861	5.39202E-16	upregulated
MTHFD1P1	2.060316667	9.73682E-17	upregulated
GPR18	2.068600883	7.55922E-15	upregulated
LINGO4	2.07081719	1.48369E-28	upregulated
RPL9P28	2.075058065	3.16196E-13	upregulated
FAM106A	2.075466753	1.42229E-17	upregulated
ELOVL3	2.077833926	1.31612E-37	upregulated
SYT12	2.078019091	4.01018E-25	upregulated
CP	2.080210745	3.88227E-13	upregulated
BBOX1	2.082640219	6.95195E-11	upregulated
RARRES1	2.08396751	1.20955E-24	upregulated
MTND4P35	2.091761764	6.63936E-18	upregulated
CA7	2.094907346	1.10217E-13	upregulated
SNORA80B	2.10013965	3.90816E-15	upregulated
HNRNPA3P9	2.101433398	9.24181E-26	upregulated
**HCAR2**	2.101992928	9.23113E-14	upregulated
BCL6.AS1	2.103345268	1.32528E-20	upregulated
RFX6	2.106205601	7.80858E-12	upregulated
DCDC2B	2.111712011	2.14688E-24	upregulated
SREBF2.AS1	2.116406115	5.50138E-26	upregulated
UNC5B.AS1	2.121915282	2.94153E-29	upregulated
ALOXE3	2.126717431	8.48917E-30	upregulated
FAT2	2.133878167	2.41112E-31	upregulated
CCDC141	2.134287243	6.31991E-19	upregulated
PKP4.AS1	2.135997553	4.16911E-24	upregulated
DIPK1C	2.136118852	9.07185E-21	upregulated
CHRNA7	2.13736753	1.86026E-18	upregulated
NUDT5P1	2.138680288	4.4203E-16	upregulated
CPS1	2.13868098	6.80236E-09	upregulated

NPM1P26	2.146360461	8.38428E-20	upregulated
**ALOX15B**	2.149003478	2.22625E-15	upregulated
GPR21	2.152657387	3.76835E-21	upregulated
FBXW10B	2.161515446	2.44135E-32	upregulated
SNORD89	2.166630884	4.99449E-22	upregulated
SCARNA6	2.169454274	1.94314E-08	upregulated
AGAP1.IT1	2.171178753	2.42034E-30	upregulated
S100A8	2.172133577	8.13041E-19	upregulated
REG1A	2.185838903	1.47639E-05	upregulated
MAL2.AS1	2.185921471	1.19481E-15	upregulated
PRNCR1	2.187008385	9.97238E-13	upregulated
NTRK3	2.192941163	1.72108E-18	upregulated
IL17REL	2.194663758	1.48814E-23	upregulated
IDO1	2.194832485	8.36435E-18	upregulated
SMTNL1	2.199510897	7.58819E-37	upregulated
GBP5	2.199934219	5.7353E-23	upregulated
XXYLT1.AS2	2.205359329	2.73941E-26	upregulated
MUC5B	2.2071878	1.27174E-11	upregulated
NCR1	2.213108914	1.69722E-38	upregulated
HOXC10	2.222576567	2.11415E-14	upregulated
DUSP4	2.225114193	2.02689E-23	upregulated
PRDX3P1	2.227495764	1.1721E-30	upregulated
H3C3	2.238297468	2.98371E-09	upregulated
HCAR3	2.246153819	9.6537E-17	upregulated
SLITRK5	2.246391306	6.81426E-17	upregulated
GABRP	2.246618038	8.15052E-12	upregulated
RPL29P14	2.24814428	6.78421E-29	upregulated
SNORA79B	2.249291225	8.69765E-10	upregulated
NEUROG3	2.251163297	2.07304E-16	upregulated
RNU6.1016P	2.25389224	2.57084E-17	upregulated
HOXC6	2.259786728	1.39743E-17	upregulated
PCDHGA8	2.277057089	1.15141E-17	upregulated
H2BC11	2.278463082	6.44176E-20	upregulated
LINC01257	2.27860519	1.04181E-28	upregulated
UBE2R2.AS1	2.288250119	9.43796E-26	upregulated
NME9	2.288963704	3.16539E-30	upregulated
RHOQ.AS1	2.290663967	6.28586E-17	upregulated
KCNQ1OT1	2.292159503	5.06596E-17	upregulated
BCL10.AS1	2.29216846	1.44606E-21	upregulated
WFDC21P	2.307424137	1.43791E-27	upregulated
TRPC6P2	2.315699264	2.34727E-20	upregulated
SERPINB2	2.317866364	7.75686E-14	upregulated
LINC01913	2.322091575	7.13702E-15	upregulated
ZBTB20	2.322742516	5.38591E-14	upregulated
ITGAD	2.329146994	7.49166E-38	upregulated
LINC00216	2.331678198	1.15024E-20	upregulated
NDUFV2	2.337987484	1.02284E-27	upregulated
ZNF683	2.347976759	2.72238E-29	upregulated
SLC34A2	2.351939981	1.41891E-14	upregulated
S100A7	2.354896757	3.72468E-11	upregulated

ARHGEF38.IT1	2.356973914	1.23163E-24	upregulated
ANKRD36C	2.358396436	7.52454E-27	upregulated
DHRS2	2.358905843	5.35127E-16	upregulated
HMSD	2.363828196	1.73848E-35	upregulated
ATP5PDP4	2.368803322	1.12685E-22	upregulated
SERPINB7	2.37059386	6.31593E-14	upregulated
ZNF460	2.372632302	4.59368E-23	upregulated
KIR2DL4	2.386186358	2.68361E-36	upregulated
LOXL2.AS1	2.389961369	1.43793E-25	upregulated
VNN2	2.399168755	5.03773E-29	upregulated
EDDM13	2.40012529	1.47858E-24	upregulated
GNLY	2.401336695	1.18031E-33	upregulated
RN7SKP80	2.438038975	7.33612E-30	upregulated
RNU2.6P	2.442118524	8.00416E-22	upregulated
PLA2G2F	2.446681914	1.0129E-15	upregulated
SNORA73B	2.450170057	1.04022E-07	upregulated
GBP6	2.455248241	2.97492E-40	upregulated
FYB2	2.461176868	7.11405E-26	upregulated
OMG	2.463124993	5.50718E-15	upregulated
IGFALS	2.466056486	4.13876E-14	upregulated
SUMO2P17	2.476501578	9.39775E-89	upregulated
KLK13	2.487865917	3.57381E-19	upregulated
MTND4P20	2.490395672	2.48773E-21	upregulated
REG4	2.490783772	1.6391E-09	upregulated
DIAPH2.AS1	2.496561456	1.84417E-18	upregulated
H2BC18	2.497102896	1.23749E-21	upregulated
MTND1P23	2.512822099	1.12494E-06	upregulated
STARD13.AS	2.514306244	1.89495E-13	upregulated
PM20D1	2.521018352	7.29728E-28	upregulated
TRIM72	2.539097051	4.13817E-13	upregulated
SNORD17	2.543108812	1.44537E-12	upregulated
ULBP2	2.5439835	1.64968E-42	upregulated
LY6D	2.559584149	1.66319E-15	upregulated
HRK	2.57072947	6.63374E-26	upregulated
CFLAR.AS1	2.5914343	5.66672E-21	upregulated
HOXC.AS2	2.593224052	3.54722E-24	upregulated
VNN1	2.599204271	1.09841E-21	upregulated
MIOX	2.608085073	4.77025E-33	upregulated
EEF1A1P50	2.613638715	1.62757E-30	upregulated
LINC02575	2.618917534	7.4884E-21	upregulated
LRRTM2	2.622611681	2.38511E-18	upregulated
HNF4A.AS1	2.62898927	3.1406E-15	upregulated
MTND5P11	2.639171161	5.08693E-21	upregulated
IL36G	2.654037488	4.91198E-33	upregulated
NXPH4	2.674793442	1.52576E-18	upregulated
CRADD.AS1	2.68506437	9.40215E-21	upregulated
SNORD15B	2.691400225	8.50428E-17	upregulated
PTMAP4	2.69427894	1.43805E-93	upregulated
NBPF7P	2.695691472	1.89182E-25	upregulated
PIWIL1	2.710332863	9.53968E-22	upregulated

M1AP	2.728263094	6.04658E-34	upregulated
MTND6P4	2.747981735	1.51972E-34	upregulated
H2BC4	2.758116131	5.93662E-26	upregulated
GSN.AS1	2.75968085	2.99454E-28	upregulated
SBSN	2.773745822	4.94905E-34	upregulated
HNRNPA1P14	2.779141738	8.96744E-34	upregulated
IL17C	2.786263071	3.32656E-32	upregulated
H2AC20	2.798581024	1.66081E-25	upregulated
LINC02446	2.802629725	1.02926E-45	upregulated
TP63	2.814087074	2.48951E-32	upregulated
MUC5AC	2.819082735	9.97596E-12	upregulated
PRSS2	2.857459942	1.22221E-08	upregulated
H2BC7	2.865990804	8.37464E-24	upregulated
SHROOM3.AS	2.905508493	1.22939E-37	upregulated
RNU4.2	2.950953297	2.21951E-09	upregulated
TNNT1	2.970909107	4.28695E-25	upregulated
GTF2I.AS1	2.997842867	3.29147E-23	upregulated
PCA3	3.011022714	1.00123E-20	upregulated
RN7SL1	3.01371304	6.17418E-16	upregulated
TRIM7	3.016722227	4.24216E-38	upregulated
TFF2	3.023030889	2.65009E-14	upregulated
SPRR3	3.04028335	2.80941E-14	upregulated
PSMD10P1	3.045631893	5.0392E-23	upregulated
H4C5	3.053032431	7.4884E-21	upregulated
PGC	3.078488625	3.62592E-24	upregulated
SLC7A11.AS1	3.092613454	6.27759E-30	upregulated
MALAT1	3.102937529	3.57059E-21	upregulated
SEMG1	3.11091408	1.935E-24	upregulated
RN7SL3	3.124897997	1.3834E-17	upregulated
KRT13	3.146196988	4.64076E-29	upregulated
MSH4	3.160473806	7.39899E-73	upregulated
H2AC13	3.168449272	2.90107E-25	upregulated
AFAP1.AS1	3.177843005	2.43912E-17	upregulated
SMIM38	3.185049688	1.23627E-45	upregulated
CLDN18	3.201436241	4.36679E-11	upregulated
SPIN2A	3.212437096	5.59665E-40	upregulated
UTS2B	3.256832908	2.08745E-54	upregulated
ITGA6.AS1	3.26417666	6.47045E-31	upregulated
LINC02350	3.291996555	7.87162E-32	upregulated
MTATP6P2	3.307163347	2.8939E-26	upregulated
HMX3	3.312592118	1.23562E-37	upregulated
LINC01630	3.329590872	3.6E-41	upregulated
RBP3	3.340011201	1.41093E-46	upregulated
RN7SL5P	3.355630349	1.09859E-44	upregulated
RNU4.1	3.379107777	1.4062E-14	upregulated
MUCL3	3.381037887	2.67505E-33	upregulated
XACT	3.42082331	1.59393E-17	upregulated
PLAC4	3.426008569	2.59612E-25	upregulated
LINC02912	3.435146393	1.72719E-27	upregulated
H2BC13	3.439321742	2.65839E-32	upregulated

SCARNA7	3.445638145	5.85849E-16	upregulated
PSAPL1	3.474192791	7.24361E-38	upregulated
H1.5	3.572339792	1.22514E-18	upregulated
H3C2	3.58430243	2.21785E-21	upregulated
H2BC17	3.625030806	4.39088E-29	upregulated
DANT2	3.63836324	4.73108E-52	upregulated
KRT16	3.656186173	1.34878E-29	upregulated
H1.4	3.666632935	7.57518E-23	upregulated
TRPV6	3.694106424	6.5445E-35	upregulated
SPRR2A	3.697505149	9.41895E-26	upregulated
SPRR2D	3.729351655	2.87951E-27	upregulated
ANXA10	3.743606248	4.13586E-29	upregulated
KRT6C	3.745105183	2.21708E-47	upregulated
H3C12	3.751772531	7.48039E-34	upregulated
SPRR1B	3.757355906	4.59968E-27	upregulated
HNRNPA3P11	3.790089694	1.02393E-60	upregulated
**MTND4P24**	3.801826352	1.43997E-30	upregulated
GP2	3.880865468	9.28478E-23	upregulated
MT.TM	3.911050904	1.89756E-30	upregulated
CLCA2	3.976137762	2.53348E-40	upregulated
LINC00632	4.056656029	1.39391E-37	upregulated
KRT6A	4.075859523	6.56478E-28	upregulated
H4C4	4.100912102	7.24514E-31	upregulated
H1.3	4.136631072	9.02234E-34	upregulated
KRT5	4.162933789	7.03814E-22	upregulated
RN7SL4P	4.239641797	1.40119E-43	upregulated
DLGAP1.AS5	5.416555128	4.0625E-99	upregulated
MUC6	5.819888532	1.85374E-45	upregulated
KRT14	6.960938919	1.38424E-79	upregulated
PAEP	7.849475775	1.02442E-95	upregulated

Supplementary Table S1C TCGA Wilcoxon MMR-d/MSI-H vs. MMR-p/MSI-L+MSS DEGs

TCGA DEG	Wilcoxon		MMR-d/MSI-H vs. MMR-p/MSI-L+MSS
Gene	logFoldChange	FDR	Direction
NOTUM	-3.331482934	3.37723E-11	downregulated
SLC26A3	-3.236571756	2.71704E-13	downregulated
LCN15	-3.231348164	0.000167991	downregulated
PRSS56	-3.024597221	6.95251E-06	downregulated
SLC14A1	-2.93270557	4.50041E-05	downregulated
GNG4	-2.843843046	7.4381E-17	downregulated
KRT23	-2.760234175	8.67244E-14	downregulated
UGT2A3	-2.753909378	2.31777E-11	downregulated
MS4A12	-2.738698661	1.67535E-05	downregulated
KRT40	-2.702366191	5.60819E-07	downregulated
CA4	-2.679835812	5.11109E-05	downregulated
COL2A1	-2.590480925	2.86409E-13	downregulated
TNNC2	-2.580436118	1.89296E-17	downregulated
ZG16	-2.543310098	0.001997287	downregulated
WIF1	-2.518645183	1.28093E-09	downregulated
SCNN1B	-2.458860307	5.24639E-12	downregulated
QPRT	-2.43470401	8.18555E-18	downregulated
CELP	-2.398804263	8.18555E-18	downregulated
AQP8	-2.383796329	2.9187E-08	downregulated
NPTX2	-2.382846008	0.018585963	downregulated
GUCA2A	-2.302011356	4.82078E-09	downregulated
IGF2BP1	-2.296979067	2.43875E-06	downregulated
SLC30A2	-2.258554762	7.89198E-17	downregulated
GRM8	-2.249374736	1.64792E-15	downregulated
CXCL14	-2.229901658	3.89403E-14	downregulated
NKD1	-2.229155102	2.50299E-14	downregulated
ENPP3	-2.213394647	1.12624E-12	downregulated
CYP2B6	-2.196359879	3.3856E-15	downregulated
MAP7D2	-2.161690042	1.17713E-09	downregulated
FABP1	-2.155880641	1.71354E-12	downregulated
SLC39A5	-2.155717197	8.18555E-18	downregulated
CHGB	-2.152721091	0.000194123	downregulated
IGHA2	-2.131745726	0.000227837	downregulated
CACNG4	-2.121512643	1.0183E-12	downregulated
APCDD1	-2.085694775	2.37897E-13	downregulated
PTPRO	-2.05046321	1.2712E-11	downregulated
DIO3OS	-2.043183353	2.53503E-15	downregulated
CAB39L	-1.999308616	2.26144E-17	downregulated
C6orf15	-1.993602321	4.80271E-14	downregulated
CRIPTO	-1.9835065	1.23586E-15	downregulated
HMGCS2	-1.971171778	1.12395E-08	downregulated
SLC26A2	-1.970149009	2.49177E-08	downregulated
ASCL2	-1.965194298	6.4437E-16	downregulated
POU5F1B	-1.960116838	3.13838E-16	downregulated
GPR15LG	-1.948207781	2.04453E-13	downregulated
HUNK	-1.943714673	2.53503E-15	downregulated
PCSK1N	-1.942976361	1.1784E-06	downregulated

COL9A3	-1.941313613	1.73842E-05	downregulated
MEP1A	-1.93767214	7.77754E-12	downregulated
CHP2	-1.908822009	2.11183E-11	downregulated
MUC12	-1.90869882	2.57259E-10	downregulated
NTS	-1.905044786	0.012100999	downregulated
PCDH19	-1.899873406	1.62932E-08	downregulated
AXIN2	-1.899743593	2.43255E-16	downregulated
RUBCNL	-1.871197602	2.24509E-15	downregulated
STRA6	-1.868526385	6.92643E-07	downregulated
SLC39A2	-1.858635657	7.07893E-08	downregulated
CTTNBP2	-1.855546291	1.44241E-16	downregulated
SLC13A2	-1.854195989	6.71015E-12	downregulated
SLC22A11	-1.844960169	2.28138E-17	downregulated
ACSL6	-1.842360376	2.27373E-11	downregulated
XPNPEP2	-1.841161971	2.02038E-10	downregulated
DRD2	-1.817669461	7.86088E-14	downregulated
LINC02940	-1.817614779	4.80851E-10	downregulated
SYN3	-1.788840597	9.20489E-14	downregulated
CEL	-1.765625251	6.15723E-15	downregulated
NKD2	-1.756886273	5.05193E-13	downregulated
DPEP1	-1.751774505	1.29669E-10	downregulated
GPR143	-1.750440294	4.19099E-14	downregulated
NXPE4	-1.724734605	1.68407E-05	downregulated
VENTX	-1.720639157	8.68163E-05	downregulated
FOLR1	-1.717100261	1.79583E-07	downregulated
ACE2	-1.715081008	1.08302E-11	downregulated
GDF10	-1.707850914	0.000729948	downregulated
TMEM132C	-1.705449326	1.8542E-05	downregulated
SLC15A1	-1.695221113	1.07533E-10	downregulated
QPCT	-1.690003027	2.08213E-12	downregulated
PIPOX	-1.688655915	1.06405E-06	downregulated
SLC6A4	-1.688019736	1.58496E-11	downregulated
F10	-1.687006763	3.71507E-11	downregulated
TRPM6	-1.68680181	5.7173E-12	downregulated
URAD	-1.672148885	7.74183E-06	downregulated
SLC1A7	-1.668977041	1.27222E-10	downregulated
NOX1	-1.66531503	7.1525E-13	downregulated
VSTM2L	-1.662234142	3.62025E-05	downregulated
RBP2	-1.661617277	7.52031E-10	downregulated
MOGAT3	-1.661316036	1.37389E-14	downregulated
SELENBP1	-1.638930125	3.00133E-11	downregulated
WNT11	-1.638296646	2.03777E-10	downregulated
TUSC8	-1.632915765	1.43403E-13	downregulated
SLC13A3	-1.632216772	1.04304E-08	downregulated
RBP1	-1.62691316	1.05202E-07	downregulated
ABCB1	-1.62179944	2.41887E-10	downregulated
LEFTY1	-1.618569322	3.36846E-07	downregulated
PCP4	-1.611700305	1.09823E-07	downregulated
VAV3	-1.608772867	1.69698E-13	downregulated
MTTP	-1.599540075	0.004601393	downregulated

CEACAM7	-1.595180903	0.002530797	downregulated
CKMT2	-1.594935283	3.35635E-07	downregulated
COL9A1	-1.584972444	2.14535E-13	downregulated
LINC01411	-1.577694689	2.94935E-06	downregulated
CDHR1	-1.569132869	6.06891E-12	downregulated
MTCO3P12	-1.563535163	0.000350107	downregulated
IHH	-1.558346464	4.22625E-14	downregulated
PHYHIPL	-1.552359394	8.06711E-09	downregulated
PRR15	-1.552348649	4.31499E-18	downregulated
TG	-1.549764215	4.77511E-10	downregulated
CPLX2	-1.547580057	5.55533E-09	downregulated
TMEM176A	-1.546973105	7.07256E-14	downregulated
ERP27	-1.54532493	3.62515E-08	downregulated
SATB2.AS1	-1.539388775	6.07919E-14	downregulated
RNF43	-1.535214372	9.04157E-17	downregulated
PTPRD	-1.535017653	3.28499E-11	downregulated
CES1	-1.532120536	8.8476E-06	downregulated
C3orf85	-1.522359578	2.84104E-13	downregulated
EYA1	-1.515125414	0.000428567	downregulated
PKLR	-1.510973075	6.35749E-12	downregulated
UCA1	-1.509671852	8.56566E-05	downregulated
L1CAM	-1.50694644	0.002127868	downregulated
REEP1	-1.505707999	1.21663E-10	downregulated
SERPINA6	-1.499341785	3.77884E-08	downregulated
F7	-1.496624551	3.89403E-14	downregulated
ISM2	-1.488058674	1.93196E-11	downregulated
TINAG	-1.481665094	2.60647E-14	downregulated
TAC1	-1.474122612	7.11131E-09	downregulated
SESN1	-1.468047584	2.37897E-13	downregulated
AIFM3	-1.461269718	1.72657E-12	downregulated
TMIGD1	-1.460095778	0.000157873	downregulated
ABAT	-1.458902167	2.52078E-12	downregulated
PLA2G12B	-1.457439707	8.38338E-12	downregulated
LHFPL7	-1.457377468	3.12879E-07	downregulated
CLDN8	-1.457069822	0.000233807	downregulated
AREG	-1.449428569	2.94122E-09	downregulated
CYP4F2	-1.446632882	1.31405E-11	downregulated
CKB	-1.441994172	8.98542E-07	downregulated
MYOM3	-1.440920924	8.83132E-12	downregulated
ISX	-1.436002068	5.03064E-10	downregulated
FGGY	-1.434737233	5.05421E-06	downregulated
SLC2A12	-1.434496873	1.24004E-10	downregulated
SLC35D3	-1.434117451	1.75482E-11	downregulated
CFTR	-1.431093765	6.75096E-14	downregulated
VANGL2	-1.427802512	1.49709E-09	downregulated
R3HDML.AS1	-1.425644356	3.97912E-16	downregulated
TMEM252	-1.422698681	4.93298E-11	downregulated
POU6F2	-1.420603486	0.000127407	downregulated
SLC19A3	-1.417343862	1.41406E-10	downregulated
ARID3A	-1.414219919	7.10845E-14	downregulated

ID1	-1.412599314	2.92324E-09	downregulated
SLC51B	-1.412260212	1.19366E-10	downregulated
EPDR1	-1.409296954	1.52673E-14	downregulated
CLSTN2	-1.408040377	0.000148225	downregulated
ELF5	-1.407277802	1.03992E-06	downregulated
TMEM150C	-1.403905039	9.03747E-11	downregulated
CAPS	-1.403888467	5.17025E-10	downregulated
RNF182	-1.396609538	7.57497E-06	downregulated
PRAC1	-1.394325035	0.001443166	downregulated
PPP1R14D	-1.391947084	2.09823E-12	downregulated
SHISA9	-1.381915082	3.23782E-08	downregulated
PMEPA1	-1.379789959	8.19127E-14	downregulated
PHACTR3	-1.377453723	2.72683E-09	downregulated
FREM1	-1.367076181	1.5316E-06	downregulated
REN	-1.362781879	2.05722E-11	downregulated
TNFRSF19	-1.352942391	7.17418E-05	downregulated
TLE2	-1.352494652	1.05354E-11	downregulated
PGAP3	-1.347542988	5.4609E-08	downregulated
HCAR1	-1.339399533	2.1304E-07	downregulated
NR1I2	-1.335740964	2.66761E-13	downregulated
PLAGL2	-1.333468283	1.44241E-16	downregulated
MAGEB17	-1.332411164	1.86914E-11	downregulated
CTSV	-1.320221833	3.9702E-12	downregulated
NPFFR1	-1.31814462	1.10972E-12	downregulated
PLCB4	-1.316970452	4.66246E-12	downregulated
ANO9	-1.316813426	1.28177E-11	downregulated
MMP11	-1.316249281	3.07406E-05	downregulated
SYT7	-1.316077744	8.97743E-12	downregulated
LRRC36	-1.314861056	1.98207E-14	downregulated
ADH1C	-1.314366801	0.001373231	downregulated
B3GAT1.DT	-1.309537041	5.126E-10	downregulated
ENGASE	-1.307831007	6.35676E-15	downregulated
TMEM63C	-1.306921295	3.14915E-09	downregulated
CST1	-1.306836685	0.000463229	downregulated
MEX3A	-1.30603087	2.56024E-12	downregulated
MAGEB17.AS1	-1.299037386	2.23797E-10	downregulated
SMIM2.AS1	-1.295335342	4.90047E-12	downregulated
PALM3	-1.294253901	1.45743E-08	downregulated
NRXN2	-1.287570168	4.96558E-07	downregulated
DLX3	-1.281972153	2.5163E-11	downregulated
LGR6	-1.278140081	7.73499E-06	downregulated
GGT7	-1.273324654	1.49903E-12	downregulated
OXGR1	-1.267363172	2.17657E-08	downregulated
LRRC2	-1.266976597	7.34159E-13	downregulated
CPA6	-1.258582416	7.36509E-07	downregulated
TMEM176B	-1.257691746	1.52787E-12	downregulated
PTPRD.AS1	-1.257226565	4.22625E-14	downregulated
ABCC2	-1.25683271	0.000363606	downregulated
SOSTDC1	-1.254903176	2.18183E-07	downregulated
SGK2	-1.252381662	1.31405E-11	downregulated

POFUT1	-1.24979814	8.18555E-18	downregulated
MME	-1.24365381	0.02541379	downregulated
GGH	-1.24251738	3.02182E-12	downregulated
SULT1C4	-1.241007335	0.042283893	downregulated
SALL4	-1.238052697	4.09195E-10	downregulated
PRSS23	-1.238046447	4.66246E-12	downregulated
TBX10	-1.237861298	1.93389E-09	downregulated
FER1L4	-1.232269294	6.58065E-08	downregulated
FREM2	-1.232053427	1.53177E-12	downregulated
KRT20	-1.231551356	7.53729E-08	downregulated
RASL10B	-1.225089074	6.65017E-06	downregulated
PAH	-1.222797204	1.72675E-11	downregulated
SHISA6	-1.219920148	0.000344682	downregulated
CERNA2	-1.218941944	9.29581E-10	downregulated
PPP1R14C	-1.211610274	2.38704E-09	downregulated
FGF20	-1.209869588	2.85286E-07	downregulated
FZD10.AS1	-1.204441247	0.00895783	downregulated
FABP6	-1.193579458	2.15243E-08	downregulated
CTNNA2	-1.190935999	1.60395E-07	downregulated
GSPT2	-1.187115755	1.07585E-10	downregulated
SEMA5A	-1.185274713	1.08865E-13	downregulated
DIO3	-1.182069047	1.30909E-09	downregulated
SHROOM2	-1.180710979	3.77547E-10	downregulated
DDC	-1.180358295	3.49525E-10	downregulated
SLC5A6	-1.173420126	1.64792E-15	downregulated
ELAVL2	-1.171956678	6.56669E-12	downregulated
ATP9A	-1.171298247	2.98413E-15	downregulated
MYEF2	-1.169343058	1.62332E-08	downregulated
LINC00654	-1.165537151	3.84039E-09	downregulated
TMPRSS13	-1.161888121	2.70609E-06	downregulated
LINC02418	-1.16179353	4.03678E-08	downregulated
AGT	-1.160331128	1.63337E-09	downregulated
KHDRBS3	-1.158684371	9.05147E-12	downregulated
HSD11B2	-1.157965379	2.64892E-10	downregulated
CARD11	-1.155286537	0.025147958	downregulated
LCN12	-1.1517358	1.37561E-10	downregulated
TCF7	-1.151533024	3.3856E-15	downregulated
TRIM54	-1.15108634	3.72524E-05	downregulated
VWA5B1	-1.14751499	0.000463663	downregulated
TSPAN6	-1.147151767	2.79354E-14	downregulated
LRRC19	-1.147107939	2.34949E-09	downregulated
RASSF10	-1.144048682	1.30881E-07	downregulated
CLDN15	-1.139872097	3.14915E-09	downregulated
LINC02441	-1.139546509	3.3856E-15	downregulated
SLC38A4	-1.138176764	3.62515E-08	downregulated
HSPH1	-1.136441523	4.84622E-14	downregulated
AOAH	-1.135707713	6.16887E-10	downregulated
MAPRE3	-1.131025727	7.01752E-15	downregulated
RNLS	-1.126043468	2.08213E-12	downregulated
UPK3A	-1.125706609	1.91121E-05	downregulated

NTRK2	-1.122240518	0.015795246	downregulated
SPMIP5	-1.114215599	3.97575E-15	downregulated
ZNF606	-1.113967112	2.9655E-10	downregulated
MPP1	-1.111188675	1.0974E-09	downregulated
FCGRT	-1.108790636	1.44883E-12	downregulated
SERPINE2	-1.107957937	1.18239E-09	downregulated
RPS6KA6	-1.107843395	3.02343E-13	downregulated
DDAH2	-1.107274271	5.28932E-16	downregulated
NEUROD1	-1.106222261	0.004622095	downregulated
PTP4A3	-1.10250329	3.74324E-10	downregulated
OSER1.DT	-1.094907304	1.9534E-14	downregulated
PABPC1L	-1.090419011	6.16981E-07	downregulated
MUC20P1	-1.090004315	4.73431E-10	downregulated
MLH1	-1.087975586	7.34159E-13	downregulated
TGFBI	-1.08608156	1.70787E-07	downregulated
CPE	-1.085855407	1.53898E-07	downregulated
CAPN6	-1.085732439	0.000514045	downregulated
ZNF853	-1.084523859	1.96733E-09	downregulated
LAPTM4B	-1.08400566	1.05079E-10	downregulated
CEACAM6	-1.081598943	4.463E-07	downregulated
HOXD10	-1.078528379	3.33685E-05	downregulated
MUC20	-1.0782955	2.76194E-11	downregulated
BRSK2	-1.077879073	7.73499E-06	downregulated
WNT5B	-1.0760098	8.25296E-10	downregulated
UMODL1	-1.074139822	1.45951E-12	downregulated
ZSWIM3	-1.0739522	1.26843E-16	downregulated
SELENOP	-1.07366426	1.8542E-05	downregulated
APOLD1	-1.071246969	6.51543E-07	downregulated
PIGZ	-1.071159154	4.28475E-09	downregulated
CEBPA	-1.068816624	4.40069E-12	downregulated
HOXA3	-1.06739625	1.30816E-08	downregulated
SCARA5	-1.066880823	0.000121987	downregulated
IGFL4	-1.064764664	9.27382E-10	downregulated
SLC38A3	-1.064384924	2.79976E-09	downregulated
FADS6	-1.06297459	4.36654E-10	downregulated
NEK3	-1.062623953	2.39426E-13	downregulated
RTL8A	-1.061798497	1.58306E-08	downregulated
ALKAL1	-1.057627569	3.16435E-10	downregulated
TMEM236	-1.057412461	8.41819E-07	downregulated
GRB7	-1.057179919	0.001447457	downregulated
EREG	-1.056439963	2.4521E-09	downregulated
LINC02967	-1.055476554	5.03601E-12	downregulated
MLXIPL	-1.054613588	5.55602E-06	downregulated
CLDN3	-1.0537268	8.30666E-09	downregulated
BEX2	-1.052304261	7.12851E-08	downregulated
SUSD3	-1.051590535	3.40153E-05	downregulated
OGDHL	-1.051056605	8.69841E-11	downregulated
ST6GAL2	-1.050140327	0.000987956	downregulated
COL17A1	-1.048170104	5.41538E-05	downregulated
EGF	-1.047344563	0.006189384	downregulated

GUCA2B	-1.046934202	0.013739361	downregulated
VIL1	-1.041416679	1.85722E-13	downregulated
CAMKV	-1.040997192	5.60447E-07	downregulated
CCDC170	-1.038873316	9.44542E-08	downregulated
DDX27	-1.038623124	4.56782E-18	downregulated
R3HDML	-1.038530901	6.35676E-15	downregulated
EPHA7	-1.037936428	8.06144E-05	downregulated
CDHR5	-1.036320707	1.56431E-06	downregulated
EVX1	-1.03605324	2.96018E-08	downregulated
CLCN2	-1.033820075	5.42008E-14	downregulated
LINC02747	-1.033158238	5.65205E-10	downregulated
GLRA2	-1.032265695	1.5648E-05	downregulated
PRDX5	-1.032026008	6.21257E-10	downregulated
LPL	-1.031283311	7.21269E-05	downregulated
SLC38A11	-1.030583141	0.000124092	downregulated
SNCAIP	-1.030560456	9.46152E-06	downregulated
CCDC88B	-1.030528792	0.000320734	downregulated
CADPS	-1.030278144	3.04488E-07	downregulated
ARSL	-1.024661892	2.20981E-06	downregulated
TFCP2L1	-1.022032765	3.43E-10	downregulated
FITM2	-1.020235271	6.65309E-14	downregulated
PLA2G4F	-1.019163948	4.9874E-09	downregulated
MAGEA4	-1.015916862	0.000874411	downregulated
APOD	-1.014419787	0.000476371	downregulated
AMACR	-1.013833683	2.52078E-12	downregulated
SPIRE2	-1.013657748	2.15921E-13	downregulated
CDX2	-1.010956916	4.06252E-11	downregulated
ZNF503	-1.010324111	3.24696E-10	downregulated
ZNRF3	-1.010135613	7.01883E-13	downregulated
SERPINA10	-1.008854021	3.98505E-09	downregulated
ADGRF4	-1.008635375	1.39952E-06	downregulated
LIN7A	-1.007267669	0.04009343	downregulated
RAB32	-1.007074707	2.80043E-12	downregulated
SMOC2	-1.003710168	1.87537E-05	downregulated
PID1	-1.000203864	2.36505E-06	downregulated
SOWAHA	-1.000072386	6.875E-08	downregulated
CYBB	1.000030955	3.26185E-05	upregulated
PTMAP5	1.00088346	3.79386E-07	upregulated
PLBD2	1.00123327	0.005010496	upregulated
LMO4	1.001941796	3.95784E-14	upregulated
AGAP2	1.002134005	6.679E-08	upregulated
NME9	1.002882651	0.003179362	upregulated
LINC01443	1.002982461	0.02317212	upregulated
SULT1C2	1.003288966	0.00413958	upregulated
EPB41L1.AS1	1.003595958	0.001808041	upregulated
MCUB	1.004124685	1.06407E-14	upregulated
F3	1.006287174	1.21079E-07	upregulated
TNFAIP2	1.006342339	5.26767E-06	upregulated
H2BC26	1.006396146	0.048668636	upregulated
IRF1	1.007198383	2.17657E-08	upregulated

POLR2A	1.007611284	2.23163E-08	upregulated
PTAFR	1.00828272	6.19652E-09	upregulated
AQP9	1.008286057	8.95555E-05	upregulated
PIAS2	1.008922581	7.4381E-17	upregulated
ASRGL1	1.0094503	0.00018003	upregulated
C1QA	1.009537244	0.00043797	upregulated
CD84	1.011318367	0.000101271	upregulated
SLFN5	1.012456557	2.57598E-08	upregulated
SLC28A3	1.017615563	0.000471772	upregulated
MMP25.AS1	1.018143339	0.030176714	upregulated
RAC3	1.019580031	1.27562E-08	upregulated
KLK10	1.02036677	0.003661544	upregulated
GBP2	1.022050444	4.33794E-10	upregulated
PIK3AP1	1.022158004	4.88396E-10	upregulated
MSR1	1.022758143	0.0002144	upregulated
ARHGEF4	1.022949535	0.005342681	upregulated
FAM83A	1.023820618	0.013167521	upregulated
TOX	1.024550992	6.21063E-05	upregulated
FGFR2	1.024932903	1.98556E-05	upregulated
CEP85L	1.025099925	2.63022E-12	upregulated
AFAP1L2	1.025300714	1.21494E-09	upregulated
TNFRSF11A	1.026203707	5.59054E-09	upregulated
KDELR3	1.028216021	2.56956E-11	upregulated
HLA.DRB6	1.029474958	0.003881871	upregulated
MAP3K6	1.029979031	3.47685E-11	upregulated
SULT1C3	1.030055607	0.000615623	upregulated
CAMK2N2	1.030342716	1.09704E-08	upregulated
PLA2G3	1.030453111	4.70266E-06	upregulated
MICB	1.033830424	5.15002E-11	upregulated
DIAPH2.AS1	1.034854554	0.000395153	upregulated
IL17C	1.037472117	0.000353954	upregulated
FYB1	1.038968003	0.000188687	upregulated
NEUROG3	1.039399631	0.017446004	upregulated
SLC2A5	1.039685611	1.0348E-06	upregulated
FAM13A.AS1	1.041255273	0.012264792	upregulated
ADAM28	1.042396051	7.78697E-08	upregulated
PCDHB13	1.043735339	0.009439083	upregulated
APOL6	1.043853506	4.70346E-13	upregulated
USP18	1.044368271	7.24269E-12	upregulated
TIAM1	1.044800411	1.34371E-05	upregulated
WNT7B	1.045054291	4.6883E-05	upregulated
SLC1A1	1.047929724	1.30909E-09	upregulated
BMAL2	1.050820812	3.68323E-14	upregulated
BCAT1	1.051562407	8.13285E-05	upregulated
DLEU7	1.053021453	0.00043797	upregulated
APOL4	1.055012604	1.04851E-06	upregulated
SYT13	1.055349284	0.00061789	upregulated
LINC02350	1.056409743	5.29934E-05	upregulated
GALNT5	1.056553301	3.45089E-09	upregulated
DPYSL2	1.057998255	4.84702E-08	upregulated

PTGER2	1.058229122	9.02077E-06	upregulated
ANXA1	1.058922142	0.003154294	upregulated
MDM2	1.060476417	7.10315E-15	upregulated
CA9	1.066005598	0.000560262	upregulated
TCP11L2	1.071181505	9.7017E-10	upregulated
KCNK1	1.071931892	3.71632E-13	upregulated
GCNT3	1.072929114	9.84353E-05	upregulated
CXCL11	1.073648786	0.000148225	upregulated
HOXC11	1.07427248	0.005106464	upregulated
PARP14	1.076203606	1.18826E-10	upregulated
RAB26	1.076476071	2.06562E-07	upregulated
RNF125	1.077015809	1.50551E-14	upregulated
MSH4	1.077386707	4.94897E-15	upregulated
LINC02649	1.08109894	1.71757E-05	upregulated
MUC1	1.083114678	2.62288E-05	upregulated
LINC02446	1.083735217	4.84622E-14	upregulated
LINC00941	1.083886891	4.90255E-10	upregulated
SPHK1	1.085654531	5.02445E-05	upregulated
TNRC6B	1.085663032	4.33106E-11	upregulated
CCNP	1.087276813	0.000103552	upregulated
APOL2	1.08964436	3.78989E-12	upregulated
IRX2	1.092248011	0.000184829	upregulated
SHF	1.0936758	8.03758E-05	upregulated
UST	1.093762961	0.008807453	upregulated
HLA.DQB1	1.095072878	7.04583E-05	upregulated
IFI6	1.097470606	0.001874512	upregulated
FRAS1	1.097486639	0.000414371	upregulated
ATP5PDP4	1.099642708	0.015921791	upregulated
PRF1	1.100501499	1.49418E-10	upregulated
LINC02688	1.101867867	5.06716E-09	upregulated
IL2RB	1.102419887	2.40087E-08	upregulated
C1QB	1.102567684	0.000116273	upregulated
EDDM13	1.105549303	0.012891978	upregulated
FCGR3B	1.108545135	5.99077E-05	upregulated
DPYD	1.108641229	5.22191E-07	upregulated
UBE2L6	1.109216975	5.47973E-09	upregulated
KLK11	1.110150529	0.000836255	upregulated
MUC16	1.112262252	0.032182693	upregulated
TGM1	1.11231847	0.030707214	upregulated
HCAR3	1.114304792	0.001878058	upregulated
TANC2	1.115015565	9.6849E-08	upregulated
S100A9	1.120570099	0.037305979	upregulated
APOBEC3G	1.123827892	2.50937E-07	upregulated
SP140	1.129628232	7.07684E-08	upregulated
TNC	1.130519748	0.024417447	upregulated
PLCL2	1.133920756	1.07411E-11	upregulated
GPR82	1.136911462	0.012066729	upregulated
RASGRP1	1.138737038	6.35749E-12	upregulated
TLR8	1.142277999	5.30586E-06	upregulated
SOX8	1.142349084	0.000733668	upregulated

PLEK	1.144966833	1.88143E-06	upregulated
IFIT2	1.145908586	2.97742E-07	upregulated
RNF152	1.147163741	2.221E-10	upregulated
GBP1P1	1.148896746	1.03638E-08	upregulated
ZNF618	1.150047748	1.58726E-12	upregulated
TP73	1.150258784	1.54813E-08	upregulated
LINC02575	1.152928373	0.01588044	upregulated
XAF1	1.153169431	1.5216E-06	upregulated
KYNU	1.153361186	3.39326E-08	upregulated
JAK2	1.154586037	2.53503E-15	upregulated
HAPLN3	1.158443985	3.12611E-09	upregulated
HLA.DQA1	1.158944249	9.84353E-05	upregulated
MMP1	1.160667583	0.000816055	upregulated
M1AP	1.161795331	3.84422E-06	upregulated
CSF3R	1.162458586	0.000103552	upregulated
GATA4	1.165337445	0.002726834	upregulated
PNPLA3	1.170076107	4.90255E-10	upregulated
HLA.DMB	1.171791738	1.72785E-06	upregulated
BEGAIN	1.176016902	2.23588E-10	upregulated
ANKRD36	1.176506125	0.009985434	upregulated
FPR2	1.176792543	1.43906E-05	upregulated
SMIM33	1.178026857	5.23549E-06	upregulated
CXCR4	1.178128242	2.01856E-09	upregulated
NPTN.IT1	1.17832035	0.04009343	upregulated
SLC25A48	1.17943706	2.40328E-06	upregulated
HLA.DPB1	1.181093703	1.27863E-05	upregulated
ANKRD36B	1.183562819	0.001072047	upregulated
LOXL1.AS1	1.184399971	3.75992E-12	upregulated
FCGR2A	1.187853638	3.95488E-07	upregulated
SERPINB2	1.187932301	3.3401E-06	upregulated
STS	1.189872326	2.99725E-07	upregulated
MIR924HG	1.190498226	7.83384E-09	upregulated
TENT5A	1.19105905	9.25659E-14	upregulated
CARINH	1.191469932	1.42406E-05	upregulated
LINGO1	1.193388554	0.027485385	upregulated
MRC1	1.193558126	0.000383172	upregulated
TYMP	1.197275536	5.24219E-09	upregulated
RNF144B	1.198272561	2.38477E-11	upregulated
REG1B	1.201485602	0.004999744	upregulated
MAMLD1	1.203360418	5.59254E-08	upregulated
CCL4	1.20551672	1.58932E-10	upregulated
ONECUT2	1.210576319	3.87937E-07	upregulated
AIF1L	1.2110107	7.29542E-08	upregulated
IFIT3	1.213710406	1.47109E-06	upregulated
MT2A	1.213834188	3.97671E-08	upregulated
FOXD1	1.216428673	9.23931E-08	upregulated
BARX2	1.216705909	1.02906E-05	upregulated
TCN1	1.224340756	2.26807E-05	upregulated
MIRLET7BHG	1.225358291	1.99306E-08	upregulated
SLC4A4	1.226036137	6.42635E-07	upregulated

SPTB	1.229977908	1.9657E-10	upregulated
DOCK5	1.233542599	1.57236E-16	upregulated
MLPH	1.234868663	3.0347E-08	upregulated
FSCN1	1.237343344	3.5321E-06	upregulated
SLC7A11	1.239398129	1.1192E-07	upregulated
OXCT1	1.239800147	4.45807E-14	upregulated
CD8A	1.241406376	4.65544E-08	upregulated
RAP1GAP	1.24204037	5.08509E-05	upregulated
PRKCG	1.244748542	0.002554667	upregulated
USP3.AS1	1.247014226	0.007404078	upregulated
GRAMD1B	1.247055978	4.40175E-08	upregulated
CD74	1.249934713	1.40943E-06	upregulated
ADGRF1	1.251673786	0.000594926	upregulated
CCL26	1.255979204	0.003081708	upregulated
MYRF	1.256506188	5.00915E-11	upregulated
CHRNA7	1.260321992	2.40142E-11	upregulated
PSAPL1	1.260520291	3.50309E-05	upregulated
C8G	1.261184926	0.016426645	upregulated
SLC34A2	1.262544549	0.000476019	upregulated
TFF1	1.264453892	0.017054663	upregulated
APOL1	1.264548026	1.14559E-09	upregulated
SLC43A3	1.265267417	7.50534E-07	upregulated
GZMH	1.268004692	3.00966E-08	upregulated
PRDM16	1.270929171	3.5321E-06	upregulated
HOMER2	1.273150165	0.000208582	upregulated
CTSW	1.276574927	3.68374E-11	upregulated
TMPRSS3	1.276819008	4.5858E-05	upregulated
GAD1	1.277079755	7.7717E-09	upregulated
ANO1	1.277983945	8.49126E-08	upregulated
MMP12	1.280004973	4.88647E-06	upregulated
SDR16C5	1.28172265	9.77141E-10	upregulated
SLC1A3	1.281872758	5.66426E-07	upregulated
CXCL8	1.282412226	8.8476E-06	upregulated
FAM177B	1.285356912	6.25725E-08	upregulated
FER1L6	1.286304614	5.1552E-05	upregulated
IL1RN	1.287505306	2.87835E-08	upregulated
ERFE	1.288284861	2.77721E-09	upregulated
NFIA	1.288584622	0.045198783	upregulated
SYT12	1.289252223	1.1219E-05	upregulated
ZIC2	1.289554102	2.5612E-06	upregulated
IFI44L	1.290534889	0.001152008	upregulated
CCDC68	1.291170331	3.06446E-13	upregulated
GPR18	1.293592623	0.006691859	upregulated
OAS2	1.293674428	3.77884E-08	upregulated
CBFA2T3	1.294275985	0.015066166	upregulated
PLA2G4A	1.295102498	1.15547E-09	upregulated
SAA2	1.296453345	0.001733541	upregulated
PNMA2	1.297621986	0.000264075	upregulated
HLA.DRB1	1.300807852	3.7825E-06	upregulated
ENO2	1.303026811	5.83906E-12	upregulated

FFAR4	1.307831395	2.02847E-06	upregulated
KLRD1	1.308482638	8.90405E-12	upregulated
PTGS2	1.314512364	9.77268E-06	upregulated
MIAT	1.318701664	2.35657E-05	upregulated
NKX3.1	1.319301711	6.86778E-14	upregulated
OMG	1.326738747	0.001701022	upregulated
CHST4	1.327644677	0.008874656	upregulated
MATR3	1.327827747	0.010950793	upregulated
HPSE	1.329380398	2.49069E-15	upregulated
HLA.DRA	1.330325511	6.16981E-07	upregulated
IL1R2	1.335527378	5.29284E-08	upregulated
CXCL13	1.336161712	4.80734E-08	upregulated
LRRTM1	1.340982795	4.91856E-07	upregulated
SERPINB5	1.352069904	9.97085E-06	upregulated
XKR9	1.352856448	1.03301E-10	upregulated
WARS1	1.35489906	8.44355E-10	upregulated
MTCO2P12	1.355311943	0.00722161	upregulated
UBASH3B	1.358451632	2.58484E-11	upregulated
SERPINB7	1.358942038	4.734E-05	upregulated
ETV5	1.359723077	1.7864E-14	upregulated
LYPD5	1.362401645	8.39363E-13	upregulated
GBP1	1.362969051	1.92725E-09	upregulated
AIM2	1.363519743	0.000252577	upregulated
BST2	1.371680588	4.11019E-07	upregulated
NKG7	1.372550034	2.82631E-10	upregulated
DANT2	1.373439057	4.08922E-06	upregulated
SAMD9L	1.374434526	1.60699E-11	upregulated
HLA.DPA1	1.374604281	6.63242E-05	upregulated
ASPHD2	1.376287677	2.53503E-15	upregulated
CALB1	1.379884059	0.000256976	upregulated
NDUFA4L2	1.381942676	0.002461687	upregulated
RN7SL5P	1.382071901	0.010008905	upregulated
FCGR3A	1.383436691	5.09993E-08	upregulated
APOL3	1.388652476	2.38477E-11	upregulated
NDUFV2	1.392490385	9.82768E-10	upregulated
RPL22L1	1.396161144	8.37244E-14	upregulated
LY6D	1.398800901	0.02433186	upregulated
SNORA79B	1.400545871	0.023297422	upregulated
CHST6	1.403995861	6.28232E-09	upregulated
ABCA3	1.404400846	5.05421E-06	upregulated
ZIC5	1.409547243	7.74513E-07	upregulated
ALDH1L1	1.413527723	4.86492E-05	upregulated
INSM1	1.417634949	0.000449511	upregulated
LINC00261	1.420806712	0.009947822	upregulated
PLLP	1.42165604	7.35908E-10	upregulated
ZNF683	1.427147804	2.68628E-11	upregulated
SHROOM3.AS	1.428251241	0.008881107	upregulated
DMBT1	1.429788666	0.011995788	upregulated
H3C3	1.431353504	0.024200583	upregulated
GJB5	1.431515341	5.89113E-10	upregulated

FBXL16	1.432368936	8.44355E-10	upregulated
TP63	1.439824943	0.001246178	upregulated
ATOH1	1.44476928	0.012968359	upregulated
NPSR1	1.446409056	4.86599E-05	upregulated
**OR2I1P**	1.447487078	1.16719E-05	upregulated
SPDEF	1.459406713	3.14991E-05	upregulated
HSPA4L	1.462461349	1.50492E-13	upregulated
CD163	1.464943972	3.46301E-05	upregulated
HLA.DRB5	1.468467607	1.73842E-05	upregulated
WFDC21P	1.471526301	1.87651E-10	upregulated
**HCAR2**	1.475573528	0.00397167	upregulated
LYZ	1.475673596	5.69316E-06	upregulated
ITGA6.AS1	1.480720317	0.005005893	upregulated
DEFA6	1.480912415	8.73627E-06	upregulated
PLK2	1.481263938	8.30666E-09	upregulated
VSTM5	1.484246017	4.08785E-11	upregulated
KIAA1549L	1.484580213	1.62034E-06	upregulated
CCL5	1.486775834	2.2426E-10	upregulated
MT1E	1.491742836	1.67594E-09	upregulated
MAPK12	1.498038393	1.325E-11	upregulated
MUC4	1.505152057	3.58272E-06	upregulated
PTPRU	1.505193059	1.69426E-07	upregulated
TRIB2	1.507271108	7.01752E-15	upregulated
SLC16A7	1.508863199	6.94807E-06	upregulated
HLA.DMA	1.50959412	1.13085E-06	upregulated
NXF3	1.511671027	5.90939E-11	upregulated
LAG3	1.512339647	3.54447E-09	upregulated
ABCA12	1.518392555	2.01807E-05	upregulated
DAPK1	1.524518109	3.31281E-11	upregulated
PRDM8	1.527826171	2.22534E-13	upregulated
CCL18	1.529265587	6.42899E-05	upregulated
BIRC3	1.529804783	3.57751E-08	upregulated
CD274	1.530746729	1.13509E-11	upregulated
MUCL3	1.531581963	1.28475E-06	upregulated
ONECUT3	1.545627809	0.000573716	upregulated
SECTM1	1.5538	1.18826E-10	upregulated
FAM167A	1.555951563	4.14614E-09	upregulated
PLA2G2A	1.557744827	4.88647E-06	upregulated
HOXC6	1.559873023	4.79657E-09	upregulated
CXCL10	1.572284554	2.91896E-08	upregulated
SNPH	1.573356919	1.15152E-08	upregulated
DOC2B	1.580743875	0.005483125	upregulated
SPINK4	1.586125418	0.027028855	upregulated
CD55	1.59111045	2.37083E-11	upregulated
KRT13	1.594088784	0.011203638	upregulated
JPX	1.596588192	0.003611694	upregulated
CREB3L1	1.601906543	3.30877E-10	upregulated
AQP5	1.602263011	0.004411358	upregulated
CA8	1.609015631	3.06897E-07	upregulated
SLC7A11.AS1	1.611246884	7.08492E-06	upregulated

DNAH2	1.613192934	2.87835E-08	upregulated
STARD13.AS	1.617492036	0.000866806	upregulated
PDE10A	1.619555608	1.0308E-08	upregulated
COLGALT2	1.622409998	4.72809E-06	upregulated
MUC2	1.626697376	1.15435E-07	upregulated
TNFSF9	1.627358158	4.88304E-11	upregulated
HLA.DOA	1.633872034	0.000259806	upregulated
SERPINA1	1.634308761	0.000377343	upregulated
MUC17	1.638351548	0.005865799	upregulated
GFI1	1.639535606	9.57494E-16	upregulated
GZMA	1.640460248	2.03469E-12	upregulated
PI3	1.651850595	0.022009205	upregulated
TRNP1	1.656385668	1.68416E-11	upregulated
RAB3B	1.659642136	6.51543E-07	upregulated
CTSE	1.687233834	2.22636E-09	upregulated
SLC6A14	1.688384721	0.000287515	upregulated
**ALOX15B**	1.720245015	4.6597E-09	upregulated
TFAP2A	1.725761351	1.14138E-13	upregulated
NTSR1	1.730520616	0.034683946	upregulated
HMX3	1.754129842	3.64125E-05	upregulated
H1.2	1.774421189	0.000249704	upregulated
CP	1.775996994	0.003259945	upregulated
RAMP1	1.792368825	1.76104E-07	upregulated
CXCL9	1.794116931	7.61638E-10	upregulated
ADGRG6	1.798633047	9.31791E-16	upregulated
RAB27B	1.800475737	4.96906E-17	upregulated
PLAAT4	1.812262477	1.00612E-08	upregulated
VNN2	1.83212142	3.17622E-12	upregulated
AGR2	1.836687029	1.31846E-13	upregulated
CD109	1.844986728	2.44513E-05	upregulated
PTMAP4	1.846154303	5.67019E-19	upregulated
ZNF460	1.862554024	2.06851E-06	upregulated
DEFA5	1.899943347	0.000397197	upregulated
ULBP2	1.912616184	7.6287E-12	upregulated
H2BC13	1.934748621	0.000799398	upregulated
CIITA	1.955952273	4.431E-13	upregulated
S100A8	1.96291433	4.5858E-05	upregulated
GBP4	1.964463678	2.71377E-12	upregulated
H2AC20	1.983038758	1.7562E-09	upregulated
MT.RNR1	1.990255308	0.024958432	upregulated
ANKRD36C	1.996156028	0.000476371	upregulated
AQP3	2.004099003	4.71054E-07	upregulated
CPS1	2.015354794	0.000186463	upregulated
RARRES1	2.026671185	8.97997E-10	upregulated
GABRP	2.064693153	2.2426E-10	upregulated
GBP5	2.090585609	7.31725E-09	upregulated
SEMG1	2.091033702	1.08928E-08	upregulated
**IDO1**	2.092411096	1.6082E-06	upregulated
TRIM72	2.103648702	2.80395E-06	upregulated
H3C12	2.105306287	1.19309E-06	upregulated

GNLY	2.136358095	9.86291E-15	upregulated
H2AC13	2.179254832	0.030267749	upregulated
REG1A	2.182970153	0.000276199	upregulated
MT.TM	2.186923753	0.011454647	upregulated
DUSP4	2.192023883	2.24509E-15	upregulated
MUC5B	2.202674987	4.38489E-06	upregulated
H2BC17	2.216177778	0.000729948	upregulated
SNORD17	2.253636348	0.034802182	upregulated
H2BC4	2.263041328	0.002692449	upregulated
DLGAP1.AS5	2.284606039	5.47973E-09	upregulated
NXPH4	2.338751683	9.80036E-09	upregulated
SNORA73B	2.371684716	0.003777305	upregulated
TRPV6	2.434679065	5.66763E-17	upregulated
VNN1	2.436869924	5.23393E-12	upregulated
SPRR3	2.444250304	0.002320697	upregulated
PIWIL1	2.446775055	0.002868499	upregulated
MTND1P23	2.480502228	0.000293265	upregulated
REG4	2.487101853	1.11333E-11	upregulated
TNNT1	2.510992234	1.85722E-13	upregulated
ANXA10	2.584983243	1.06886E-16	upregulated
TRIM7	2.616065137	5.85743E-18	upregulated
RN7SL3	2.673833689	0.000227837	upregulated
SPRR1B	2.717349103	0.046122721	upregulated
H3C2	2.722755176	7.79643E-06	upregulated
**MTND4P24**	2.77591202	0.049743701	upregulated
MUC5AC	2.780578366	2.11856E-08	upregulated
H4C4	2.897029826	0.002652246	upregulated
TFF2	2.899248861	0.000232685	upregulated
AFAP1.AS1	2.937702047	0.000100068	upregulated
PLAC4	2.938821707	0.030707214	upregulated
H1.3	2.954265625	0.007557402	upregulated
RN7SL4P	2.998290247	0.008330301	upregulated
RN7SL1	3.007704129	6.21063E-05	upregulated
KRT16	3.111412869	0.003154294	upregulated
CLDN18	3.120875261	1.11997E-14	upregulated
H1.5	3.127278564	0.008996021	upregulated
H1.4	3.146093979	1.11486E-05	upregulated
GP2	3.501670891	0.002830925	upregulated
KRT14	4.953215725	0.0496796	upregulated
MUC6	5.37657105	9.50319E-12	upregulated
PAEP	5.914348257	0.000860343	upregulated

Supplementary Table S1D TCGA edgeR AA vs. EA DEGs

TCGA DEG	edgeR		African vs. European Ancestry
Gene	logFold change	FDR	Direction
PAEP	-5.018345647	1.24192E-08	downregulated
ORM1	-4.748100505	1.83901E-11	downregulated
A2ML1	-3.224169247	7.45216E-08	downregulated
DLGAP1.AS5	-3.121050055	9.37752E-07	downregulated
CALCB	-3.005395606	0.000140022	downregulated
TMPRSS11E	-2.759248806	2.97329E-06	downregulated
MUC16	-2.730288281	5.6446E-06	downregulated
SYT4	-2.669676377	4.64088E-07	downregulated
CHRNB2	-2.600718559	2.24498E-07	downregulated
INSL5	-2.57905384	3.43285E-06	downregulated
RPS4XP22	-2.555834414	3.49636E-20	downregulated
EEF1A2	-2.535027001	2.04041E-07	downregulated
SFTPB	-2.47000054	2.82166E-05	downregulated
CHGB	-2.418419034	6.57364E-05	downregulated
MT.TM	-2.39546472	5.16754E-05	downregulated
LEP	-2.330806009	4.3343E-05	downregulated
HDGFP1	-2.324534817	5.38572E-11	downregulated
HMX3	-2.323261066	1.17541E-06	downregulated
INSM1	-2.320614877	1.27412E-05	downregulated
SCG3	-2.312299239	1.66711E-06	downregulated
H1.5	-2.30499485	0.001294435	downregulated
AMER3	-2.267346504	2.85179E-06	downregulated
CLDN18	-2.250628321	0.006289633	downregulated
DEFA5	-2.215529622	0.00568619	downregulated
KRT14	-2.169726842	0.005226082	downregulated
SPRR3	-2.169458928	0.001453048	downregulated
NEUROD1	-2.157356124	0.000374978	downregulated
PCDHA5	-2.129572773	2.57275E-06	downregulated
MIR3147HG	-2.068291184	8.11635E-05	downregulated
LINC02154	-2.06406539	7.94761E-08	downregulated
IGHGP	-2.045015653	8.51262E-06	downregulated
EPHB6	-2.031817573	2.22937E-05	downregulated
KCNQ2	-2.028689692	0.000117195	downregulated
CARTPT	-2.026961017	6.88405E-06	downregulated
ASB5	-2.026860678	2.59339E-05	downregulated
MUCL3	-2.017387259	0.000181243	downregulated
FMO2	-1.987612223	5.50147E-07	downregulated
GP2	-1.958575994	0.006184748	downregulated
AP3B2	-1.926389026	7.88763E-05	downregulated
DPYSL5	-1.912743432	4.39896E-05	downregulated
H4C4	-1.904738219	0.004783809	downregulated
KIF1A	-1.904534706	9.11609E-05	downregulated
F11	-1.890665948	1.56883E-05	downregulated
RIMS4	-1.862815281	4.06079E-05	downregulated
TM4SF4	-1.849631738	0.001294221	downregulated
LINC01913	-1.841616063	0.000396767	downregulated
SLC22A31	-1.839872549	0.000378622	downregulated

PGC	-1.826373455	0.000296428	downregulated
UNC13A	-1.826300569	3.6643E-06	downregulated
IGFL1	-1.817470164	0.000566053	downregulated
LINC01923	-1.800163306	0.000196908	downregulated
CLDN8	-1.787649893	0.002759995	downregulated
CACNA1E	-1.787093296	7.32821E-05	downregulated
WNT7A	-1.786248647	0.000538881	downregulated
LCN15	-1.785187905	0.017600987	downregulated
ALOX15B	-1.773002152	1.27565E-05	downregulated
B3GALT5.AS1	-1.767335354	9.87707E-05	downregulated
LRP2	-1.752310148	0.000749565	downregulated
BRINP3	-1.748226563	0.001684922	downregulated
PRG4	-1.743106705	4.75841E-05	downregulated
NKX2.1	-1.728659355	0.005872409	downregulated
**HCAR2**	-1.726617136	5.9108E-05	downregulated
APOB	-1.725471801	0.000680502	downregulated
H1.4	-1.720997674	0.010463447	downregulated
PCOLCE2	-1.719681252	1.66167E-05	downregulated
CRABP1	-1.716016239	3.17558E-05	downregulated
CYP3A4	-1.70830318	0.000222869	downregulated
EMX2	-1.69419547	0.000221728	downregulated
GCG	-1.680396972	0.0018016	downregulated
KRT6C	-1.669028158	0.001486454	downregulated
TCF23	-1.662929976	0.000174209	downregulated
ADIPOQ	-1.655550546	0.01687482	downregulated
MAPK8IP1P2	-1.650212412	0.001458506	downregulated
H3C2	-1.648614946	0.016934921	downregulated
SEZ6	-1.642476782	0.000307086	downregulated
GABRB3	-1.642341103	0.000651092	downregulated
DEFA6	-1.635458172	0.019113509	downregulated
KRT6A	-1.635240389	0.014013154	downregulated
NKAIN2	-1.621280153	0.000391962	downregulated
ORM2	-1.616420442	4.76964E-07	downregulated
RUNDC3A	-1.599261587	0.000105343	downregulated
VGF	-1.592264572	0.000685726	downregulated
DHRS2	-1.586149178	0.000320015	downregulated
NCCRP1	-1.585235502	0.000939819	downregulated
H2BC13	-1.571113782	0.004700068	downregulated
KRT16	-1.561045247	0.006027412	downregulated
IGHV3.13	-1.553470248	0.000516044	downregulated
IBSP	-1.551784358	8.56057E-05	downregulated
CXCL8	-1.549566788	0.000160709	downregulated
XACT	-1.549121324	0.025526982	downregulated
WT1.AS	-1.544109952	0.001209087	downregulated
LRRTM1	-1.540465241	0.004811796	downregulated
PM20D1.AS1	-1.534324107	0.000173016	downregulated
THSD7B	-1.532676305	0.000339771	downregulated
PAX4	-1.529557862	0.00387444	downregulated
LINC02188	-1.52851563	0.000549782	downregulated
HP	-1.526386488	0.002152329	downregulated

ARSLP1	-1.522157828	2.27791E-05	downregulated
LINC02128	-1.509624905	0.000401403	downregulated
RPSAP53	-1.505850823	0.00089003	downregulated
STMN2	-1.48329968	0.000274243	downregulated
PSCA	-1.476745768	0.018320337	downregulated
H1.3	-1.473031244	0.025429885	downregulated
UNC5A	-1.452125368	0.000536612	downregulated
NKAIN1	-1.445100581	6.78682E-06	downregulated
SMIM38	-1.438419215	0.000884057	downregulated
PCAT14	-1.429881249	0.010551755	downregulated
H2BC17	-1.416807905	0.023644692	downregulated
MKRN3	-1.416767595	0.00808205	downregulated
ATP12A	-1.409781739	0.009697469	downregulated
PLA2G2F	-1.406308922	0.005168957	downregulated
LRRN1	-1.39222651	0.000114173	downregulated
KRT6B	-1.391815262	0.00692976	downregulated
KLRG2	-1.391517188	0.001794083	downregulated
SLC5A8	-1.390286343	0.02095666	downregulated
C6orf15	-1.378798131	0.016546801	downregulated
SLC7A14	-1.364121494	0.001679745	downregulated
FABP4	-1.3588431	0.027852389	downregulated
SVOP	-1.354487718	0.002263139	downregulated
PENK	-1.35410818	0.001866488	downregulated
HCAR3	-1.35201848	0.002034267	downregulated
HTATSF1P2	-1.344867471	1.88933E-10	downregulated
ST18	-1.337439065	0.000165233	downregulated
PCDHGA8	-1.336158386	0.003473567	downregulated
SNORD17	-1.335506649	0.024095945	downregulated
ANO3	-1.329413018	0.001666832	downregulated
TPH1	-1.312672492	0.003109677	downregulated
SEZ6L	-1.312561831	0.001914211	downregulated
LHFPL4	-1.302893404	0.013395056	downregulated
EEF1GP1	-1.301532953	0.000174042	downregulated
UGT2B10	-1.298784443	0.0084715	downregulated
IL36G	-1.298343106	0.001375275	downregulated
CREB3L3	-1.294115277	0.00030469	downregulated
SLC26A9	-1.291020544	0.034293959	downregulated
PNRC2P1	-1.286505954	7.18492E-06	downregulated
MMP8	-1.282405032	0.019573399	downregulated
SLITRK5	-1.278216877	0.00378079	downregulated
SPRR2D	-1.275141377	0.044373486	downregulated
CCDC144A	-1.274330768	0.006683208	downregulated
CXCL10	-1.274315868	7.34997E-05	downregulated
SLAMF9	-1.266756169	3.90536E-05	downregulated
BOK.AS1	-1.265913298	0.018962315	downregulated
H4C5	-1.265616391	0.026629961	downregulated
RPL10P3	-1.258265211	1.04296E-09	downregulated
**IDO1**	-1.254800567	0.001031186	downregulated
H2AC13	-1.254678925	0.022815194	downregulated
CCK	-1.254041529	0.018143168	downregulated

H2AC20	-1.250676359	0.006940943	downregulated
PHYHIPL	-1.249878353	0.00790326	downregulated
DIPK1C	-1.242558929	0.001382926	downregulated
EPYC	-1.241176484	0.015627376	downregulated
XKR9	-1.240240983	0.000469811	downregulated
IGKV1D.12	-1.231728879	0.011605172	downregulated
KIRREL2	-1.229568813	0.000903593	downregulated
CP	-1.227076965	0.005092492	downregulated
TDRD9	-1.226631062	0.000453574	downregulated
MARCO	-1.22576224	0.006527103	downregulated
ST8SIA6.AS1	-1.222934024	0.022221424	downregulated
EIF5AL1	-1.222365446	3.90673E-05	downregulated
DIRAS2	-1.219589523	0.000637239	downregulated
SERPINA6	-1.219069338	0.004420274	downregulated
ASB12	-1.212278693	1.00209E-07	downregulated
GJB6	-1.21120105	0.004002912	downregulated
ANK1	-1.21044091	1.12594E-05	downregulated
IGHV3.53	-1.204167635	0.007910623	downregulated
SLN	-1.202742535	0.00088843	downregulated
SSTR5.AS1	-1.202678717	0.007575462	downregulated
PLIN1	-1.200530778	0.020946168	downregulated
CDK5R2	-1.198853614	0.004047376	downregulated
NFIA.AS2	-1.196725716	0.003294938	downregulated
SLITRK3	-1.196390823	0.004434569	downregulated
COLEC10	-1.186310561	0.004051487	downregulated
TCAP	-1.182529943	0.009591315	downregulated
GRM4	-1.181690144	0.014862121	downregulated
PLIN4	-1.179215158	0.037835058	downregulated
PCDHA4	-1.174483228	0.009896754	downregulated
ADGRG2	-1.165367469	0.002952417	downregulated
PCSK2	-1.164600703	0.014645802	downregulated
SYNPR	-1.155427052	0.018367617	downregulated
SCEL	-1.148796012	0.013187229	downregulated
PPP1R1A	-1.148612516	0.003048045	downregulated
TFF1	-1.148291876	0.013010712	downregulated
ENTHD1	-1.147540083	0.000758889	downregulated
SNORD3A	-1.144990598	0.0478217	downregulated
LBP	-1.143779344	0.008334577	downregulated
GLYATL2	-1.142586324	0.004619515	downregulated
SHISA9	-1.141182626	0.04222179	downregulated
XIRP1	-1.139082427	0.000244232	downregulated
IGLC7	-1.133617745	0.013392334	downregulated
LINC02560	-1.133195389	0.001581869	downregulated
HOMER2	-1.13122949	0.00131134	downregulated
LINC01980	-1.128788637	0.035527651	downregulated
IL36RN	-1.126448922	0.011211104	downregulated
NEUROG3	-1.126331922	0.012802822	downregulated
COLGALT2	-1.12627925	0.01104587	downregulated
SEMA3D	-1.113523738	0.005226082	downregulated
FAHD2P1	-1.113084898	0.000304239	downregulated

SPESP1	-1.111544644	0.003193698	downregulated
IFNG	-1.111479035	0.000332437	downregulated
LMX1B	-1.109206335	0.007095104	downregulated
CNTN1	-1.109014589	0.00452504	downregulated
LINC02575	-1.104101166	0.028754661	downregulated
HSPA6	-1.103249987	0.001032472	downregulated
LINC00702	-1.098129106	0.000549782	downregulated
ECEL1	-1.097747036	0.002150047	downregulated
TCHH	-1.096446881	0.029000371	downregulated
LINC02765	-1.094977998	0.002484584	downregulated
IGLV3.10	-1.08821068	0.023602179	downregulated
NOS1	-1.086342107	0.007544073	downregulated
MYOC	-1.084305258	0.02207241	downregulated
OGDHL	-1.082026485	0.004374175	downregulated
GZMH	-1.079665479	0.000209414	downregulated
SERPINA10	-1.078408515	0.013670816	downregulated
FBLL1	-1.076725972	0.001862677	downregulated
EVX2	-1.074080271	0.006401096	downregulated
HOXB13	-1.073611791	0.001083673	downregulated
MYBPC1	-1.0734769	0.004076609	downregulated
NLRP2	-1.072714612	0.04859866	downregulated
ATCAY	-1.069023238	0.004889686	downregulated
MTCO2P12	-1.067103603	0.012798213	downregulated
AGAP7P	-1.066900302	0.002473633	downregulated
SLITRK4	-1.066806847	0.006469237	downregulated
LINC00958	-1.062753007	0.005598419	downregulated
**OR2I1P**	-1.060696085	0.002024378	downregulated
VSIG1	-1.053653921	0.020422981	downregulated
CLEC6A	-1.051750203	0.002529613	downregulated
LINC01630	-1.050477773	0.035486643	downregulated
TENM1	-1.047095947	0.014245568	downregulated
ZNF683	-1.04574958	0.002965364	downregulated
RRAS2P1	-1.044431388	0.004022693	downregulated
SLC5A7	-1.043690972	0.015310379	downregulated
SLIT1	-1.040143812	0.006580294	downregulated
ZNF492	-1.039760952	0.012324146	downregulated
FYB2	-1.038216745	0.011085146	downregulated
IGHG4	-1.03693432	0.044472167	downregulated
ZNF750	-1.035751059	0.022883682	downregulated
SUGCT.AS1	-1.031639117	0.00254209	downregulated
RBFOX1	-1.031491142	0.016755073	downregulated
GABRQ	-1.027787047	0.003320639	downregulated
SERPINB2	-1.027551492	0.04854967	downregulated
H3C14	-1.024863119	0.014131476	downregulated
LINC00665	-1.019788473	0.005529327	downregulated
HRK	-1.012817211	0.023803373	downregulated
CSF2	-1.010994815	0.00343167	downregulated
H4C8	-1.010416721	0.004268507	downregulated
CXCL11	-1.010001449	0.004333615	downregulated
DCSTAMP	-1.0067048	0.003021121	downregulated

C5orf58	-1.005781614	0.01853195	downregulated
H2BC7	-1.004037258	0.047568797	downregulated
SP8	-1.000286689	0.040120444	downregulated
MRFAP1P1	1.002879191	3.37507E-10	upregulated
SULT1E1	1.015562845	0.018046657	upregulated
OR5BA1P	1.018520668	8.55682E-06	upregulated
IGKV2D.29	1.0206221	0.005848343	upregulated
NELL1	1.020830568	8.48779E-05	upregulated
IGHV3.20	1.021484091	0.005013187	upregulated
LINC02397	1.027757456	0.000464401	upregulated
SLC7A11.AS1	1.029719291	0.011803172	upregulated
MIR23AHG	1.031100771	6.95363E-10	upregulated
ACSM1	1.032227298	1.42789E-09	upregulated
NIBAN3	1.032740071	0.000189027	upregulated
LINC01237	1.037039036	8.09538E-13	upregulated
AP1G2.AS1	1.037114478	3.33903E-08	upregulated
CR2	1.042117011	0.008605823	upregulated
FCRL1	1.042241322	0.000912038	upregulated
SH2D6	1.042900858	0.000291968	upregulated
CYP2C9	1.044638158	0.000515508	upregulated
IL1RL1	1.050839179	6.34331E-06	upregulated
IGHV3.64D	1.051497537	0.017318404	upregulated
IGLV9.49	1.051577833	0.00902234	upregulated
STPG3.AS1	1.062111672	5.99329E-08	upregulated
WASH7P	1.065465934	1.26539E-13	upregulated
AADAC	1.06656269	0.0004918	upregulated
STAG3L5P	1.067952763	2.59869E-13	upregulated
AGAP10P	1.069183783	9.13309E-12	upregulated
RPS26P15	1.071204576	1.71266E-08	upregulated
TSIX	1.071464813	0.000527476	upregulated
LNC.LBCS	1.075312694	1.08173E-05	upregulated
SLC6A16	1.087850629	1.10004E-07	upregulated
CDH4	1.092350503	1.81478E-08	upregulated
PLCXD3	1.09423348	0.001089795	upregulated
TRPC6P2	1.094726065	0.001823494	upregulated
AKR1B15	1.095806018	0.000153547	upregulated
LMNTD2.AS1	1.095858166	9.4301E-09	upregulated
DNAH10	1.10046526	5.72108E-09	upregulated
LINC03126	1.105603992	2.98759E-07	upregulated
MIR142HG	1.10965127	1.19286E-06	upregulated
TTLL3	1.112436988	1.72369E-13	upregulated
LINC01807	1.11754286	0.000146824	upregulated
MAMDC4	1.130423787	2.39259E-10	upregulated
CACNA1I	1.132275438	3.5036E-06	upregulated
YJEFN3	1.13318342	5.08008E-10	upregulated
PPIAP29	1.139671412	6.70707E-06	upregulated
MSLNL	1.141521212	0.000252461	upregulated
IGHD	1.144363259	0.001069933	upregulated
RPL29P26	1.145437067	3.85206E-07	upregulated
STPG3	1.154938702	3.37507E-10	upregulated

POLR2J3.1	1.155806343	4.45765E-07	upregulated
RLBP1	1.158490771	8.32622E-05	upregulated
GJB7	1.160766022	0.002017324	upregulated
TDO2	1.160889301	2.53146E-05	upregulated
CRYBB2	1.166868405	2.71829E-11	upregulated
SPDYE2	1.169415259	7.08408E-06	upregulated
IGHV1.69.2	1.1751356	0.008383129	upregulated
MTND4P35	1.175363518	0.00011161	upregulated
CD300LD.AS1	1.175837556	0.00114277	upregulated
POLR2J2	1.182673652	2.7583E-09	upregulated
ACAD9.DT	1.185119967	2.16678E-09	upregulated
GAS6.AS1	1.185909603	3.52413E-07	upregulated
SERPIND1	1.186676247	0.000358826	upregulated
GPC1.AS1	1.190275783	4.18273E-06	upregulated
FAM86MP	1.191001316	4.35171E-07	upregulated
ARHGEF38.IT1	1.195183491	0.000137105	upregulated
COX6CP1	1.20570569	9.36429E-05	upregulated
ESPNP	1.2069494	5.06167E-10	upregulated
KRT1	1.212628852	1.04508E-05	upregulated
CCDC154	1.213902933	1.88612E-11	upregulated
PRH2	1.219458456	0.000320015	upregulated
IGKV1D.43	1.230383001	3.07871E-05	upregulated
BHLHA9	1.23960456	0.000160302	upregulated
CFAP276	1.242889991	3.85499E-08	upregulated
TMEM132C	1.24314254	0.006053733	upregulated
AIRE	1.246490767	1.82194E-05	upregulated
EVX1.AS	1.251053457	1.34304E-05	upregulated
LINGO4	1.251512992	2.6312E-08	upregulated
SLC4A10	1.275146465	0.000111901	upregulated
SFRP5	1.277402966	0.00011161	upregulated
GNG13	1.287115533	3.22059E-06	upregulated
SPIN2A	1.287709001	0.000726392	upregulated
GREP1	1.288649756	6.43318E-05	upregulated
MT.ND5	1.290058528	2.70012E-10	upregulated
SYCP2	1.291660401	2.8324E-07	upregulated
CSTL1	1.298534889	8.01763E-10	upregulated
GPC3	1.299486024	5.89968E-06	upregulated
PWP2	1.30367949	2.14971E-08	upregulated
XIST	1.309848833	0.020280825	upregulated
APPAT	1.311687802	7.11176E-12	upregulated
MTND6P4	1.316195735	4.06008E-05	upregulated
SUZ12P1	1.326404466	1.6089E-19	upregulated
DKK4	1.336869795	0.002849426	upregulated
LERFS	1.343080673	4.13085E-06	upregulated
TCL1A	1.343532476	0.000143407	upregulated
NXNL2	1.352274093	1.68549E-15	upregulated
LRRC36	1.353375786	1.351E-10	upregulated
CROCC2	1.374530766	9.11213E-08	upregulated
VWFP1	1.377275011	2.13109E-09	upregulated
IGFN1	1.386203321	1.91933E-05	upregulated

LINC01709	1.388159632	3.59245E-05	upregulated
POLR2J3	1.390811673	7.20309E-27	upregulated
PON1	1.391114458	2.64171E-06	upregulated
NPIPB15	1.405800743	2.75715E-08	upregulated
HK2P1	1.409473619	3.16145E-14	upregulated
MICOS10P3	1.411245908	2.09834E-06	upregulated
LINC02350	1.412955218	0.000336645	upregulated
OBP2B	1.422436591	0.000251169	upregulated
NXF3	1.434847336	0.000322668	upregulated
LINC02512	1.435510628	7.81638E-07	upregulated
IGHJ6	1.440182366	0.000124165	upregulated
APOA1	1.440903636	1.65169E-05	upregulated
EEF1A1P13	1.461562857	5.46329E-12	upregulated
RPL29P11	1.462917173	1.21816E-09	upregulated
TCL6	1.47285805	2.87538E-06	upregulated
FLG	1.488711161	2.69493E-07	upregulated
LINC03033	1.494079078	8.88506E-16	upregulated
FAM238C	1.50753091	1.78853E-13	upregulated
RBM44	1.516740614	1.92923E-15	upregulated
RAMACL	1.522696585	1.84555E-10	upregulated
MTCYBP18	1.547401114	9.66918E-06	upregulated
RPL15P18	1.573987989	1.52823E-14	upregulated
IGLV5.37	1.576250832	8.2908E-07	upregulated
CALR4P	1.577868696	1.45138E-09	upregulated
RBP3	1.578244208	3.93404E-07	upregulated
ATP5MFP2	1.621515525	2.20652E-10	upregulated
CTNNA2	1.636342573	2.59339E-05	upregulated
MT.ND6	1.638749038	9.82831E-14	upregulated
GATD3	1.640651354	4.68289E-13	upregulated
SIK1	1.647644957	6.84253E-11	upregulated
DCT	1.653648973	2.06908E-06	upregulated
RARA.AS1	1.687064314	8.73594E-25	upregulated
MTND4P20	1.706427661	1.90292E-07	upregulated
IGKV1D.13	1.710148414	1.81478E-08	upregulated
IGLV7.46	1.716524313	3.45643E-06	upregulated
SORD2P	1.73489972	4.87678E-25	upregulated
KRT39	1.737947791	2.0934E-08	upregulated
RN7SL5P	1.766527428	3.10717E-08	upregulated
GCAWKR	1.840359168	1.15345E-24	upregulated
PSPHP1	1.844734628	1.52344E-07	upregulated
CYP4F8	1.896057053	5.29998E-11	upregulated
HSD3B2	1.936874741	2.39259E-10	upregulated
RPL10P6	1.965067056	4.26E-10	upregulated
UICLM	1.991356978	3.1901E-12	upregulated
ZBTB8OSP2	2.006221487	4.17415E-27	upregulated
ID2.AS1	2.013507667	1.68119E-27	upregulated
IGKV2.29	2.046173527	4.0503E-10	upregulated
RPS26P47	2.055938611	4.08658E-16	upregulated
IGHV3.64	2.084706658	5.72108E-09	upregulated
MTCO1P12	2.128394953	3.34201E-35	upregulated

PPBP	2.184283352	0.000351075	upregulated
IGKV1D.27	2.205510236	9.70973E-17	upregulated
MTND4P12	2.312411196	8.37325E-12	upregulated
MTND5P11	2.35143615	7.03013E-16	upregulated
ARHGAP40	2.443028139	6.49231E-22	upregulated
PRSS2	2.520573591	3.22059E-06	upregulated
MTCO1P2	2.523096487	2.7162E-37	upregulated
RPL10P9	2.526233506	1.70286E-22	upregulated
TDRD12	2.622368329	1.52882E-45	upregulated
MTCO1P53	2.628336371	4.23791E-16	upregulated
LINC00632	2.705886502	1.30106E-13	upregulated
MTCO3P22	2.770524497	3.11926E-32	upregulated
HNRNPA3P11	2.800168375	2.40956E-21	upregulated
CIST1	2.957852869	1.37416E-32	upregulated
PNMT	3.023181522	6.23031E-28	upregulated
COL2A1	3.083636356	4.82764E-18	upregulated
IGLVI.70	3.511474159	4.45224E-39	upregulated
PRSS56	3.531928923	2.69283E-17	upregulated
**MTND4P24**	3.66376244	6.29655E-28	upregulated
MTATP6P2	4.339946284	2.34222E-65	upregulated
MTCO1P40	4.470297181	9.2117E-48	upregulated
MTND1P23	4.984571581	5.53902E-29	upregulated
**MTATP8P2**	5.566008937	5.82612E-92	upregulated

Supplementary Table S1E TCGA Wilcoxon AA vs. EA DEGs

TCGA DEG	Wilcoxon		African vs. European ancesty
Gene	logFold change	FDR	Direction
IGHGP	-2.006718269	0.000123984	downregulated
XACT	-1.459214211	0.046792998	downregulated
**CXCL10**	-1.203114943	0.001478075	downregulated
PSCA	-1.203105057	0.038763083	downregulated
**ALOX15B**	-1.160771322	0.003245156	downregulated
**IDO1**	-1.147256252	0.003978534	downregulated
RPS4XP22	-1.125714181	3.70278E-16	downregulated
**HCAR2**	-1.026191707	0.025286811	downregulated
**OR2I1P**	-1.017690159	0.027720673	downregulated
**MARCO**	-1.01209827	0.000868186	downregulated
MIR23AHG	1.006918423	0.000125982	upregulated
MTCYBP18	1.031616034	0.020976313	upregulated
MAMDC4	1.042360701	0.000438313	upregulated
TTLL3	1.057589567	7.38201E-07	upregulated
GATD3	1.080083952	1.33331E-09	upregulated
IGKV2.29	1.091254921	6.46589E-05	upregulated
CIST1	1.105020144	0.004118726	upregulated
GAS6.AS1	1.135507044	0.000156395	upregulated
LRRC36	1.155161196	0.000519366	upregulated
SIK1	1.182411085	2.81233E-09	upregulated
NPIPB15	1.188868271	5.6377E-11	upregulated
SUZ12P1	1.236115034	0.02280582	upregulated
IGLVI.70	1.252852431	9.52607E-06	upregulated
MT.ND5	1.289646578	0.001900697	upregulated
SORD2P	1.482683885	5.88167E-08	upregulated
MTND5P11	1.498158739	4.69045E-09	upregulated
PSPHP1	1.544843762	1.87507E-15	upregulated
MTCO1P53	1.586209227	9.70705E-11	upregulated
IGHV3.64	1.623468884	0.002270223	upregulated
MT.ND6	1.637354124	0.020916655	upregulated
RPL10P6	1.703167396	2.06721E-11	upregulated
ID2.AS1	1.772227107	6.12957E-09	upregulated
MTCO1P12	2.11218152	9.38731E-13	upregulated
MTND4P12	2.288952023	9.27896E-11	upregulated
RPL10P9	2.403094896	3.31626E-13	upregulated
**MTND4P24**	2.600486948	2.3153E-22	upregulated
MTATP8P2	3.630291198	3.39657E-12	upregulated
MTCO1P40	4.026500751	3.67765E-10	upregulated
MTND1P23	4.828086083	3.12458E-11	upregulated
