# Supplementary figures and images for: The effect of African ancestry and mismatch-repair enzyme deficiency/microsatellite instability-high on colorectal adenocarcinoma immune gene expression
Source: Front Gastroenterol (Lausanne). 2025 Oct 3;4:1638438. doi: 10.3389/fgstr.2025.1638438 (PMC12867126; doi:10.3389/fgstr.2025.1638438)

# TCGA-COREAD: CXCL10 vs Total T Cell Infiltration

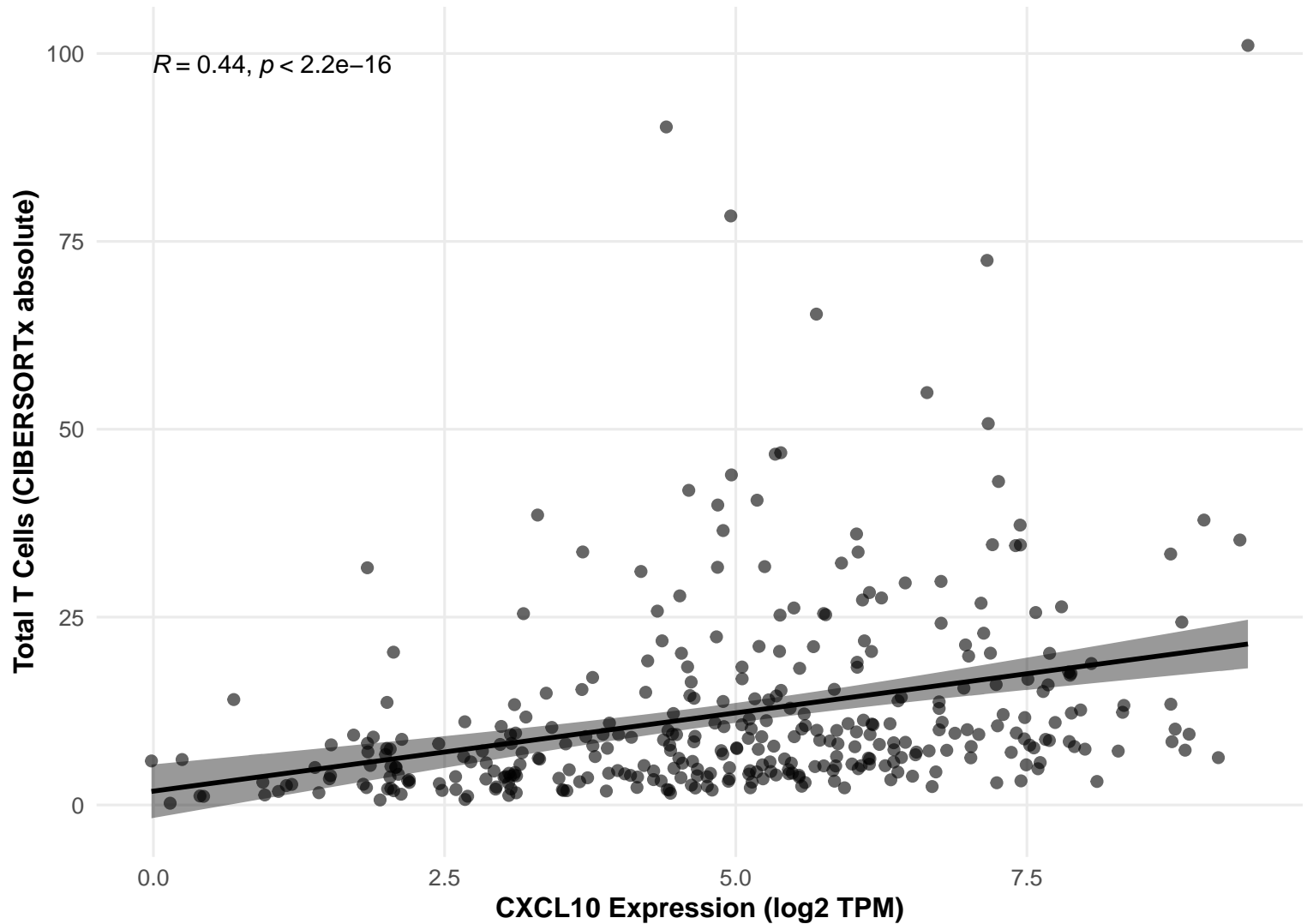

Supplement: Supplementary Figure 2 — Scatter plot of CXCL10 log2TPM values in (A) AA CMS1, CMS2, CMS3, CMS4 and No Labels groups; and (B) EA CMS1, CMS2, CMS3, CMS4 and No Labels groups. [file DataSheet2.pdf]
