# Supplementary material for: The effect of African ancestry and mismatch-repair enzyme deficiency/microsatellite instability-high on colorectal adenocarcinoma immune gene expression
Source: Front Gastroenterol (Lausanne). 2025 Oct 3;4:1638438. doi: 10.3389/fgstr.2025.1638438 (PMC12867126; doi:10.3389/fgstr.2025.1638438)

Supplementary Figure S2. Comparison of *CXCL10* log_2TPM values between (A) CMS1, CMS2, CMS3, CMS4, NO LABEL in AA TCGA-COAD-READ; (B) CMS1, CMS2, CMS3, CMS4, NO LABEL in EA TCGA-COAD-READ.

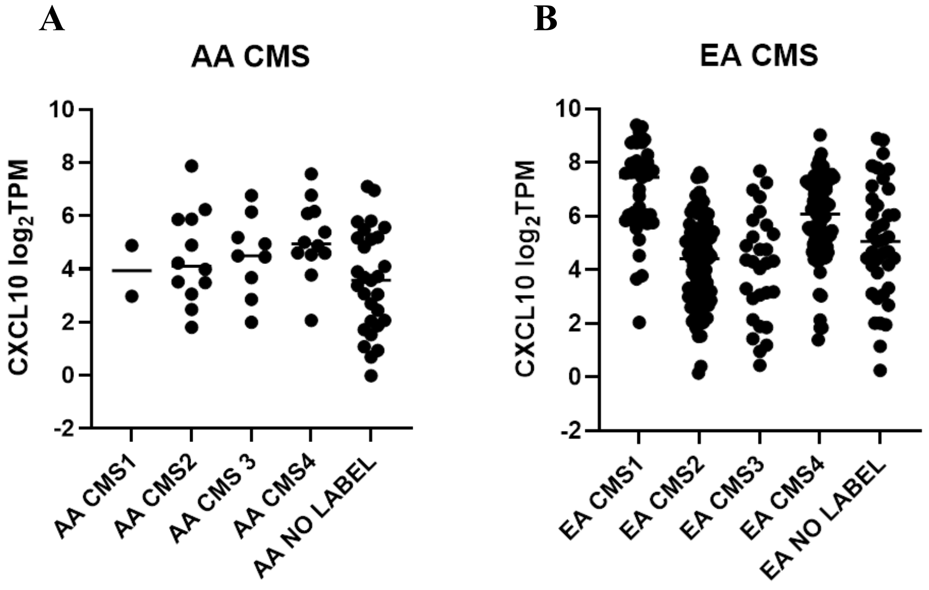

Supplement: Supplementary file 3 [file DataSheet3.pdf]
