# Supplementary material for: The effect of African ancestry and mismatch-repair enzyme deficiency/microsatellite instability-high on colorectal adenocarcinoma immune gene expression
Source: Front Gastroenterol (Lausanne). Author manuscript; Available in PMC 2026 Feb 4. (PMC12867126; doi:10.3389/fgstr.2025.1638438)
Supplement: Supplementary file 1 [file NIHMS2139346-supplement-Supplementary_Figure_1.pdf]

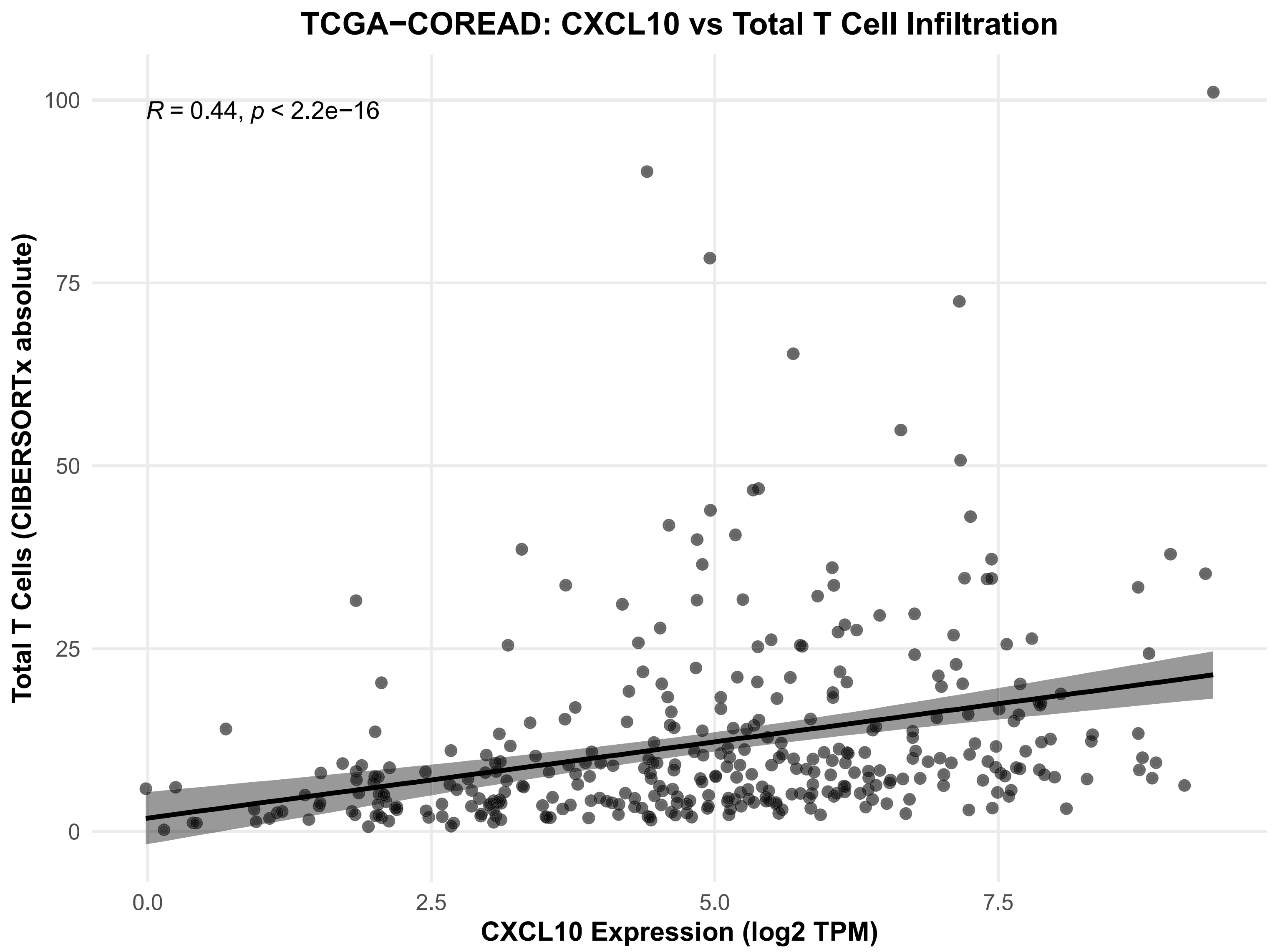
TCGA−COREAD: CXCL10 vs Total T Cell Infiltration
R = 0.44, p < 2.2e−16
Total T Cells (CIBERSORTx absolute)
100
75
50
25
0
0.0
2.5
5.0
7.5
CXCL10 Expression (log2 TPM)
